# Plant Biosystems Design Research Roadmap 1.0

**DOI:** 10.34133/2020/8051764

**Published:** 2020-12-05

**Authors:** Xiaohan Yang, June I. Medford, Kasey Markel, Patrick M. Shih, Henrique C. De Paoli, Cong T. Trinh, Alistair J. McCormick, Raphael Ployet, Steven G. Hussey, Alexander A. Myburg, Poul Erik Jensen, Md Mahmudul Hassan, Jin Zhang, Wellington Muchero, Udaya C. Kalluri, Hengfu Yin, Renying Zhuo, Paul E. Abraham, Jin-Gui Chen, David J. Weston, Yinong Yang, Degao Liu, Yi Li, Jessy Labbe, Bing Yang, Jun Hyung Lee, Robert W. Cottingham, Stanton Martin, Mengzhu Lu, Timothy J. Tschaplinski, Guoliang Yuan, Haiwei Lu, Priya Ranjan, Julie C. Mitchell, Stan D. Wullschleger, Gerald A. Tuskan

**Affiliations:** ^1^Biosciences Division, Oak Ridge National Laboratory, Oak Ridge, TN 37831, USA; ^2^The Center for Bioenergy Innovation, Oak Ridge National Laboratory, Oak Ridge, TN 37831, USA; ^3^Department of Biology, Colorado State University, Fort Collins, CO 80523, USA; ^4^Department of Plant Biology, University of California, Davis, Davis, CA, USA; ^5^Feedstocks Division, Joint BioEnergy Institute, Emeryville, CA, USA; ^6^Department of Biodesign, Biological Systems and Engineering Division, Lawrence Berkeley National Laboratory, Berkeley, CA, USA; ^7^Department of Chemical and Biomolecular Engineering, University of Tennessee, Knoxville, TN 37996, USA; ^8^SynthSys and Institute of Molecular Plant Sciences, School of Biological Sciences, University of Edinburgh, Edinburgh EH9 3BF, UK; ^9^Department of Biochemistry, Genetics and Microbiology, Forestry and Agricultural Biotechnology Institute (FABI), University of Pretoria, Pretoria 0002, South Africa; ^10^Department of Food Science, University of Copenhagen, Rolighedsvej 26, DK-1858, Frederiksberg, Copenhagen, Denmark; ^11^State Key Laboratory of Subtropical Silviculture, School of Forestry and Biotechnology, Zhejiang A&F University, Hangzhou, Zhejiang 311300, China; ^12^State Key Laboratory of Tree Genetics and Breeding, Research Institute of Subtropical Forestry, Chinese Academy of Forestry, Hangzhou, Zhejiang 311400, China; ^13^Department of Plant Pathology and Environmental Microbiology and the Huck Institute of the Life Sciences, The Pennsylvania State University, University Park, PA 16802, USA; ^14^Department of Genetics, Cell Biology and Development, Center for Precision Plant Genomics and Center for Genome Engineering, University of Minnesota, Saint Paul, MN 55108, USA; ^15^Department of Plant Science and Landscape Architecture, University of Connecticut, Storrs, CT 06269, USA; ^16^Division of Plant Sciences, Bond Life Sciences Center, University of Missouri, Columbia, MO, USA; ^17^Donald Danforth Plant Science Center, St. Louis, MO, USA; ^18^Environmental Sciences Division, Oak Ridge National Laboratory, Oak Ridge, TN 37831, USA

## Abstract

Human life intimately depends on plants for food, biomaterials, health, energy, and a sustainable environment. Various plants have been genetically improved mostly through breeding, along with limited modification via genetic engineering, yet they are still not able to meet the ever-increasing needs, in terms of both quantity and quality, resulting from the rapid increase in world population and expected standards of living. A step change that may address these challenges would be to expand the potential of plants using biosystems design approaches. This represents a shift in plant science research from relatively simple trial-and-error approaches to innovative strategies based on predictive models of biological systems. Plant biosystems design seeks to accelerate plant genetic improvement using genome editing and genetic circuit engineering or create novel plant systems through *de novo* synthesis of plant genomes. From this perspective, we present a comprehensive roadmap of plant biosystems design covering theories, principles, and technical methods, along with potential applications in basic and applied plant biology research. We highlight current challenges, future opportunities, and research priorities, along with a framework for international collaboration, towards rapid advancement of this emerging interdisciplinary area of research. Finally, we discuss the importance of social responsibility in utilizing plant biosystems design and suggest strategies for improving public perception, trust, and acceptance.

## 1. Introduction

**Figure 1:**
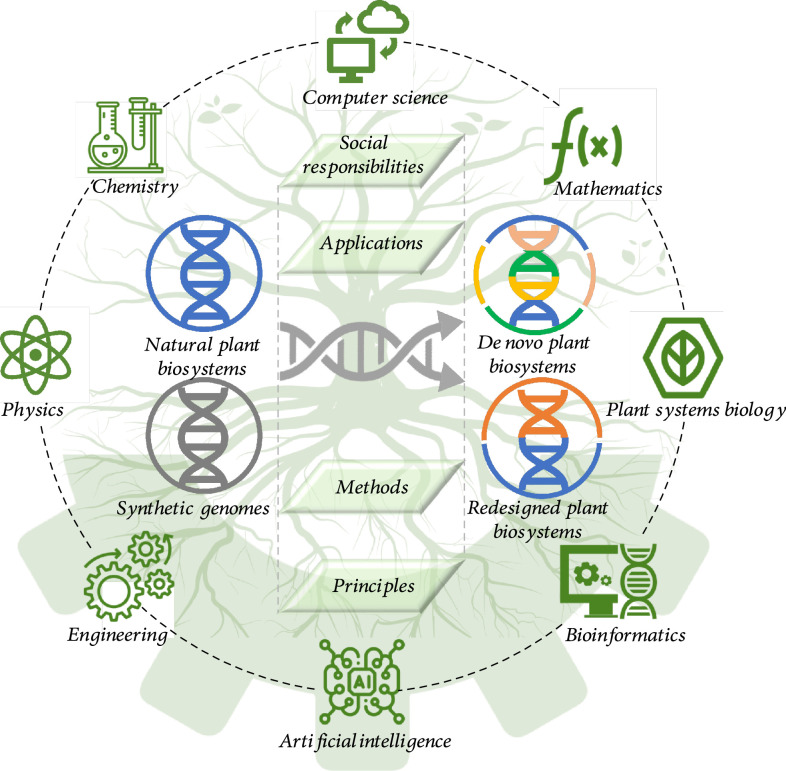
The scope of plant biosystems design research. Plant biosystems design is an interdisciplinary research field integrating plant systems biology, engineering, chemistry, computer science, bioinformatics, artificial intelligence, physics, and mathematics to redesign natural plant systems and construct new plant systems in a predictable and programmable manner. Plant biosystems design research covers four aspects: theories and principles, methods and toolboxes, applications, and social responsibilities. Some icon made by Pixel perfect from http://www.flaticon.com/ and http://www.pngtree.com/.

Humans depend on plants for a variety of important resources including sustenance, energy, clothing, bio-based products, and shelter [1–3]. On a global scale, plants play critical roles in biogeochemical cycling and environmental stability [4, 5]. There are currently ~374,000 known plant species on Earth, of which approximately 82% are vascular plants [6], and only a small fraction of these have been domesticated. There still exists vast potential for incorporating useful traits from wild relatives and from natural plant populations to design plants for human and environmental use. It has become increasingly clear that the current trajectories of yield increase for staple crop varieties/cultivars will not be adequate to meet the future demands of the increasing global population [7, 8]. Furthermore, many crop plants may not be sufficiently robust to cope with impending stresses of rapid climate change such as extreme weather, reduced water resources (e.g., reduction in both quantity due to drought and quality due to pollution), and deteriorated soil quality [9, 10]. Therefore, there is an urgent need for new strategies to accelerate crop development and domestication and expand the possibilities for a thriving plant-based bioeconomy (e.g., production of novel bio-based products) to address our projected economic, social, and environmental needs. To this end, a new frontier in plant research called “plant biosystems design” is emerging and quickly evolving. Plant biosystems design is an interdisciplinary field of research that seeks to genetically/epigenetically improve plants or create novel plant traits or organisms through editing, engineering and refactoring of native, heterologous, or synthetic biological parts based on predictive design (Figure 1). To promote this emerging field, we present a roadmap for plant biosystems design that aims to identify knowledge gaps, technical challenges, and opportunities. We review theoretical and technical approaches and propose innovative applications of biosystems design for basic and applied plant science research, along with strategies to enhance social responsibility of scientists and companies in terms of biosafety and ethics (e.g., public beneficence, intellectual freedom and responsibility, and fairness).

## 2. Theoretical Approaches and Principles of Plant Biosystems Design

Plants are complex, multicellular organisms. The predictive design of plant biosystems requires a comprehensive understanding of the principles underlying biological processes across all scales, from molecular interactions to cellular metabolism, cell/tissue/organ growth and development, and environmental responses of plants. Plant biosystems design involves several theoretical approaches: (1) graph theory providing a graphic view of the structure of plant systems, (2) mechanistic models linking genes to phenotypic traits, and (3) evolutionary dynamics theory enabling prediction of the genetic stability and evolvability of genetically modified plants or *de novo* plant systems. These theoretical approaches enable the design of complex plant systems based on the principles of modular design, dynamic programming, natural and artificial selection (i.e., selective breeding), genetic stability, and upgradability.

### 2.1. Theoretical Approaches

#### 2.1.1. The Graph Theory Approach for Plant Biosystems Design

**Figure 2:**
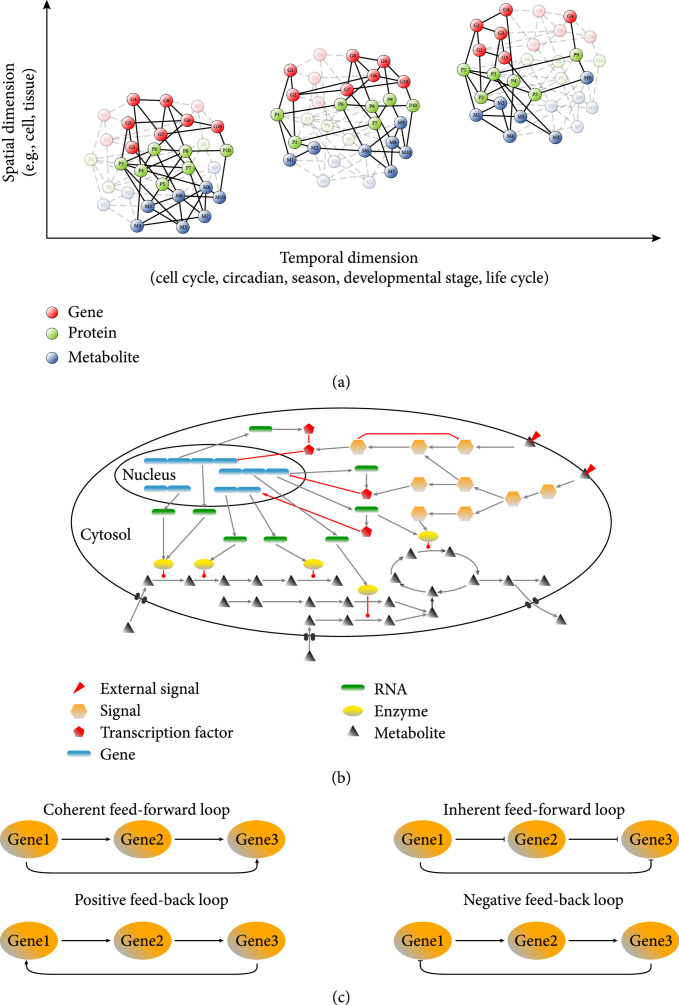
The network control theory of plant biosystems design. (a) Dynamic networks of genes and metabolites distributed in spatial (e.g., cell and tissue) and temporal (e.g., cell cycle, circadian time, season, developmental stage, and life cycle) dimensions. (b) A plant gene-metabolite network; arrow-shaped edges represent activation; blunt edges represent inhibition, and edges ending with a solid circle indicate enzymatic catalysis; adapted from Gonçalves et al. [370]. (c) The structure of regulatory/signaling network motifs; arrows indicate positive regulation; T-shape arrows indicate negative regulation; adapted from Gupta and Singh [20].

A graph can be used to describe complex biological systems where the components and interactions of the system are represented by thousands of nodes (e.g., genes and metabolites) connected with thousands of edges (e.g., interactions) [11]. Inherent to the graph theoretic approach for describing biological systems is the use of network graphs to represent, for example, the extensive communication between metabolic and gene regulatory networks [12]. Metabolites can regulate protein activity via allosteric regulation and posttranslational modifications [13], and gene expression in plants is subject to epigenetic regulation mediated by metabolic fluxes and cellular redox states [14, 15]. From the perspective of biosystems design, a plant biosystem can be defined as a dynamic network of genes and multiple intermediate molecular phenotypes, such as proteins and metabolites, distributed in a four-dimensional space: three spatial dimensions of structure (e.g., cell and tissue) and one temporal dimension (e.g., cell cycle, circadian time, season, developmental stage, and life cycle) (Figure 2(a)). Along the spatial dimensions, plant tissue/organ growth and development are precisely orchestrated in a distributed fashion through collective interactions of many connected cells [16, 17], and therefore, the subnetworks spatially distributed in individual cells are interconnected as the nodes of tissue/organ-scale networks. Furthermore, tissue/organ-scale subnetworks are interconnected as nodes of whole-plant-scale networks. Along the temporal dimension, genes are turned on and off at various time scales, and their expression profiles vary with changes in cell cycle, circadian clock, growing season, development stage, and life cycle. Also, the products of gene expression (RNAs and proteins) are degraded at various time scales, resulting in variation in their turnover times.

A plant gene-metabolite network contains nodes and edges, where the nodes are genes/RNAs/proteins/metabolites, and the edges represent either promotional or inhibitory relationships in protein-protein, protein-RNA, protein-DNA, protein-metabolite, and RNA-RNA interactions (Figure 2(b)). Moreover, the overall gene-metabolite network of a plant biosystem is complex and can be divided into subnetworks responsible for plant growth, development, and interaction with abiotic and biotic environmental factors. Within the subnetworks, there are network motifs that are statistically overrepresented subgraphs as the simple building blocks of complex systems [18, 19]. The structure of regulatory network motifs can be classified as feed-forward loops or feed-back loops (Figure 2(c)) [20, 21]. So far, plant biosystems design has been limited to subnetworks and basic network motifs (e.g., basic loops). There are significant challenges to be addressed: (1) construction of a genome-scale, metabolic/regulatory network with labelled subnetworks responsible for individual biological processes in relation to plant growth, development, and response to the environment; (2) mathematical modeling of this genome-scale network for accurate prediction of plant phenotypes in response to genetic and environmental perturbations; (3) sharing of this consensus predictive model with the scientific community for plant biosystems design; (4) a lack of knowledge of how the metabolic and genetic networks are linked with each other, such as the binding of metabolites to enzymes and transcription factors, although very good progress has been made in the identification of protein-protein or protein-DNA interactions [22]; and (5) insufficient data about the concentration of metabolites in different compartments and different cell types, as well as the transport between different compartments and cell types, which provides a challenge for modeling. New computational tools like MAGI [23] will be needed to facilitate the integration of metabolic and genetic networks.

#### 2.1.2. The Mechanistic Modeling Theory of Plant Biosystems Design

**Figure 3:**
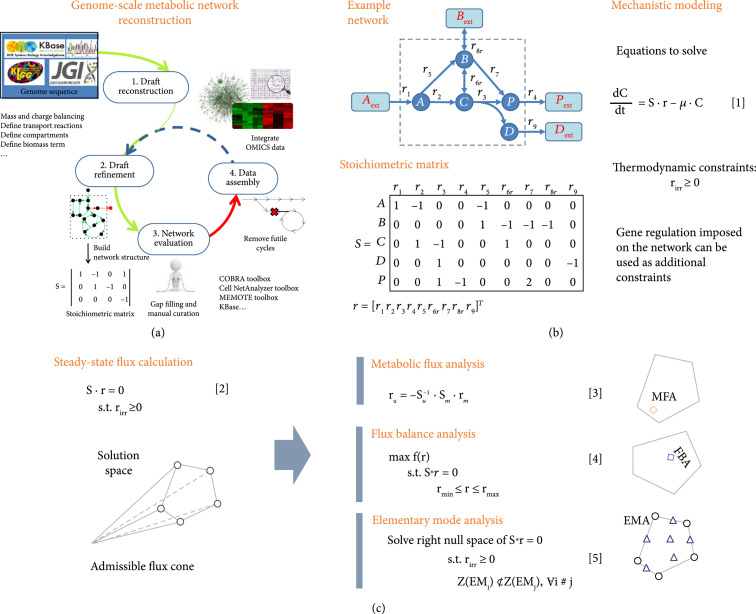
Mechanistic modeling for plant biosystems design. (a) General steps in reconstructing a genome-scale metabolic network. (b) Mass balance of metabolites in a metabolic network. Equation 1 is a system of ordinary differential equations describing dynamic chemical transformation of metabolites in a metabolic network where C is a metabolite concentration vector, S is a stoichiometric matrix, r is a reaction flux vector, and μ is the cell growth. (c) Calculation of steady-state flux distributions. Three common methods can be employed to determine metabolic flux distributions including metabolic flux analysis (MFA), flux balance analysis (FBA), and elementary mode analysis (EMA). For a typical metabolic network, a system of homogenous equations [2] is highly underdetermined, resulting in an infinite solution space. MFA determines a physiological state of a cell under a defined condition by calculating $r_{u}$ based on experimentally measured fluxes $r_{m}$ that make [2] being determined. Here, $r=\left[r_{u}\;r_{m}\right]$, and $S=\left[S_{u}\;S_{m}\right]$. FBA also determines a physiological state of a cell by implementing a cellular objective function subject to (s.t.) mass balance and flux bounds. Different from MFA and FBA, EMA unbiasedly seeks to identify all finite admissible fluxes in the solution space by imposing the thermodynamic constraints of reaction direction and pathway nondecomposability. Adapted from [30, 34, 371].

Mechanistic modeling of cellular metabolism, based on the law of mass conservation, is used to interrogate and characterize complex plant biosystems with capabilities of linking genes, enzymes, pathways, cells, tissues, and whole-plant organisms. Starting from the plant genome sequence and omics datasets, a metabolic network can be constructed based on metabolites and reactions representing nodes and edges, respectively [24] (Figure 3(a)). By defining the plant cell as a control system, the mass conservation for each metabolite can be written to decipher the fluxes of chemical elements (e.g., carbon, electron, nitrogen, and phosphate) within the plant system. These fluxes can be used as a basis to quantitatively describe cellular phenotypic characteristics [25–27]. Mathematically, mass conservation can be expressed as a system of ordinary differential equations (ODEs) to delineate the rate of change for each metabolite in the network (Figure 3(b)). The metabolic fluxes are the reaction rates determined by the metabolite concentrations, enzyme activities, enzyme concentrations, and operating conditions (e.g., temperature, pH, and ionic strength) where enzymes are encoded by genes. The first effort to create a genome-scale model (GEM) in plants was achieved for *Arabidopsis* about a decade ago [28, 29], and this has been improved and expanded since then. Today, there are 35 published GEMs for more than 10 seed plant species [30]. GEMs can be applied to plant biosystems design in the context of metabolic engineering, plant-microbe interactions, evolutionary processes, analysis of biological properties, prediction of cellular phenotypes, and model-driven discovery [31–33]. A plethora of tools can be used to analyze the plant metabolic network and enable the design of new metabolic networks. If the knowledge of reaction kinetics of the network is known, the cell phenotypes can be defined by solving the ODEs directly. Often, this approach is best suited for analyzing small reduced networks due to lack of kinetic information. Alternatively, one can interrogate the steady state solutions of the network (Figure 3(c)). Since the metabolic network is highly underdetermined, the exact phenotypes of the cell can be evaluated by performing extensive flux measurement to make the system being determined via stable isotope-labeling (e.g., ^13^C-labeled CO_2_) experiments. Alternatively, constraint-based metabolic analyses can be employed using either flux balance analysis (FBA) or elementary mode analysis (EMA) [30, 34]. FBA can predict a cellular phenotype based on an objective function (e.g., maximization of cell growth or a product synthesis), whereas EMA unbiasedly identifies all possible phenotypes for a given network. Through decades of development, tools for constructing and analyzing metabolic networks are quite mature and useful for plant biosystems design. However, several key challenges still remain: (1) the lack of knowledge of gene functions and their regulation required for accurate and comprehensive network curation and analysis [23]; (2) the lack of experimental data to decipher metabolites, reactions, and pathways that exist in compartments within a cell and among different cell types of a plant; and (3) the hidden underground metabolism due to enzyme promiscuity [22, 35]. Advances in single-cell/single-cell-type omics (see Section 3.6.2) are critically required to address these challenges.

#### 2.1.3. The Evolutionary Dynamics Theory of Plant Biosystems Design

Extant plants are the products of evolution driven by selection and random genetic drift acting on heritable phenotypic variations caused by mutations (e.g., point mutations, insertions, deletions, and gene birth/death), recombination, gene/genome duplication followed by diversification, and transgenerational epigenetic changes [36, 37]. Within an evolutionary context, several important theoretical questions remain to be answered: To what extent are the existing plant biosystems optimized for adaptation vs. production? Which plant genes and metabolites are essential, and which are spandrels: nonadaptive byproducts of evolution [38, 39]? In other words, can we simplify and perhaps improve the gene-metabolite network by removing some optional edges or nodes? Can we rewire/modify natural networks and/or introduce new components into existing networks for genetic improvement of certain traits without negative impact on other traits? The implementation of novel, orthogonal features poses a special challenge, as the interaction with the native network(s) cannot be predicted and require a strong evolutionary adaption of a system.

Plant biosystems design generates either genetically modified or *de novo* plant genomes, which will likely face evolutionary pressures caused by spontaneous mutations and natural/artificial selection. In *Arabidopsis thaliana*, the estimated haploid single nucleotide mutation rate and insertion/deletion mutation rate are $6.95\times 10^{-9}$ per site per generation and $1.30\times 10^{-9}$ per site per generation, respectively [40]. Somatic mutations have been reported in various tissues of multiple plant species, with a high proportion of mutations in shoots in perennials being transmissible [41]. In a long-lived woody perennial species *Populus trichocarpa*, the somatic mutation rate is estimated to be approximately $1.99\times 10^{-9}$ base substitutions per site per generation [42], slightly lower than that in *A. thaliana* ($7\times 10^{-9}$) [43]. Also, the plant phenotype generated from a genome could potentially exert a feed-back loop by either maintaining genome stability or guiding genome variations [44]. Therefore, a key question emerges: What new mechanisms can be implemented to maintain the stability of genetically modified genomes or *de novo* genomes?

### 2.2. Principles of Plant Biosystems Design

#### 2.2.1. Modularity of Plant Biosystems Design

Modularity is the fundamental principle of efficient and reproducible construction and maintenance of complex systems [45]. From the perspective of engineering, this has been the driving force for the modern industrialization. A module can be defined as an essential and self-contained functional unit relative to the product of which it is part, with standardized interfaces and interactions that allow composition of products by combination [46]. A modular system can be classified into sessional and chassis-based architectures. The sessional architecture has all components assimilated to modules and shares a common interface, e.g., a piping system, in which pipes are connected with a common interface for fluid transport. The chassis-based architecture can be further subclassified into the bus and slot architectures. The bus architecture (e.g., a USB port) uses the same interface whereas the slot architecture uses different interfaces, e.g., an automobile which is comprised of a chassis with many interfaces with various modules (e.g., wheels and headlights). Plant biological systems exhibit a similar principle of modularity, which has persisted for millions of years under natural selection. The principle of modular design in biological systems has been revealed at the molecular level using network theory in combination with advances in sequencing, omics, and imaging technologies over the past few decades [46–51]. Even though the principles of modular design in both biological and engineered systems are very similar, the former is much more complex, exhibiting all modular design architectures across scales (from genes to enzymes, pathways, cells, and whole organisms), and more importantly, having a unique capability to evolve (e.g., plasticity with a rewiring in response to perturbations).

**Figure 4:**
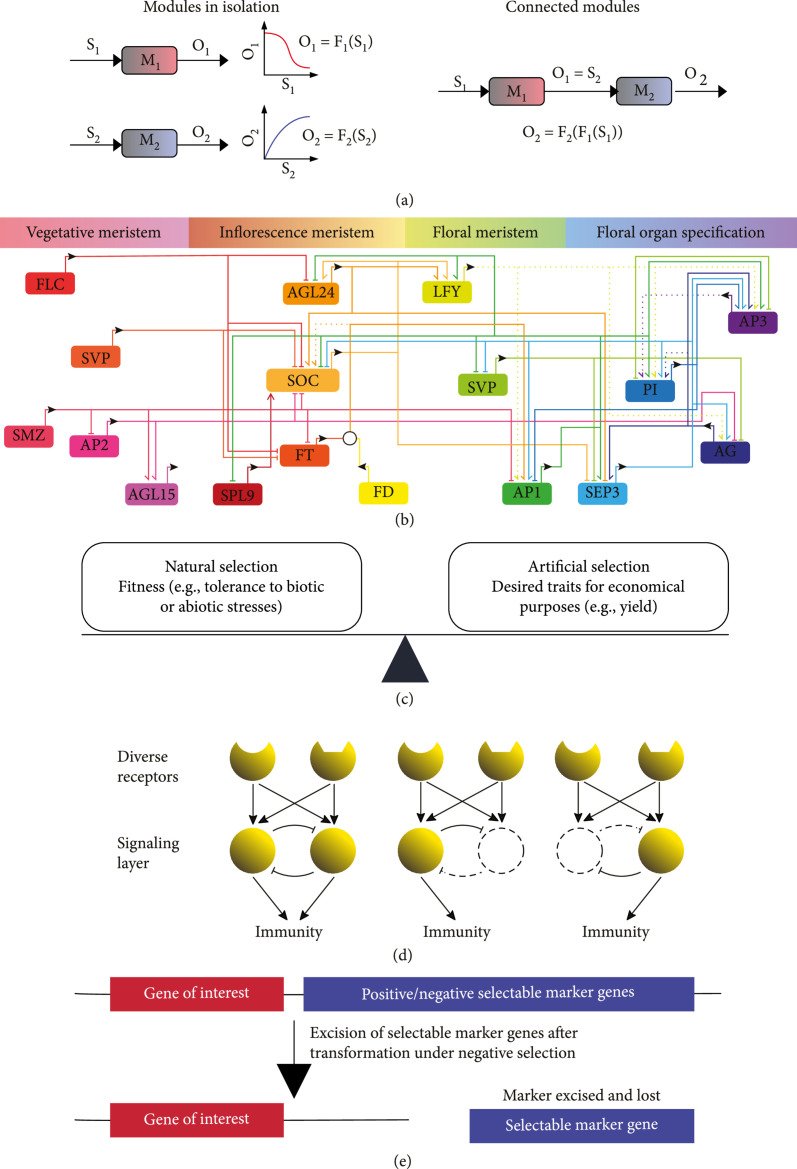
The principles of plant biosystems design. (a) Modularity; M1 and M2 represent two different modules; F1 and F2 represent the processes converting inputs S1 and S2 to outputs O1 and O2, respectively; M1 and M2 are connected, with O2=S1; redrawn from Grunberg and Del Vecchio [372]. (b) Dynamic programming as exemplified by the expression of regulators (suppressors or activators) programmed in the sequential developmental stages from a vegetative meristem to a floral meristem; redrawn from Kaufmann et al. [56]. (c) Tradeoff between natural selection and artificial selection. (d) Genetic stability, as exemplified by the pathogen-associated molecular pattern- (PAMP-) triggered immunity signaling network, with the inhibitory loops within the network to provide buffering interference (i.e., loss-of-function of some network components releases associated inhibitory loops allowing other components of the network to compensate for the loss); redrawn from Tyler [373]. (e) Upgradability, as exemplified by marker-free systems, in which the selectable marker gene can be excised from the plant genome after transformation, to allow for unlimited rounds of genetic transformation.

It is critical for plant biosystems design to fundamentally understand the principles of modular design so as to harness them for innovative applications such as challenges related to human health, food, energy, and the environment. For instance, the collective effect of genes within a module or subnetwork should be considered to achieve desirable phenotypic traits. Characterization of input/output properties of the subsystems (or modules) in isolation, and understanding how these are connected to each other, would allow inferring the behavior of complex systems by composing the behaviors of its subsystems (Figure 4(a)). For optimizing existing gene modules in plants, several alternative strategies can be explored, including (1) modifying the protein sequence or changing the gene expression of rate-limiting steps of the signaling or metabolic pathways, (2) manipulating the gene expression of master regulators that control the expression of multiple genes in the target module, (3) engineering enzymes to regulate metabolites that mediate epigenetic control of multiple genes in the target module, and (4) optimizing kinetics of metabolic reaction. These represent “homologous” approaches, whereas “heterologous” approaches, which use cell free systems or a simplified host for reconstruction of modules to evolve and/or identify essential components in the absence of endogenous interference [52, 53], would be useful for optimizing modular design. For installing new modules in plants, all the network components should be configured in gene circuits with the appropriate spatial and temporal expression patterns, with no or minimal negative side effect on the target plants.

Improved plant biosystems design requires a better understanding of the following questions: What makes up a modular (chassis) cell and exchangeable production modules and their interfaces? How can a modular cell be created as a chassis to effectively couple with exchangeable production modules to achieve desirable phenotypes? How can modular design be implemented to minimize potential tradeoff among robustness, compatibility, and evolution [51, 54]? Critical to addressing these challenges is to reconstruct an accurate plant metabolic network (see Section 2.1.2). Furthermore, recent advances in metabolic network modeling and analyses in combination with Pareto optimization theory, computational algorithms, and high performance computing will help shed light on understanding and harnessing modularity of plants at various levels such as single-cell level (e.g., stem cells and stomata) and tissue level [51, 54, 55]. Application of modular design to plant biosystems needs to consider orthogonal interactions (i.e., if a nonnative/nonnatural metabolite is introduced into the system, how can we test and/or predict if other enzymes and/or transcription factors would react with this unnatural metabolite). This would in principle require an intensive testing of cross-reactivity of metabolites with enzymes and proteins to assess side activities.

#### 2.2.2. Dynamic Programming of Plant Biosystems Design

For biosystems design, it is critical to consider the dynamic genetic programming for plant growth, development, and response to environmental perturbations. Plant biosystems design involves an ability to turn gene networks on or off in the designated tissue, time, and life cycle, while interacting with environmental input. For example, the expression patterns of regulators (suppressors or activators) are genetically programed in the sequential developmental stages from a vegetative meristem to a floral meristem [56], as illustrated in Figure 4(b). The change in gene expression during the transition from apical to floral meristem is governed by various regulators (e.g., ncRNAs, transcription factors, chromatin remodelers, and hormones) in response to environmental signals (e.g., temperature, photoperiod, and nutrient status) and endogenous cues (e.g., plant age) [57, 58]. For dynamic genetic programming in plants, it is necessary to consider not only the abundance of transcripts and proteins but also epigenetic or posttranslational modifications. For example, in *Arabidopsis*, a novel regulatory mechanism, which depends on cofactor switching mediated by phosphorylation of the photorespiratory enzyme hydroxypyruvate reductase 1, is responsible for the regulation of photorespiratory fluxes in response to the changing environmental conditions [59]. The major challenges in dynamic programming of plant biosystems are: What are sensors and regulators to enable dynamic programming? How can these dynamic regulatory systems be created and controlled, e.g., how can the turnover of mRNAs and proteins be controlled?

#### 2.2.3. Tradeoff between Natural Selection and Artificial Selection

Biosystems design for the industrial purpose of yield maximization or minimal resource utilization may not be orthologous with natural evolution, in which some natural biochemical pathways are presumably optimized for environmental fitness [60]. As most crop plants are grown in open environments, they are still at least partially under natural selection pressure while artificial selection plays an important role in plant domestication. Plant biosystems design might encounter a compromise between the natural selection for fitness and the artificial selection for agricultural and/or industrial purposes (Figure 4(c)). Alternatively, some biosystems design modifications of crops may be selected for under both natural and artificial selection. Engineered photorespiratory bypasses that increase growth rate may form such an example [61, 62]. For example, genetic improvement in yield or quality of crop plants needs to be balanced with stress tolerance enhancement. Alternatively, can these beneficial traits be coupled to ameliorate the tradeoff as part of the biosystems design to increase crop yield and quality under natural environmental inputs and fluctuations?

#### 2.2.4. Genetic Stability of Plant Biosystems Design

As plant genomes are prone to spontaneous mutations (see Section 2.1.3), the capability to maintain the genetic stability of plant biosystems design over a long period of time (e.g., many generations of annual plants and life span of perennial plants) is critical. Also, epigenetic changes may have an impact on the stability of plant biosystems design. Robust traits in multiagent complex systems can be generated through networked buffering mechanism, which features a concurrent, distributed response involving chains of agents with versatility (i.e., agents perform more than one single functional role) and degeneracy (i.e., there exists partial overlap in the functional capabilities of agents) [63]. For example, the pathogen-associated molecular pattern- (PAMP-) triggered immunity signaling network is highly buffered against interference, with the inhibitory loops within the network providing buffering (i.e., loss-of-function of some network components releases associated inhibitory loops to allow other components of the network to compensate for the loss) (Figure 4(d)). Plant biosystems design using long-term buffering strategies such as network buffering or using proteins with multiple functions may produce more robust traits (e.g., disease resistance) that would last for a long period of time (e.g., many generations, tens or hundreds of years).

#### 2.2.5. Upgradability of Plant Biosystems Design

In general, it is important to design a product that can adapt to future required performance and functions via upgrading the components of a biosystem [64]. Since plant biosystems design may require multiple iterations of Design-Build-Test-Learn (DBTL) cycles (for details see Section 3), it is essential that the genetically modified plants or *de novo* plant systems can be easily upgraded for improving performance or adding new functions. In general, upgrading the plant genome requires consecutive stable plant transformation processes, which is constrained by a limited number of selectable marker genes available for plant transformation, including widely used selectable marker genes conferring antibiotic (e.g., kanamycin and hygromycin) or herbicide (e.g., BASTA) resistance [65], along with some nonantibiotic and nonherbicide markers such as plant phosphomannose isomerase [66], broad-specificity amino acid racemase [67], and fluorescent proteins [68, 69]. For enabling upgradability of plant biosystems design, it would be desirable to consider marker-free plant transformation systems, in which the selectable marker gene can be excised from the plant genome after transformation (Figure 4(e)). Selectable marker genes can be self-excised in plants using various approaches mediated by site-specific recombinase [70–73], zinc finger nuclease [74], and CRISPR [75, 76].

## 3. Technical Approaches for Plant Biosystems Design

**Figure 5:**
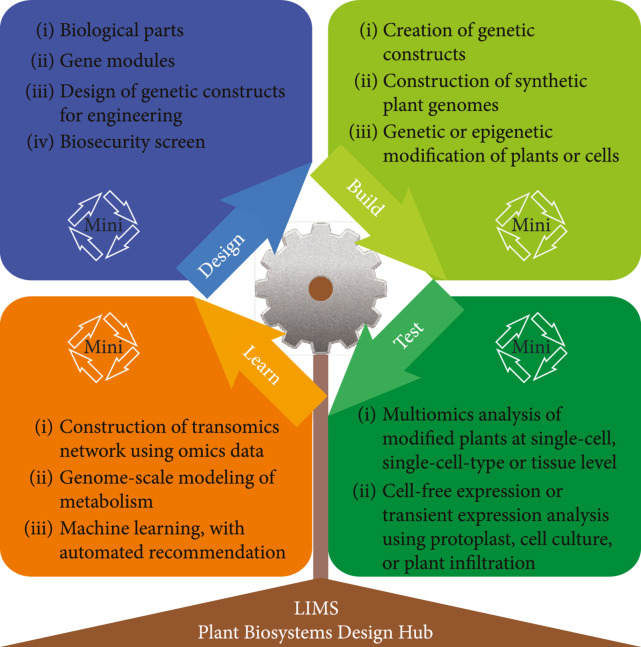
Technical approaches for plant biosystems design. In general, plant biosystems design requires iterative cycles of design-build-test-learn. The number of cycles varies with the plant traits to be engineered. LIMS: laboratory information management system.

In general, a plant biosystems design approach goes through iterative DBTL cycles. This approach has been widely practiced in the biosystems design of microbial systems [77, 78], but its application to plant biosystems design is still limited, mainly due to much longer time needed to complete DBTL cycles in plants. It would be important to see how recent attempts of accelerating DBTL using cell-free protein synthesis (CFPS) systems [53, 79] would impact cellular approaches in plants. On the other hand, at the organismal level, there is a need for establishing state-of-the-art capabilities for plant biosystems design, including modular cell design, validated biological parts, automated design and build of genetic constructs, generation and testing of plant genetically modified or *de novo* plant systems, and learning from the test data, and integrating “design (D),” “build (B),” “test (T),” and “learn (L)” as well as executing mini-DBTLs (Figure 5). For effective execution of the DBTL cycles, a laboratory information management system (LIMS) could be used to facilitate local data acquisition and sharing. Also, a Plant Biodesign Hub (PBH) needs to be established as an open access online platform for biological parts registration, genetic circuit design, and predictive modeling based on test data. Although other repositories are already in use and have proven effective, they cannot meet the increasing needs of the growing biosystems design community in terms of data comparability as well as the integration of data curation, submission, biological knowledge, and circuit design.

### 3.1. Mini-DBTL and Integration

Each component of the Shewhart cycle (D, design; B, build; T, test and L, learn) has their steps for control and continuous improvement forming a DBTL cycle within each D, B, T, and L, named here mini-DBTL. For example, a mini-DBTL within the "D" step could represent formulated DNA sequences that have failed synthetic DNA fragments synthesis and thus have their nucleotide sequence studied, redesigned, resubmitted for synthesis, and reanalyzed to conjunctively inform core adjustments necessary (e.g., maximum local GC content, repeats, homopolymers, and hairpins) for future attempts to succeed [80]. Similarly, researchers may need to (1) attempt different approaches before completing the assemblies (e.g., in the "B" step), (2) evaluate process variation impacting data acquisition (e.g., in the "T" step) [81], (3) develop tools that improve predictive power while making dissimilar suggestions (e.g., in the "L" step) [82], and (4) iterate automation [83]. Such improvements, along with the use of LIMS, robotics, and physical/electronic repositories (e.g., ICE, EDD) [84, 85], will need to be accounted for accelerated and reproducible biosystems design.

### 3.2. Modular Cell Design

Network modeling has mainly been employed to elucidate complex phenotypes of existing plant biosystems. In principle, the approach can be applied to design plant systems *de novo*. A recent computational advancement in network modeling using Pareto optimization theory has been described in the ModCell algorithm, which has enabled rational design of a modular (chassis) cell that can be coupled with many exchangeable production modules to achieve various desirable production phenotypes in bacteria [45, 51, 54, 55, 86, 87]. This modular cell engineering approach is aimed at generating production strains rapidly with efficient performance while minimizing the number and cycle time of DBTL cycles. While the ModCell tool may prove very useful to guide the parts, modules, and chassis selection for prokaryotic cells, the compartmentalized endomembrane architecture of eukaryotic cells and the interconnected multicellular nature of complex plant systems featuring specialized cell types remain a considerable challenge for plant biosystems design.

### 3.3. Curation of Validated Biological Parts

Libraries of validated parts (e.g., protein coding sequences, regulatory elements for gene expression, signaling, and other functional genetic elements) are critical for the engineering of multicomponent biological systems quickly and reliably [88, 89]. Several repositories have been established, such as the iGEM Registry of Standard Biological Parts (http://parts.igem.org) [90, 91], SynBioHub (https://synbiohub.org) [92], and the Addgene repository (https://www.addgene.org) [93, 94]. While these repositories are overwhelmingly dominated by biological parts of prokaryotic origin, they host some DNA parts useful for plant biosystems design, such as the MoClo Toolkit [95, 96] deposited in Addgene. Recently, a library of chloroplast-specific parts was established for plant biosystems design using the plant chloroplast as a chassis [97].

Given the increased complexity of plant genes over those of prokaryotes due to the presence of introns, distal regulatory elements, and posttranscriptional processing signals, a common “Phytobrick” syntax has been developed to enable universal Type IIS assembly with standardized parts (see Section 3.5.1) [89]. Despite these advancements, the conversion of natural DNA sequences into Phytobricks can be laborious, and the removal of “illegal” restriction sites in these sequences to enable Type IIS assembly can introduce unintended alterations to the part’s function. In the recent production of a standardized parts library of 221 *Eucalyptus* transcription factors and 65 promoters [98], the risk of altering promoter function in their conversion to Phytobricks was minimized by using known single nucleotide polymorphism data in *Eucalyptus* populations to mutate undesirable restriction sites.

The biological parts for plant biosystems design have been obtained mainly from natural sources (i.e., plant genomes). Recently, a library of synthetic transcriptional regulator systems, which include synthetic activators, synthetic repressors, and synthetic promoters, was established to control plant gene expression in a tissue-specific and environmentally responsive manner [99]. Genome recoding (i.e., rewriting codon meaning through chemical synthesis for new features) has been practiced in microbial systems [100] and could be used to generate novel biological parts for plant biosystems design.

**Figure 6:**
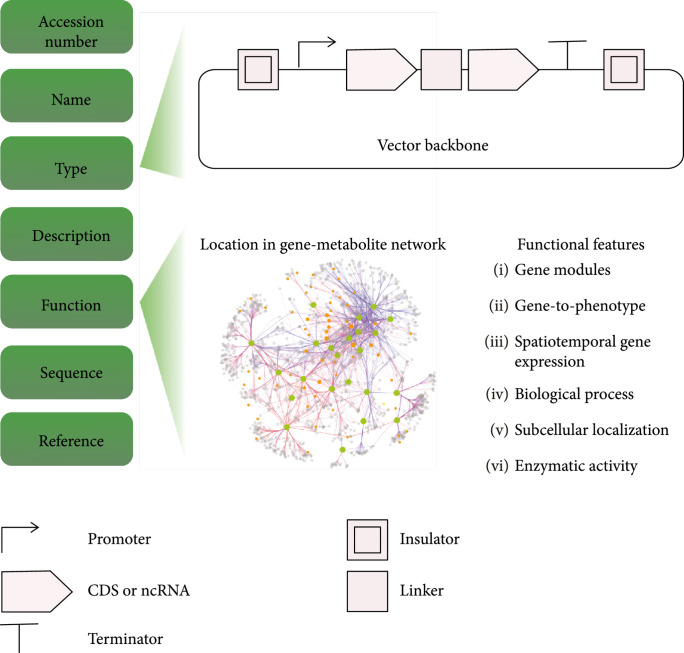
A biological parts registration and curation module for plant biosystems design. The registration form includes accession number, name, type, description, function, sequence (i.e., DNA and protein sequence), and references (e.g., publications associated with the biological parts). For illustration purpose, the functional items are listed for protein-encoding genes only. CDS: protein-encoding sequence; ncRNA: noncoding RNA sequence, including natural noncoding RNAs and guide RNAs for genome editing.

To facilitate international access and reproducibility of data, a centralized knowledgebase of validated biological parts needs to be established, which includes specific experimental context, standardization, and crossed references among different databases (e.g., iGEM, SynBioHub, and Addgene) [85]. The nomenclature of such a knowledgebase would build upon structures already defined by the parts repositories listed above and expand the concept from genomic constructs such as promoters and coding sequences into plant specific structures. Such an initiative could be established through collaboration with KBase [101], with the capability of submission, query, functional mapping onto the gene-metabolite network, as illustrated in Figure 6. There are several challenges to realize the standardization of biological parts across the international plant biodesign research community. For example, (1) how can data be standardized and made comparable between different laboratories? And (2) how can part characterization be rewarded? These challenges may be addressed through international workshops sponsored by a professional society, with participants from academia and industries in the future. For registration of biological parts, it would be important to include negative results, which typically do not get published or reported, to avoid wasteful repetitions in different labs. The negative results would also be very helpful for computational design of synthetic biological parts using machine learning approaches.

### 3.4. Genetic Construct Design

**Figure 7:**
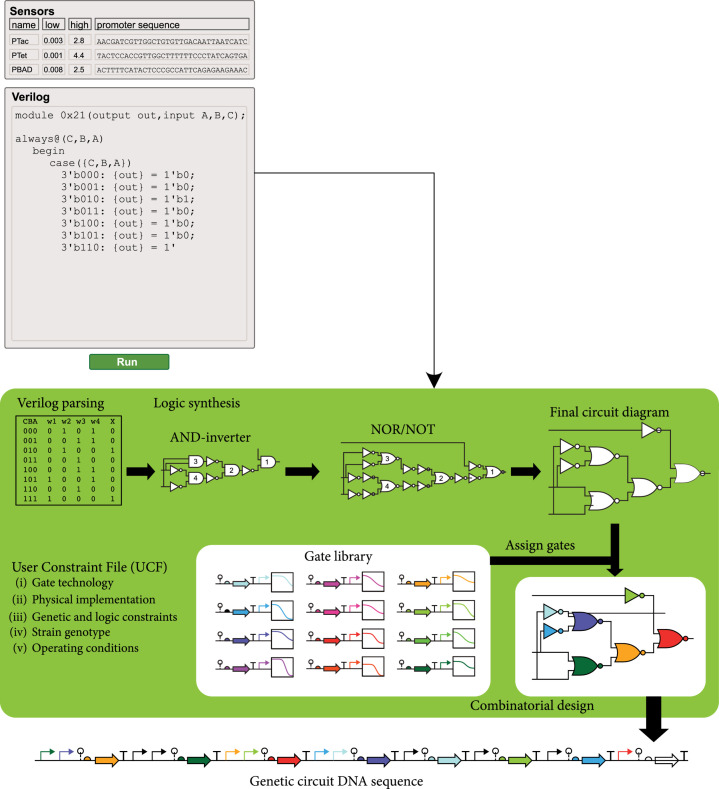
An automated gene construct design module for plant biosystems design. Redrawn from Nielsen et al. [115].

Genetic constructs are designed for nucleic acid sequence modification, gene expression regulation, and metabolic engineering. The genetic design for genomic sequence modification *in vivo* can be achieved through different methods, including CRISPR/Cas-mediated genome editing, with an emphasis on maximizing on-target efficiency and minimizing off-target effects [102]. Recently, rapid progress has been made in the development of new genome editing technologies, such as high precision prime editing [103], cytosine base editors [104], and adenine base editors [105]. These new technologies have been tested and adapted for genome editing in plants [106–110]. The design for modulating gene expression can be achieved through CRISPR interference (CRISPRi) and activation (CRISPRa) systems, in which nuclease-deactivated Cas9 (dCas9) is tethered to inhibitory and activating domains, respectively, to regulate gene expression [111, 112]. CRISPRi may also be achieved with dCas9 alone acting on promoter or exonic sequences. In addition, RNA editing allows altering splicing or introducing nonheritable changes to protein sequences [113]. It is also possible to multiplex activation, repression, sensing, and emulation of gene expression using homologous CRISPR-sgRNA pairs or different CRISPR-associated RNA scaffolds, which can be potentially used to build complex synthetic programs [102, 114]. For designing predictable gene circuits, genetic design automation (GDA) tools compatible with Synthetic Biology Open Language (SBOL), such as Cello [115] and SBOLDesigner 2 [116], could be adapted to plants and used in an integrated fashion with the knowledgebase of biological parts to streamline the design process, as illustrated in Figure 7.

### 3.5. Building Genetic Constructs and Synthetic Plant Genomes

#### 3.5.1. Assembly of DNA Parts into Genetic Constructs

**Figure 8:**
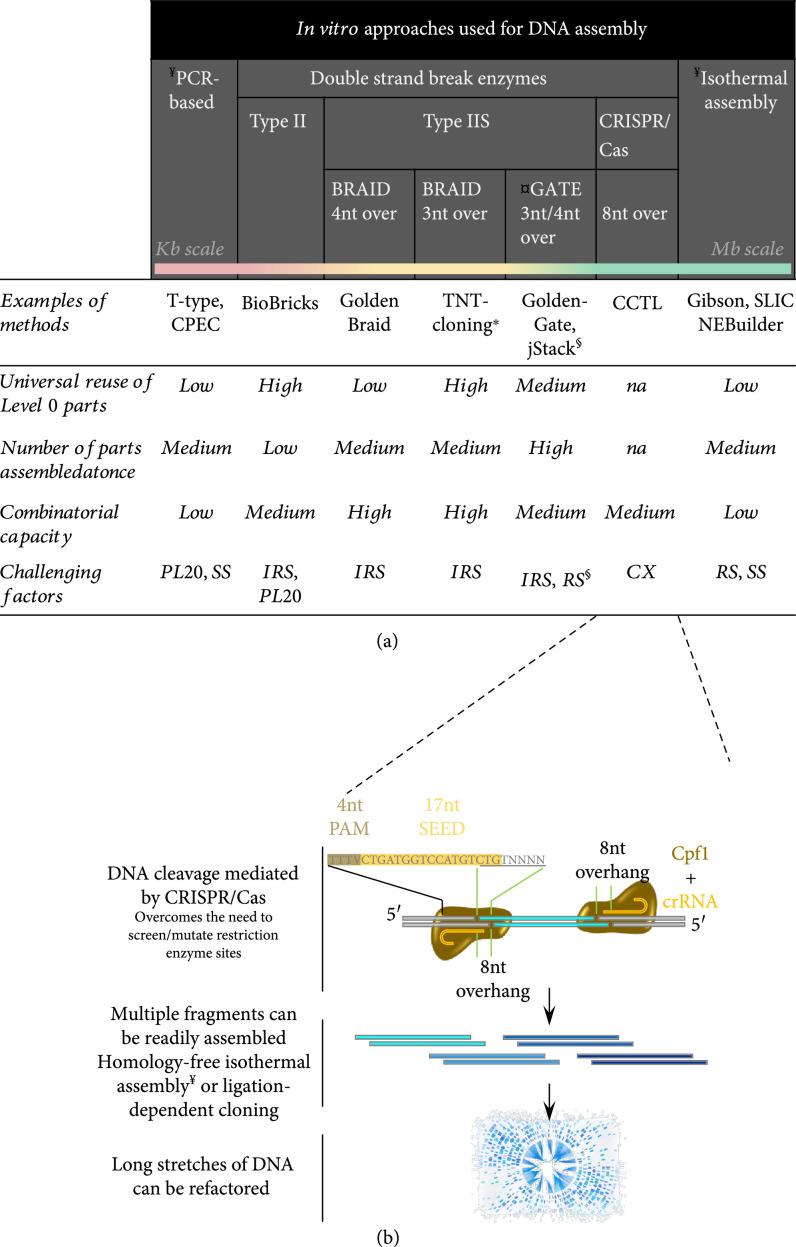
Strategies for DNA assembly across scales relevant for plant biosystems design. (a) Major platforms and key factors considered for executing large DNA assemblies across scales. (b) Detail of the CCTL method. crRNA sequence is from [123]; overhang is underlined with the last 4 nt being programmable [124]. ^¥^Produce scarless assemblies (NEBuilder allows ssDNA oligos in substitution of homologous overlap).^¤^May leave scars. ${}^{*}$Undesired type IIS restriction sites can be partially masked by oligos [122]. For BRAID systems, see [120]. ^§^Hybrid system using golden-gate followed by *in vivo* homology-based assembly [129]. CX: complex design and execution; PL20: parts size/length beyond 20 kb; RS: repetitive sequences; SS: secondary structures; IRS: internal restriction sites. Recombination-based approaches (e.g., Gateway) were omitted due to limited use for biosystems design.

Genetic constructs are built using various DNA assembly methods which can be based solely on PCR reactions (e.g., T-type), sequence-dependent recombinases (e.g., Gateway), enzymes causing specific DNA double strand breaks (e.g., types II and IIS), or an enzyme mix that coordinate nucleotide polymerization (e.g., Gibson assembly) [117–122] (Figure 8(a)). Each of these methods features a unique set of characteristics, and their combinatorial (i.e., shuffling) capacity, hierarchical (i.e., stacking) support, strengths dealing with secondary structure and repetitive sequences will guide their appropriation to different tasks for plant biosystems design. A major challenge in the field has been the editing of specific parts within a large preassembled DNA fragment, which can be addressed by the CCTL method (Cpf1-assisted cutting and Taq DNA ligase-assisted ligation) [123–125] (Figure 8(b)).

Type IIS restriction endonuclease-based DNA assembly systems are widely used for hierarchical assembly of DNA fragments into genetic constructs, such as GoldenBraid [120], TNT-cloning [122], and universal Loop assembly (uLoop) [126], which are based on Golden Gate [118]. These approaches have two advantages: (1) the DNA parts can be individually cloned into the entry vectors to establish biological parts libraries, which can be shared in the scientific community, and (2) multiple rounds of binary to hexanary assemblies can be performed to join various numbers of DNA fragments in flexible configurations. However, all type IIS-based approaches suffer from prohibitive internal restriction sites. These sites can be partially masked [122] but not eliminated, and innovative approaches mutating deoxy-adenine within such prohibitive sites to unnatural nucleotides (e.g., deoxy-NaM) during PCR could advance these methods due to *E*. *coli*'s natural ability to restore the original adenine-rich restriction site *in vivo* [127].

Multiple DNA fragments with unique short (e.g.,15-20 bp) overlaps between neighboring parts can be assembled using isothermal assembly such as Gibson Assembly, in which a 5${}^{\prime }$ exonuclease removes nucleotides from the 5${}^{\prime }$ ends of double-stranded DNA molecules, complementary single-stranded DNA overhangs are annealed, a DNA polymerase fills the gaps, and a DNA ligase seals the nicks [119]. Recently, another similar approach, called “SureVector,” was developed to assemble multiple DNA fragments with 30 bp overlapping ends, in which DNA parts are denatured and adjacent parts are annealed due to the overlaps followed by DNA polymerase-mediated partial extension of exposed 3${}^{\prime }$-OH ends, resulting in flaps that are digested by an endonuclease and covalently joined by a ligase [128]. These approaches have several key advantages: (1) allowing for assembling multiple blunt-end DNA fragments in a single-tube reaction and (2) no reliance on restriction enzyme digestion of DNA fragments and consequently no requirement for removing or mutating type IIS restriction sites. On the other hand, these methods have a reduced capacity to assemble multiple parts at once (compared to Golden Gate) and have their efficiency strongly impacted by repetitive sequences as well as sequences prone to secondary structure when single stranded. Under such circumstance, using hybrid methods, which combine the advantages of two or more approaches at different levels while being amenable to the drawbacks, will be highly beneficial [129].

#### 3.5.2. Plant Synthetic Genomes

Recently, a 785 kb *Caulobacter ethensis*-2.0 (*C. eth*-2.0) genome was constructed in yeast using multiple rounds of homologous gap repair approach [130]. A 4 Mb synthetic *Escherichia coli* genome was constructed through a high-fidelity convergent total synthesis [131]. However, the construction of plant synthetic chromosomes through DNA synthesis has not been reported yet. A synthetic plant chromosome vector requires a minimum of centromeric and telomeric sequences, origins of replication, and a selectable marker gene [132]. A notable step towards the generation of synthetic plant genomes was the full cloning and yeast-mediated modification of the 204 kb plastid genome of the algae *Chlamydomonas reinhardtii* [133]. Plastids are among the defining features of plants, and their relatively small and well conserved genomes, along with the potential for high-level expression of desired genes, are currently more tractable candidates for total synthesis than plant nuclear genomes [134].

### 3.6. Testing Genetic Constructs in Plants

Testing of plant biosystems designs is mainly achieved through stable transformation and transient expression (e.g., agroinfiltration and protoplast transformation) of genetic constructs, sometimes followed by omics (epigenomics, transcriptomics, metabolomics, proteomics, and phenomics) analysis of genetically modified plants or *de novo* plant systems. Stable transformation is a bottleneck of the DBTL cycle because the main limitation remains to generate many transgenic plants transformed with multigene constructs. What can be improved at a throughput level using protoplasts is at the expense of an understanding of the construct design’s effect on whole-plant performance and fitness, and likewise, protoplast-based systems will not be suitable for all traits being engineered.

#### 3.6.1. Stable Transformation and Transient Expression of Genetic Constructs

*Agrobacterium*-mediated transformation has been the major approach for plant genetic engineering. However, there is substantial variation in the amenability to *Agrobacterium*-mediated transformation among plant species and even cultivars of the same species, with high-efficiency transformation protocols available for a limited number of plant species/cultivars. One limitation for using *Agrobacterium* to transform plants is that not all plant species are *Agrobacterium*-infectable. Another major bottleneck in *Agrobacterium*-mediated transformation is *in vitro* regeneration of shoots or embryos from transformed cells. Ectopic expression of morphogenic or developmental regulator genes (e.g., *Baby boom* and *Wuschel2*) can promote somatic cells to form embryos, which develop into whole plants, in monocot species and consequently improve *Agrobacterium*-mediated transformation efficiencies dramatically [135]. Plant transformation often requires tissue culture by exposure of cells to various hormones, which are inefficient and time-consuming. Recently, a *de novo* induction of meristem approach based on the use of development regulators was developed in *Nicotiana benthamiana*, tomato, potato, and grape, avoiding the use of traditional tissue culture [136]. The generation of transgenic roots (hairy roots) through *Agrobacterium rhizogenes*-mediated transformation, leading to the production of composite plants (i.e., transgenic roots on wild-type shoots), has proven to be a fast and versatile system particularly suited for certain woody plants recalcitrant to transformation such as *Eucalyptus* [137, 138]. There is an urgent need to extend these methods to other plant species or to develop new capabilities for enabling or improving transformation in a wider range of dicot and monocot species, particularly for the ones that are currently recalcitrant to genetic transformation. *In planta* transformation methods can be particularly useful because no *in vitro* regeneration of shoots or embryos is required. It is preferred that morphogenic regulator genes are not used or that the morphogenic regulator genes could be excised out of the genome by inducible recombinase excision system, because expression of these transgenes can affect plant growth and development [139–141].

Besides *Agrobacterium*-mediated transformation, DNA, RNA, or protein molecules can be directly delivered to target sites via particle bombardment, nanoparticles, or direct injection. For example, Cas9–gRNA ribonucleases (RNPs) were directly injected into plant zygotes for DNA- and selectable-marker-free genome editing in rice [142]. Furthermore, carbon nanotubes were recently used for efficient plasmid DNA delivery into multiple plant species (e.g., arugula, wheat, and cotton) to enable high protein expression levels without transgene integration [143], which has great potential for application to transgene-free genome-modification [144] and may also be useful for functional testing of plant biosystems design.

Multigene transformation is important for plant biosystems design. It can be achieved using binary vectors based on transformation-competent artificial chromosomes, such as pHUGE-Red which is suited for cloning large DNA fragments [145]. Alternatively, multiple binary vectors with compatible replication origins can be hosted in a single *Agrobacterium* cell for simultaneous delivery of multiple gene constructs into plants [146]. Furthermore, gene stacking based on site-specific recombination and nuclease activity has a potential for *in planta* stacking of a large number of genes at a single genomic site of the same plant [147]. Also, multiple genes could be stacked using plastid transformation with operon-like and polyprotein expression systems [148–150]. CRISPR/Cas9-mediated targeted T-DNA integration and precise knock-in [102, 151, 152] can potentially be used for *in planta* stacking.

As an alternative to stable transformation approaches, which are often time-consuming, transient expression techniques via virus-induced gene silencing (VIGS) enable rapid knockdown of a targeted gene in a high-throughput manner, even in plant species that are difficult to transform [153]. However, RNA-directed transcriptional gene silencing cannot induce heritable changes that target the coding region, although targeting of the promoter sequence can cause heritable changes in gene expression mediated by methylation [154]. In combination with CRISPR/Cas systems, viruses can be used to quickly induce heritable changes in plant genomes. Recently, viruses have been used to deliver the guide RNAs or the entire CRISPR–Cas9 cassette for Cas9-mediated gene editing in plants, providing another high-throughput method for testing the function of gene constructs for plant biosystems design [155–157]. Also, multiplexed heritable gene editing was recently achieved through virus-mediated *in planta* delivery of mobile single guide RNAs (sgRNAs) into *Nicotiana benthamiana* transgenic plants expressing Cas9 [158], which provides another approach to produce heritable gene editing without tissue culture. Alternatively, a recently developed nanotube-based platform for RNA delivery, which enables stable siRNA delivery and efficient silencing of target genes in intact plant cells [159], might be useful for a broad range of applications including direct delivery of sgRNAs and Cas9 mRNA for DNA-free genome editing.

#### 3.6.2. Omics Analysis of Genetically Modified Plants

Integrative multiomics (e.g., transcriptomics, metabolomics, proteomics, epigenomics, and phenomics) analysis of genetically modified plants could provide rich experimental data for linking genetic design to plant phenotype. So far, plant omics data have been collected at the organ or tissue level, resulting in the molecular, metabolic, and biochemical information averaged over a population of heterogeneous cells [160, 161]. Because plant cellular processes vary spatially, single-cell multiomics is necessary for simultaneous analysis of different biomolecules to achieve accurate assessment of nodes and edges in the gene/metabolite networks operating in an individual cell [162]. Single-cell technologies have evolved intensively in the last decade [163], and transcriptomics and proteomics are viable at the single-cell level [164, 165]. However, most plant species still face major technical hurdles that make it challenging to achieve single-cell resolution, in large part because it is challenging to dissociate cells from the plant tissues [166] and/or collect adequate amounts of the desired biomass when no amplification strategies are available [167, 168]. Progress has been made to solve these challenges. For example, the protoplasts of *Arabidopsis* root cells have been successfully used for single-cell RNA sequencing using droplet-based microfluidics platform [164, 169–171], and laser capture microdissection has been used to isolate individual cell layers of tomato roots for single-cell-type proteomics [172]. Also, live single-cell mass spectrometry has been used for direct analysis of metabolites in a single live plant cell [173].

### 3.7. Learning from the Testing of Designed Plant Systems

**Figure 9:**
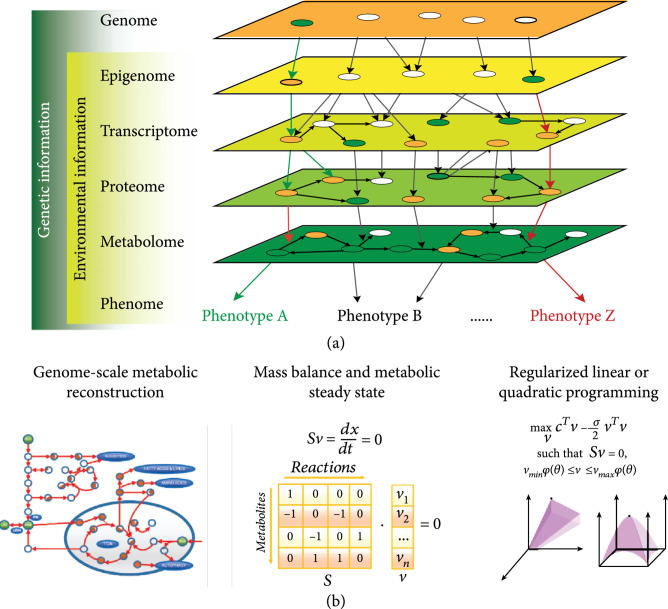
A computational learning module for plant biosystems design. (a) Integrative analysis of multiomics data; redrawn from Yugi et al. [374]. (b) Metabolic modeling; redrawn from Zampieri et al. [375].

Integration of multiomics data can provide a multiperspective view of dynamic molecular behavior and interacting networks of genes occurring in plants [174], as demonstrated in an integrative analysis of transcriptomic, proteomic, fluxomic, and phenomic data in relation to lignin biosynthesis in *Populus* [175]. The omics datasets can be integrated using statistical or advanced machine learning approaches [176] to generate results related to simple pathways or complex networks at cell, tissue, organ, or organism levels, providing insights into the effect of biosystems design on plant phenotype. For example, a machine learning method called Multi-view Factorization AutoEncoder, which uses a deep representation learning approach to simultaneously learn feature and patient embeddings, was recently developed for seamless integration of multiomics data and biological domain knowledge such as molecular interaction networks in humans [177]. This method can be extended to omics data analysis of genetically modified plants or *de novo* plant systems for improving the design of plant systems in an iterative application to the DBTL cycle, as illustrated in Figure 5. Complementary tools that use probabilistic modeling techniques to converge to the desired specification (e.g., increased expression of a gene cluster) accurately, without requiring full mechanistic understanding of the biological system [82], will need to be recruited to plant biosystems design. An exemplary module for integrative analysis of multiomics data in the “Plant Biodesign Hub” is presented in Figure 9(a). The results from the integrative analysis of multiomics data could be used for metabolic modeling based on the modeling module to be built in the “Plant Biodesign Hub” (Figure 9(b)). Besides learning from engineered plants, it would be important to learn from model species or from cross-species models incorporating prior knowledge through explainable artificial intelligence [178].

## 4. Applications of Plant Biosystems Design

**Figure 10:**
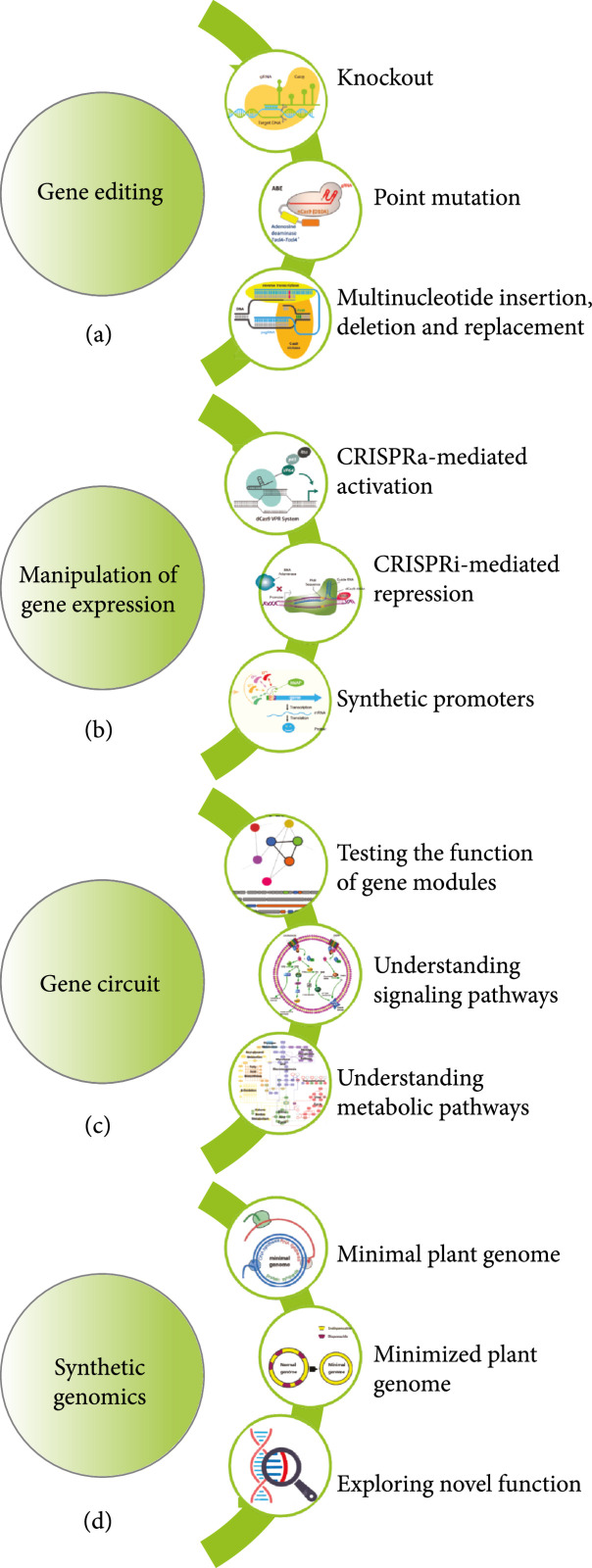
Applications of biosystems design to basic plant biology research. (a) Elucidating plant gene function through genomic mutations created by using genome editing technologies. (b) Manipulation of gene expression using CRISPR interference (CRISPRi) and activation (CRISPRa) and synthetic promoters. (c) Studying the function of gene modules, signaling, or metabolic pathways using synthetic genetic circuits. (d) Understanding the complexity of plant systems using minimal or minimized plant genomes as well as exploring novel function using synthetic genomics.

**Figure 11:**
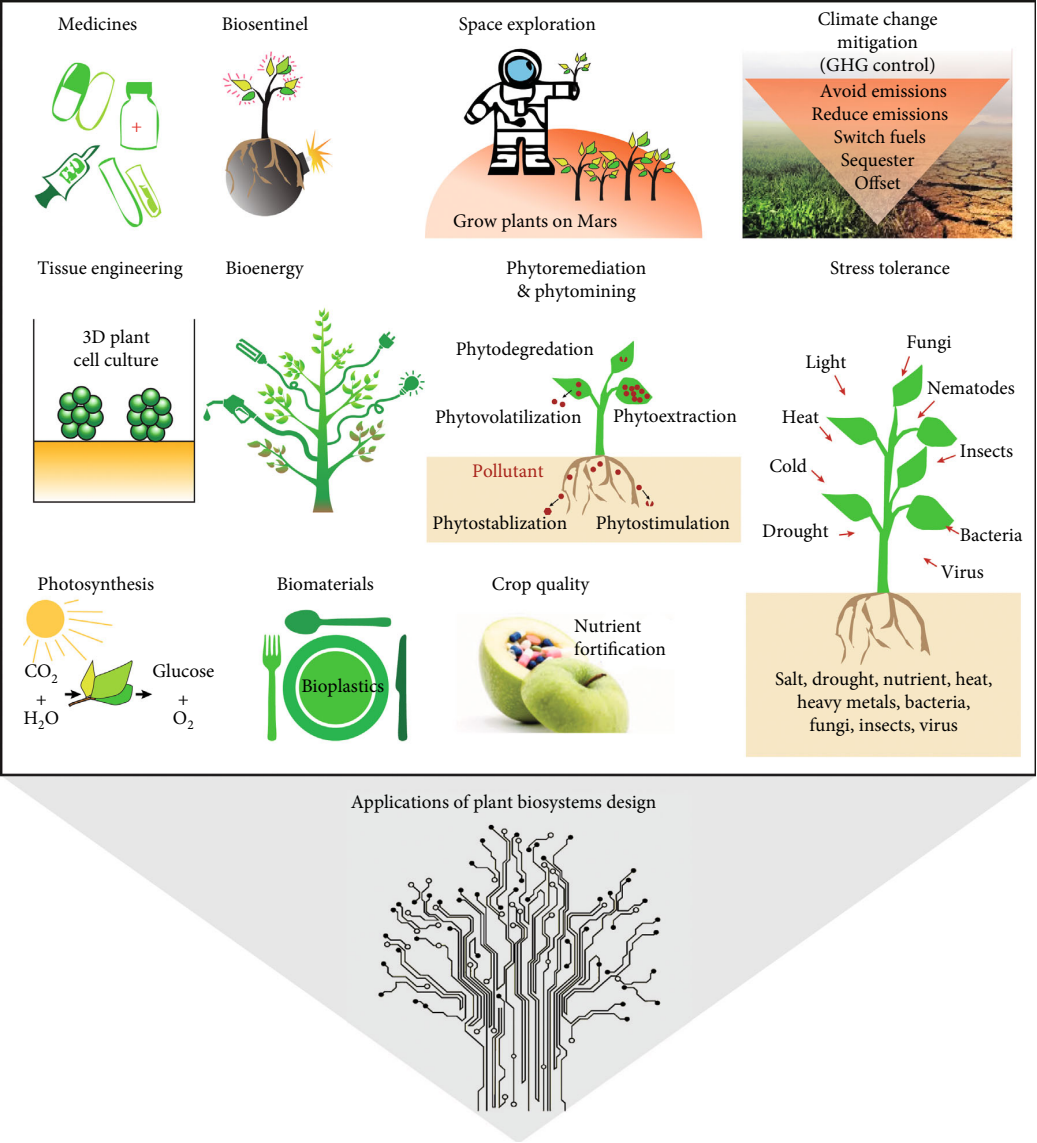
Applications of biosystems design to applied plant science research. GHG: greenhouse gas. Only representative examples are shown.

Biosystems design has great potential for applications in (1) basic plant biology research to gain a deeper understanding of molecular functions and biological processes in plant biosystems (Figure 10) and (2) various aspects of applied plant science research to accelerate the improvement of plant traits or to create new germplasms with improved traits to benefit ecosystem health and human society (Figure 11).

### 4.1. Application of Biosystems Design to Basic Plant Science Research

Biosystems design can be used to further our understanding of molecular mechanisms driving biological processes in plant systems by dissecting the function of individual genes or multigene modules.

#### 4.1.1. Understand Plant Gene Function Using Biosystems Design

Approximately 600 plant species genomes have been sequenced [179], with an increasing number of new plant genome sequences being released every year. However, even in *Arabidopsis thaliana*, which is one of the best studied model plant species, approximately 60% of predicted enzyme- and transporter-encoding genes do not have credible functional annotations, and this number is even higher in nonmodel plant species [180]. Until recently, only ~5% of genes in the *Arabidopsis* genome have experimental evidence for their functions (e.g., biochemical activity, subcellular location, and biological role) [181]. Traditionally, experimental characterization of plant gene function depends mainly on (1) knockout mediated by T-DNA insertion or chemical/radiation-induced mutagenesis, (2) knockdown mediated by RNA interference (RNAi) or VIGS, and (3) overexpression of one or a few genes in individual genetically modified plant lines. These traditional approaches suffer from several limitations: (1) the knockout mutations created by T-DNA insertion or chemical/radiation-induction occur as random insertions or deletions and often accompanied by additional unrelated mutations in the genome, (2) it is challenging to obtain homozygous multigene knockout mutants in diploids or single-gene mutants in polyploid species, as it requires multiple generations of self-pollinated plants while being almost impossible in vegetatively propagated plants and perennials with long life cycles, and (3) RNAi works on protein-coding genes only, along with incomplete loss of function and extensive off-target activities [182]. These limitations can be overcome by genome-engineering tools such as CRISPR/Cas-systems, which can be used to generate targeted homozygous knockout mutations without the need for self-fertilization [102, 182]. CRISPR/Cas-mediated gene knockout has one disadvantage for functional genomics research: it is not suitable for studying the function of essential genes due to the lethality of their knockout mutants generated by CRISPR/Cas systems, although it is possible to identify essential genes using CRISPR/Cas targeted mutagenesis in some cases where homozygous knockout mutant seeds can be obtained from heterozygous mutant parents [183]. Still, CRISPRi and CRISPRa offer the opportunities to repress and activate gene expression, respectively, for both coding and noncoding RNAs [112]. However, being similar to the traditional genetic transformation system, the CRISPR/Cas systems have not been established in many plant species. In some cases, low editing efficiency and off-target issues still cannot be fully addressed. Beyond examining the functional roles of a particular gene, more elaborate biosystems design strategies offer a powerful approach for studying the collective function of multigene modules in metabolic pathways, signal transduction cascades, and regulatory networks [184]. It is very challenging to map the protein-DNA interactions in gene regulatory networks using experimental approaches. Biosystems design could enable scalable epitope tagging for high-throughput chromatin immunoprecipitation followed by sequencing (ChIP-Seq) [185], combined with CRISPR/Cas-mediated knockout experiment, to identify accurately the target genes of plant transcriptional factors.

In general, plant functional genomics research is still substantially hindered by labor intensive and time-consuming work and therefore could greatly benefit from biosystems design approaches that provide high-throughput capabilities for determining gene function. For example, multiplex genome editing can generate more than 100 targeting events [186], enabling gain-of-function or loss-of-function screening of a large number of genes. Also, the automated design and high-throughput assembly of gene constructs described in Section 3 would greatly speed up the elucidation of plant gene functions.

#### 4.1.2. Understand the Complexity of Plant Systems Using Biosystems Design

Construction of synthetic genomes (e.g., synthetic minimal genome, massively recoded genome, chimeric genome, and synthetic genome with expanded genetic alphabet) can generate new insights into the basic principles of life and enable valuable applications [187]. Minimal genomes, which are reduced genomes containing only genes essential for life, have been constructed for multiple single-cellular organisms, such as *Mycoplasma* and *Saccharomyces* [188–190]. Recent technical advances in DNA synthesis and synthetic genomics may soon allow for the construction of minimal genomes for model plants. A minimal plant genome should enable a complete set of essential features of plants, including plant growth, development, and response to environment. A bottom-up approach based on *de novo* DNA synthesis could be used to reconstruct minimal plant genomes, but it would require information about the minimal required set of genes for plant viability. Alternatively, minimized genomes can be obtained by deletion of cryptic genes and mobile DNAs in microbes [191]. It is anticipated that creating minimized genomes for plants would be much more challenging than for microbes due to much larger and complex genomes in plants. From a biosystems design perspective, a minimized plant genome could be potentially obtained through reduction via genome-wide gene-knockout using CRISPR/Cas systems. The minimized plant genomes would provide a unique opportunity for dissecting the minimal gene network of a functional plant system and validating modular cell design. Furthermore, it would allow for adding genes or gene modules to study their function.

### 4.2. Application of Biosystems Design to Applied Plant Science Research

With guidance from the principle of biosystems design, the cutting-edge genome editing and genome-writing/rewriting technologies can be used for modifying or redesigning crop plants for various applications, including genetic improvement of photosynthetic efficiency, plant stress tolerance, crop quality, climate change mitigation, production of biomaterials, bioenergy and medicines, phytoremediation, biosentinel, tissue-engineering, and space exploration.

#### 4.2.1. Plant Biosystems Design for Increasing Photosynthetic Efficiency

The average yields of staple crops currently increase at a rate of about 1% each year, but this will need to increase by two-fold to feed the estimated world population of 9 billion people in 2050 [7]. Improvements in the yield potential of crops could be accelerated through genetic engineering approaches to enhance photosynthetic efficiency. A number of strategies have been employed to achieve this goal (for a detailed discussion see Long et al. [192]), with several recent successes that highlight our capacity to modify both the light-dependent and light-independent reactions of photosynthesis, both of which are important in determining crop yield potential. Here, we discuss some of these strategies and successes that could be further complemented by a plant biosystems design approach.

The photosynthetic reaction center complexes that capture light energy in plants (i.e., photosystem I (PSI) and photosystem II (PSII)) utilize only half of the incident solar energy (i.e., 400 to 700 nm) and work in series, connected by an electron transport chain. Redesign of the photosystems to expand the region of photosynthetic absorption from the visible region of the spectrum to include far-red and infrared regions could improve the efficiency of light capture [193]. For example, introducing novel light-harvesting pigments (e.g., chlorophylls *d* and *f* from cyanobacteria *Acaryochloris marina* and *Halomicronema hongdechloris*, respectively) could allow for light capture up to 750 nm [194]. In a more ambitious design, it would conceptually be feasible to redesign several components of the photosynthetic electron transport chain to harvest light<1000 nm [195]. Thus far, experimental successes to improve light-use efficiency and plant yields have come from overexpressing components of the cytochrome *b_6_f* complex, which facilitates the transfer of electrons from PSII to PSI [196, 197]. Furthermore, accelerating the repair of photodamage to PSII, through nuclear overexpression of the core PSII subunit protein D1, can improve photosynthesis and plant productivity and enhance survival under heat stress [198]. Lastly, engineering the photoprotective mechanisms of the photosystems can also facilitate growth enhancements. Photosystems dissipate excess absorbed light energy as heat in full sunlight but do not adapt to fluctuating light conditions rapidly, resulting in suboptimal photosynthetic efficiency and consequently losses of up to 20% of potential yield in field crops. This issue has been addressed by accelerating the induction and recovery from photoprotection in tobacco via bioengineering of an accelerated response to natural shading events, which increased dry biomass yield by ~15% in fluctuating light [199]. In contrast, the same approach in *Arabidopsis* has led to growth impairments, suggesting that the success of this strategy requires careful balance so as not to interfere with other regulatory processes [200].

For the light-independent reactions of photosynthesis (i.e., CO_2_ capture and conversion to sugars and starch), ribulose-1,5-bisphosphate carboxylase/oxygenase (Rubisco) is the key enzyme for CO_2_ fixation in all plants. Considering its importance, Rubisco is surprisingly inefficient and considered a bottleneck to photosynthetic productivity. Rubisco is a relatively slow enzyme, so to compensate, most plants invest *ca*. 30% soluble protein in leaves to the Rubisco pool. Furthermore, the dual specificity of Rubisco for CO_2_ and O_2_ results in two separate reactions: carboxylation and oxygenation. The latter results in the production of the toxic intermediate phosphoglycolate that is removed by the photorespiratory salvage pathway, causing the loss of carbon and energy. Photorespiration is widely regarded as a necessary but wasteful biochemical pathway [201]. Rubisco is one of the most well-studied enzymes and has been a prime engineering target for decades [202]. However, Rubisco consists of subunits expressed in the nuclear and chloroplast genomes and requires several chaperone proteins for functional assembly. Thus, engineering the catalytic properties of Rubisco has been challenging and progress has been slow. However, recent success in the assembly of *Arabidopsis* and tobacco Rubisco in *E. coli* could pave the way for more rapid screens to identify mutants with substantial improvements in function [52]. As an alternative strategy to combat Rubisco oxygenation, several photorespiratory bypasses in the chloroplast have been developed [203, 204] including a synthetic glycolate metabolism pathway that has been shown to increase tobacco biomass yield by ~40% in a field study [205]. Recent work has expanded this approach to rice and highlighted the need for readdressing source-sink flow in plants engineered to have enhanced photosynthetic potential [206]. Several alternative synthetic bypass routes have also been suggested, including a synthetic pathway for converting glyoxylate to hydroxypyruvate in the peroxisomes, which circumvents the mitochondrial reactions, avoiding decarboxylation and deamination [207], and enzyme engineering approaches to perform new-to-nature reactions such as the reduction of glycolate to glycolaldehyde [61]. More ambitious synthetic strategies include the development of alternative carboxylases and synthetic cycling pathways (i.e., not integrated into the Calvin-Benson-Bassham cycle) for CO_2_ assimilation that completely bypass the shortcomings of Rubisco [208–210]. Computational modeling could be used to design further novel synthetic pathways to assimilate CO_2_ and bypass photorespiration. Natural evolution in plants has only explored a fraction of the potential metabolic design space to drive photosynthesis [211, 212]; thus, there are likely many opportunities to further redesign and enhance crop performances.

Several photosynthetic organisms have evolved CO_2_-concentrating mechanisms (CCMs) to overcome the limitations of Rubisco and reduce photorespiration [213]. Plants have evolved two CCM pathways: C_4_ photosynthesis and crassulacean acid metabolism (CAM) photosynthesis. Although the potential for exploiting CAM photosynthesis for future agricultural production has been highlighted [214], most engineering work has focused on the benefits of C_4_ photosynthesis. Plants that perform C_4_ photosynthesis (i.e., C_4_ plants like maize, sorghum, sugarcane, and switchgrass) typically separate CO_2_ fixation across two cells types: initial capture of CO_2_ in mesophyll cells as the C_4_ acid oxaloacetate and conversion to malate, and then transport to Rubisco-laden bundle sheath cells, where malate is decarboxylated to release CO_2_. C_4_ photosynthesis facilitates above atmosphere local concentrations of CO_2_ around Rubisco, thereby favoring the carboxylation reaction over the oxygenation reaction and reducing photorespiration. As a result, C_4_ plants generally have higher photosynthetic efficiencies than C_3_ plants. Nevertheless, several important staple crops (e.g., rice, wheat, and soybean) are C_3_ plants. International efforts have been undertaken to engineer C_4_ photosynthesis into rice to increase photosynthetic efficiency and productivity [215]. However, due to the two-cell complexity of C_4_ photosynthesis, converting C_3_ into C_4_ photosynthesis requires considerable reengineering of metabolism and dramatic changes in leaf anatomy, both of which impose a significant challenge. Engineering a single-celled C_4_ system using biosystems design could be a promising alternative strategy [216]. In addition, introducing the single-celled physical CCMs found in algae and cyanobacteria into plants is predicted to lead to some of the largest improvements in yield potential (>60%) [217, 218]. Promisingly, several of the components required to build such systems have now been successfully introduced into plants [219–221]. Recently, the draft genome sequence of a single-cell C_4_ (SCC4) plant species, *Suaeda aralocaspica*, became available [222], providing an excellent genomics resource for engineering SCC4 in C_3_ plants. C_2_ photosynthesis, which utilizes glycine decarboxylase activity in the bundle sheath to decarboxylate the photorespiratory glycine produced in the mesophyll and deliver CO_2_ around Rubisco, is another CCM that operates by capturing, concentrating, and reassimilating CO_2_ released by photorespiration, and therefore, engineering of C_2_ photosynthesis has the potential to improve photosynthetic performance under high temperature, bright light, and low CO_2_ conditions [223, 224].

Roughly 50% of the carbon captured by photosynthesis (net of photorespiration) is subsequently lost, and strategies for cutting this large carbon loss include (1) reducing unnecessary turnover of proteins (e.g., THI4 which is a suicide enzyme with a very high turnover rate) and membranes; (2) replacing, relocating, or rescheduling metabolic activities (e.g., replacing the Phe route to lignin, relocating nitrate assimilation from root to shoot, and rescheduling biosynthetic processes from night to day); (3) suppressing futile cycles (e.g., futile cycles between sucrose synthesis and degradation or between fructose-6P and fructose-1,6BP); and (4) reducing ion transport costs (e.g., reducing efflux of nitrate to the rhizosphere) [225]. These strategies can be implemented through synthetic metabolic engineering approach [226].

#### 4.2.2. Plant Biosystems Design for Increasing Plant Stress Tolerance

Abiotic stresses (e.g., drought, heat, and salt stress) account for more than 60% of the yield loss in some major crops such as maize, wheat, rice, and soybean [227, 228]. Plant resistance to abiotic stresses can be divided into escape, avoidance, and tolerance [229, 230]. Various types of genes have been proposed as candidates for engineering to increase tolerance to abiotic stresses, including genes encoding (1) enzymes for production of protective metabolites (e.g., proline and sugars), (2) enzymes for membrane lipid biosynthesis, (3) enzymes for biosynthesis of antioxidants (e.g., ROS scavenging), (4) protective proteins (e.g., LEAs and molecular chaperones), (5) transporters (e.g., water and ion transport), (6) regulatory proteins, (7) kinases, and (8) proteins regulating transcription (e.g., transcription factors), along with genes involved in posttranscriptional (e.g., microRNAs) and posttranslational (e.g., ubiquitination) regulation of abiotic stress responses [230]. Previous efforts have been focused on engineering of individual genes to enhance tolerance to a specific abiotic stress. Plant biosystems design has the potential of integrating multiple genes to confer resistance to a broad range of abiotic stresses. Also, tissue-specific and stress-inducible expression of genes relevant to abiotic stress tolerance could be implemented via biosystems design to reduce energy costs and avoid pleiotropic effects.

One important example of stress avoidance mechanism is CAM photosynthesis, which is a natural solution to the challenge caused by drought stress. CAM plants close their stomata (the pores on the leaf surface) during the heat of day and open them at night, resulting in lower water loss and higher water use efficiency than C_3_ or C_4_ plants, which close their stomata during the nighttime and open them during the daytime [231]. The maximum yield of CAM crops is much higher than that of C_3_ or C_4_ crops under water-limited conditions [232]. Engineering of CAM machinery into C_3_ or C_4_ plants has great potential for increasing crop yield under drought conditions. CAM engineering requires design of multiple gene modules involved in carboxylation, decarboxylation, and stomatal movement, as well as genes involved in leaf succulence and vacuole size [233–235]. Biosystems design approaches could be used to integrate these CAM-related gene modules into plants, preferably using gene circuits to establish drought-inducible CAM (or CAM-on-demand) systems [236].

Biotic stresses imposed by pathogens and pests can also cause massive losses in crop yield. Engineering synthetic plant immunity would be a promising strategy for increasing or broadening plant resistance to diseases [237]. Immune receptors, such as nucleotide-binding leucine-rich repeat (NLR) receptors, are promising targets for increasing disease resistance using biosystems design approaches [238, 239]. Creating genetically modified crops resistant to insects is a useful approach to reduce the yield loss caused by pests [240], such as transgenic crops overexpressing *Bacillus thuringiensis* (*Bt*) insecticidal proteins [241]. It is critical to design gene constructs that specifically target pests without toxic effects on humans or negative impacts on beneficial organisms. Host-induced gene silencing (HIGS), in which double-stranded RNAs (dsRNAs) directed against suitable insect target genes are expressed in transgenic plants, has been used to confer protection against pests [242]. The HIGS approach has two major advantages: (1) dsRNAs can be designed to be highly specific to target insects without negative impact on other organisms, and (2) multiple dsRNAs can be engineered into each individual plant for protection against multiple pests. However, the design of HIGS requires rich genomics resources of target insects and related species.

Beneficial microbes (e.g., bacteria and fungi) can enhance plant resistance to abiotic and biotic sources of stress [243]. Plants can generate molecular and metabolic effectors for promoting beneficial plant-microbe interactions. Synthetic genetic circuits can be engineered into plants to reshape the rhizosphere microbiome to enhance stress tolerance and acquisition of nutrients (e.g., nitrogen). For example, opine biosynthesis pathways have been engineered into plants to reshape rhizosphere populations to increase the population densities of opine-catabolizing bacteria [244]. Similarly, a synthetic pathway has been engineered in *Medicago truncatula* and barley for the production of the rhizopine *scyllo*-inosamine to regulate bacterial gene expression in the rhizosphere [245]. There is considerable potential for engineering host plants to promote the beneficial interactions between plants and microbes.

#### 4.2.3. Plant Biosystems Design for Improving Food Crop Quality

The ever-increasing living standard worldwide, combined with limited arable land availability, calls for genetic improvement of food crop quality. Deficiencies in vitamins collectively affect billions of people worldwide and are a cause of substantial morbidity and mortality. Vitamin A deficiency is the global leading cause of preventable blindness [246], iron deficiency delays cognitive development [247], and folate deficiency is especially common among pregnant women and is associated with defects in fetal neural crest development [248]. Eliminating vitamin deficiency is a global public health priority and one of the World Health Organization Millennium Development Goals [249]. Biofortification (i.e., improvement of nutritional quality of food crops during plant growth and development) is a cost-effective strategy to mitigate vitamin deficiencies, particularly in the developing world where other vitamin supplementation programs suffer from logistical problems with transportation. Plant biosystems design is a useful approach to achieve biofortification through the engineering of superior nutritive properties in crop plants. Examples of biofortified crops include those enhanced with beta-carotene (provitamin A) [250, 251]; arachidonic acid [250]; carotenoids associated with eye and cardiovascular health, immunity, and cognitive function [250, 252]; iron [253]; and folate [254], along with efforts underway for *α*-tocopherol (vitamin E) [255] and zinc [256].

Crop plants generally contain various types of antinutritional factors such as cyanogenic glycosides (e.g., phaseolunatin and dhurrin), enzyme inhibitors (e.g., alkaloids and protease inhibitors), physiological disorganizers (e.g., lectins and saponins), hormone biosynthesis inhibitors (e.g., goitrogens), and antivitamins (e.g., antivitamin E) [257, 258]. For example, cowpea (*Vigna unguiculata* L. Walp) plants, which are able to grow in semiarid regions with low input requirements, provide a sustainable source of essential nutrients (e.g., high protein and low fat content), but dietary utilization of cowpea has been seriously constrained by its antinutrients (e.g., phytic acid, protease inhibitors, and cyanogenic glucosides) and low protein digestibility [259]. Altogether, biosystems design could simultaneously leverage biofortification, remove antinutritional compounds, and increase protein digestibility, but care needs to be exercised to not consequently increase pest and pathogen susceptibility.

#### 4.2.4. Plant Biosystems Design for Mitigation of Climate Change

Carbon dioxide released into the atmosphere is the primary cause of anthropogenic global warming [260, 261]. Terrestrial plants are a major player of atmospheric CO_2_ capture and storage [262, 263]. Carbon sustainability and carbon neutrality would be the great benefit that can be achieved with faster growing plants. Biosystems design has great potential in CO_2_ capture and storage in various aspects, including (1) improving photosynthetic efficiency aboveground and allocation of photosynthates to below ground structures (e.g., source-sink modulation) [264], (2) converting annual crops to perennial plants that have much larger root systems [265], (3) generating more recalcitrant carbon-containing compounds (e.g., lignin and suberin) in the roots [266], (4) establishing deeper root systems [267], (5) enabling animal to plant-sourced protein shifts [268], (6) restoring forests [269], (7) enabling carbon mineralization [270, 271], and (8) increasing biomass accumulation through genetic improvement of photosynthetic efficiency [272] (see also Section 4.2.1). Armed with these biological targets, opportunities to mitigate rising atmospheric CO_2_ concentrations are many, with promising avenues to pursue through biological carbon capture and storage in soils. This undertaking should be viewed with increasing optimism, especially as the strategies and technologies—aided by plant biosystems design—employed to store carbon in soil pools with long residence times have emerged and will continue to evolve over time. The postgenomics era provides an unprecedented opportunity to identify genes, enzymes, biochemical pathways, and regulatory networks that underlie rate-limiting steps in carbon acquisition, transport, and fate; and thereby yield new approaches to enhance terrestrial carbon sequestration. An investment in plant biosystems design could harness these new approaches to increase biomass production in agricultural crops and fast-growing trees in managed plantations.

#### 4.2.5. Plant Biosystems Design for Bio-Based Materials

**Figure 12:**
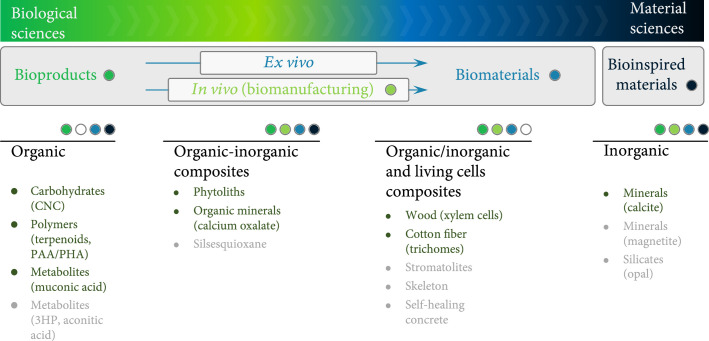
Diagram showing concepts and examples of bio-based materials from plants. Interdisciplinary gradient connects biological, engineering, medical, physical, and material sciences. Bioproducts are the building blocks of bio-derived materials, which can be manufactured outside (chemically) or inside (biologically) an organism through biomanufacturing. Each class is color coded to its major constituents (organic, inorganic, and/or living cells) with examples in plants (dark green) and elsewhere (light gray). CNC: cellulose nanocrystals; 3HP: 3 hydroxy-propionic acid; PAA: polyacrylic acid; PHA: polyhydroxyalkanoate.

Living organisms produce a series of proteins and compounds, i.e., bioproducts, used as building blocks for the manufacture of biomaterials. Such manufacture can take place *ex vivo* (e.g., through chemical manipulations of extracted bioproducts) or *in vivo*, being naturally synthetized by the living organism (i.e., biomanufactured). Bio-derived or bio-based products or materials are simply referred to here as “biomaterials”. Importantly, these concepts are independent from bioinspired materials, which are characterized by the application of biological design rules and principles by material scientists during synthetic (nano)material synthesis (Figure 12).

Bioproducts come from organic, inorganic, and/or living cell sources, which can be “mixed and matched” to promote composites with a diverse set of properties and modularity often unavailable to chemists and material scientists [273]. Plants are a prime source for various bioproducts, including (1) biopolymers such as cellulose, lignin, and derivatives; (2) extractives such as latex, starch, and fatty acids (e.g., polyhydroxyalkanoates); (3) small molecules such as phenylacetic acid and muconic acid, which often undergo additional downstream chemical, enzymatic, and/or thermal processing for commercial applications; (4) inorganic biominerals such as phytoliths; and (5) organic-inorganic composites such as calcium oxalate crystals and calcium carbonate. Plant-derived bioproducts can be used for fiber, bioplastics, liquid crystals, energy storage, and insulants [274–283]. Their applications span various fields, including medicine, engineering, and material sciences, representing complementary and replaceable alternatives to bioproducts and biomaterials from animal origin, which can draw environmental and ethical concerns.

One beneficial template example for biomanufacturing is the composite of calcium carbonate (CaCO_3_), which is a plant crystal equivalent to nacre (mother-of-pearl) in composition. However, their multifactorial architecture is different, with plant crystals being good insulators and nacre having unique mechanical and optical properties. Exploring plant biosystems design with multidisciplinary tools could enhance our predictive power to modify such plant crystals, programming a set of characteristics to solve urgent energy, engineering, and environmental problems [284–289].

Another example, in biomaterials fabrication, is the bioengineering and use of cellulose nanocrystals (CNC) and nanofibrils (CNF). Structural and functional properties of CNFs can be influenced by plant cellulose properties, such as crystallinity and interfacial binding with matrix polysaccharides [290]. The relevance of biologically synthesized 18-(glucan) chain cellulose microfibril structure on CNF properties [290] opens up the prospect of leveraging the biosynthetic processes in plants. Potential biosystems design strategies for varying the chain length and crystallinity of cellulose include altering the distribution and composition of cellulose synthase (CesA) complexes (CSCs), which have been proposed to be composed of 18 CesA proteins [291], to potentially optimize cellulose-derived nanocellulose and nanocellulosic composites. Uses of conventional approaches to enhance cellulose content via overexpression of single secondary wall-associated CesA types have generally not resulted in increased cellulose content in woody plants due to challenges with effective transgene expression in the presence of endogenous copy [292] or varying CesA stoichiometry needed for functional CSCs [293]. Use of biosystems design approaches will aid in understanding the extent to which domain swapping, modified CSC composition, heterologous CesA expression, and use of optimized promoters can impact content, crystallinity, degree of polymerization, and crosslinking properties of cellulose.

In use of biomaterials in clinical contexts, scaffolds of animal origin can lead to variability and environmental and ethical concerns, which can be potentially addressed using animal-free scaffolds such as bioproducts of plant origin [278]. Due to the natural strength of plant and marine algae-derived bioproducts (e.g., nanocellulose and alginate) and their functional roles as scaffolds for growth, plant-derived products, or materials are highly promising as bioinks for printing of novel biomaterials with applications in drug delivery, wound healing, and implantable medical devices [279].

Overall, biosystems design can be a powerful aid in accelerating the research and development of plant-derived bioproducts. Although plants have been the source of biomaterials for a long time, the recent need for petroleum-independent products, associated with the tremendous opportunities and potential for developing renewable and better performing products from biomaterials, has accelerated studies in biomanufacturing [286]. Also, *in vivo* biomanufacturing provides us with materials having characteristics unable to be reproduced by chemistry alone [273]. However, we still lack understanding of the “material loci” in plants controlling the synthesis, transport, modification, assembly, and storage of biomaterials. In addition, there has been limited exploration of chemical composition, ultrastructure, and bonding within/across interfaces in hybrid biomaterials, which often have appealing physical, optical, and electromagnetic properties. Biosystems design approaches will be required for leveraging such needs as well as recruiting and integrating emerging theoretical, computational, and *in situ* characterization tools to establish a knowledge toolbox and bridge gaps between disciplines, accelerating the overall biomaterial cycle “design-discover, synthesize, characterize, learn and apply” (DiSCLA). For example, a biosystems design approach has been proposed to reconfigure plant metabolism for cost-effective production of biodegradable plastic [294]. One challenge of this approach is how to minimize the negative impact of biodesign for bio-derived products and materials on plant growth performance both above and below ground [264, 294].

#### 4.2.6. Plant Biosystems Design for Bioenergy Production

The interest in biofuels has largely shifted from bioethanol to drop-in advanced fuels due to the increasing popularity of electric automobiles, which has shifted the major potential for biofuels into aviation, where the use of heavy batteries remains unlikely to become economically feasible. Because energy density is a key consideration for aviation fuels, drop-in fuels are more promising than bioethanol [295]. Current bioethanol production suffers from a number of problems such as relatively small lifecycle reductions in greenhouse gas emissions [296] and a competition with food production that raises the price of staple foodstuffs [297, 298]. In order to overcome these drawbacks, bioenergy crops should be engineered to grow with fewer inputs, on marginal land, or with valuable coproducts such as food, medicine, or industrial chemicals [299]. Biomass feedstocks (e.g., lignocellulose, starches, and lipids) can be converted into jet fuels via several different routes, such as oil to jet fuels (OTJ), syngas to jet fuels (STJ), and alcohol to jet fuels (ATJ) [300]. Currently, the cost of biomass-based jet fuels is relatively high (e.g., 4.4 to 5.1 $/gal from the OTJ route), which can be offset by generating high-value coproducts from the biomass feedstock [300]. Therefore, it is important to design the metabolic pathways in plants for optimizing biomass feedstock for production of both jet fuels and value-added coproducts. Previous efforts have discovered a lot of genes relevant to yield and quality of biomass in multiple bioenergy crops. Biosystems design provides an excellent opportunity for combining the improved traits conferred by individual genes to optimize the performance of bioenergy crops, with simultaneous improvement of biomass quality (e.g., high cellulose content and low recalcitrance to deconstruction) and biomass accumulation under both normal and stress conditions.

Typically, plants have a low photosynthetic efficiency, converting less than 1% of the available sunlight to stored chemical energy [301], which limits the economic feasibility of plant biomass as feedstock for biofuels production [302]. Therefore, it is critical to increase the photosynthetic efficiency of bioenergy crops using biosystems design approaches, as described in Section 4.2.1. In general, the stem of woody bioenergy crops (e.g., poplar) is used for biofuels production while the leaves are discarded as waste. To increase the economic value of woody bioenergy crops, their leaves can be used as bioreactors to produce high-value bio-based products (e.g., biodegradable plastics and specialty or commodity chemicals) using the strategies described in Section 4.2.5 as well as medicine using synthetic biology approaches described in Section 4.2.8. Furthermore, the below-ground tissue of bioenergy crops can be optimized for long-term carbon storage using biosystems design to mitigate climate change (see Section 4.2.4).

#### 4.2.7. Plant Biosystems Design for Phytoremediation and Phytomining

Pollution by heavy metals, which cannot be chemically degraded, poses a serious long-term threat to the environment and human health [303]. Some plants, called hyperaccumulators, can accumulate metal and metalloid trace elements (e.g., nickel, zinc, cadmium, manganese, arsenic, and selenium) to extraordinarily high concentrations in their above-ground living biomass [304]. There are 721 plant species identified as hyperaccumulators [305], and some of the hyperaccumulator plants can take metals up to 1-2% of total dry weight, which may be hundreds or thousands of times greater than commonly grown plants [305, 306]. In comparison with different physical and chemical methods of extracting heavy metals, use of hyperaccumulator plants is perceived as a green, low-cost, and efficient approach [307]. Hyperaccumulator plants can be utilized for phytoremediation to clean up the soils contaminated by heavy metals and/or for phytomining to recover an economic amount of metals (e.g., nickel) from the plants [303, 308].

Comparative analyses of hyperaccumulators and closely related nonhyperaccumulators have improved the understanding of molecular mechanisms of heavy metal uptake, transport, sequestration, and tolerance in hyperaccumulators. Particularly, functional characterization of heavy metal transporters, such as Zinc-regulated transporter Iron-regulated transporter Proteins (ZIP), Heavy Metal transporting ATPases (HMA), Multidrug And Toxin Efflux (MATE), and Metal Transporter Proteins (MTP) gene families, has yielded valuable gene resources for designing and engineering more effective phytoremediation systems [306, 309]. Overexpression of such transporters has been widely successful in enhancing the uptake of heavy metals in model plants or nonhyperaccumulators [306]. However, due to the complexity of plant metal transporting and trafficking systems, a much more sophisticated design of plant biosystems will be required to enhance the capability of phytoremediation.

A nascent area of interest is the role of plants in accumulation of rare earth elements (REEs), which are critical materials with unique light, catalytic, and magnetic activities but do not have reliable supply chains. Plants generally have low concentrations of rare earth elements, which in part reflect diffuse distribution and low concentration of rare elements in soil. However, certain types of plant-like ferns and citrus trees have been reported to have a higher capacity for accumulating rare earth elements [310–312]. Future research on phytomining can focus on the understanding of the molecular mechanism underlying REE accumulation in hyperaccumulating plants and then transfer the REE-accumulating mechanism into the existing crop plants using biosystems design, which may involve engineering of transporters and metabolic pathways.

Biosystems design research for phytoremediation and phytomining can be focused on the following aspects: (1) due to the narrow distribution and low biomass yield of hyperaccumulator plants [305], it is necessary to extend the geographic distribution and biomass yield of existing hyperaccumulators through targeted mutation or gene circuit design; (2) it is important to enhance the uptake and tolerance of heavy metals in nonhyperaccumulators using a biosystems design approach; (3) because hyperaccumulation of lead, copper, cobalt, chromium, and thallium has not been well established in natural plant systems yet [304], it is urgent to either find natural or synthetic genes and pathways for accumulating these heavy metals through systems biology research, *in silico* modeling, and metal transporter protein engineering; and (4) comparative cross-species studies can unravel the fundamental pathways unique to hyperaccumulators of rare earth elements and present avenues for potential biosystems design approaches in deployable plant species.

#### 4.2.8. Plant Biosystems Design for Medicine Production and Medical Research

Plants have been the primary production chassis for medicine for millennia and continue to play an important role in modern supply chains. Plants are the source of approximately 25% of modern drugs and in many cases remain the most cost effective method for their production [313]. Examples include the antimalarial drug artemisinin [314] and the anesthetic morphine [315]. Many of these high-value medicinal compounds are produced from nonmodel plants, but plant engineering efforts have nonetheless been successful in improving their yield [316], which remains economically competitive with chemical and microbial synthesis [317].

Plants have been used as bioreactors to produce vaccines, such as anticancer or viral vaccines [318]. Genetically engineered plants can produce recombinant proteins at larger scale than conventional platforms and are on track to become cost competitive with conventional production platforms. In fact, plants are being used as a platform to produce a wide range of antibodies in different organs (leaves, roots, seeds, tubers, fruits) of various plant species, such as tobacco, potato, rice, tomato, and pea [319–322]. This has been possible due to rapid improvement in plant genetic engineering and transformation technologies. There are several benefits for the use of plants to produce therapeutic antibodies. First, it reduces the production cost. Plants can be used to produce recombinant proteins at 0.1% and 2–10% of the cost of mammalian cell cultures and microbial fermentation systems, respectively [323]. Second, plants are usually regarded as safe systems for antibody production because they do not harbor mammalian pathogens or produce endotoxin. Finally, production of antibodies in edible tissue will allow convenient, needle-free oral immunization at the gastric mucosal surface [320].

There are several examples of successful use of a plant-based antibody to treat human diseases. One such case is that the secretory antibody “CaroRxTM” derived from tobacco leaves was used to treat Ebola patients during outbreaks of this virus in Africa in 2014 [324]. Other plant-made antibody products that are currently being used to cure human diseases are “DoxoRxTM” for treating drug-induced alopecia, a common side effect of cancer therapy and RhinoRxTM for treating the common cold [320, 325]. Another outstanding example of plant-based molecular pharming to produce biopharmaceuticals is the production of vaccine against the SARS-CoV-2 virus. A biotech company called Medicago Inc. (Quebec City, Canada) is using plants to produce virus-like particles (VLPs), which is the first step in developing a vaccine against COVID-19 before preclinical testing for safety and efficacy. VLPs are the noninfectious viral proteins that lack the key genetic materials for infection. However, they are still recognized by the immune system and therefore can be used to produce antibodies against the SARS-CoV-2. A similar effort is also underway by a US-based company iBio, Inc.

Plants can also be used to produce reagents for detecting human pathogen. For example, plants can be used to generate diagnostic reagents for COVID-19 in multiple ways: (1) generating positive control reagents for RT-PCR detection of SARS-CoV-2 virus by producing artificial RNA containing the target virus genomic regions which are packed inside the VLPs derived from Cowpea mosaic virus (CPMV), (2) generating antibodies for detecting the spike (S) protein of SARS-CoV-2, and (3) generating recombinant proteins for detecting antibodies against SARS-CoV-2 to identify people who are currently infected or recovering from infection [326].

In addition to serving as chassis for producing medicinal compounds and biologics, plants are emerging as a robust discovery and functional validation platform to elucidate the genetic basis of heritable human diseases including cancers and developmental abnormalities. Plants and humans share a common eukaryotic ancestry represented by evolution of the first complex and multicellular organism. Evolution of this complex life form necessitated the emergence of genetic mechanisms to coordinate DNA replication-repair, cell division, signaling between neighboring cells, and their adhesion to facilitate hierarchical assembly into tissues and organs with specialized functions [327]. Given this shared ancestry, proteins underlying basal processes such as DNA replication-repair, cell division, and cell adhesion remain highly conserved across disparate eukaryotes. Additionally, such conservation at the protein level has been shown to result in orthologous phenotypes (or phenologs) across divergent eukaryotes including *Arabidopsis*, humans, and mice [328], suggesting that comparative studies across eukaryotes have the potential to identify critical amino acid motifs mediating function of these conserved proteins.

Although significant progress has been made in linking mutations to disease outcomes in human populations using genome-wide association studies (GWAS), pin-pointing causal variants that can be used as reliable biomarkers in disease diagnosis remains a major challenge. This is largely due to the fact that heritable diseases are often extremely rare in human populations which limits the ability to acquire sufficient sample sizes for robust statistical associations [270]. In contrast, long-lived perennial plants like poplar (*Populus* spp.) exhibit wide ranges of phenotypic variation that is underpinned by their ability to maintain diverse sequence variants, including high impact loss-of-function mutations, at surprisingly high levels [329]. As such, high-resolution GWAS in plants often require fewer samples and can precisely identify causal mutations underlying phenotypic expression. For example, using ~300 individual poplar plants, Tuskan et al. [330] demonstrated that shared homology at the protein-level was manifested as an orthologous cell-proliferation phenotype between the poplar and humans. Specifically, they found significant similarities between genes implicated in callus formation in poplar and tumorigenesis in humans. Callus formation, which is the rapid growth of undifferentiated cell masses in plants, is orthologous to the uncontrolled cell proliferation during tumorigenesis in humans. The rate of callus formation was significantly associated with loss-of-function mutations occurring within a poplar SOK1 kinase related to the Mammalian Sterile-20 kinase, which has been shown to function in tumor suppression in humans [331]. In a separate study, Bdeir et al. [332] identified sequence variants in a desmosome protein, which were associated with adhesion of bark tissue in poplar. A functional variant of the protein prevented bark abscission resulting in annual accumulation of bark layers. In humans and mice, orthologous function of the same protein has been implicated in onset of the extremely rare skin cancer, keratoderma, which is manifested as an abnormal accumulation skin layer [333].

Given these unique advantages in precise genetic mapping of causal variants in addition to ease of experimental manipulation as well as fewer ethical issues, plants offer an attractive discovery and validation platform for understanding how mutations modulate the function of proteins that are fundamentally conserved across eukaryotes to provide highly resolved therapeutic targets for treatment of heritable human diseases in the rapidly expanding precision medicine field [334].

#### 4.2.9. Plant Biosystems Design for Biosentinel

Biosensors are defined as molecules, organisms, or devices in a biological context that emit quantifiable signals in response to specific molecules or biological processes [335]. Plants have rapid (within the seconds to minutes time-scale) responses to various biotic and abiotic stimuli [336]. Therefore, inducible expression of reporter genes, such as the genes encoding fluorescent protein (e.g., GFP) and pigments (e.g., anthocyanin and chlorophyll), could be engineered to detect environmental stimuli. For example, a resettable synthetic degreening plant-based biosensor (also called phytosensor) system was successfully created, in which plants lost their chlorophyll from induction of the degreening circuit by a synthetic steroid (4-hydroxytamoxifen, 4-OHT), and regreened after the inducer was removed [337]. Also, a synthetic signal transduction pathway was constructed for detecting the nitroaromatic explosive 2,4,6-trinitrotoluene (TNT) [338].

#### 4.2.10. Plant Biosystems Design for Tissue Engineering

Synthetic morphogenesis enabled by synthetic biology can be achieved for the *de novo* generation of programmable tissues and organs [339]. Three-dimensional microstructure has recently been created from single plant cells *in vitro* by mimicking the plant tissue environment and using biocompatible scaffolds similar to those used in mammalian tissue engineering, with the scaffolds providing both developmental cues and structural stability to isolated callus-derived cells grown in liquid culture [340, 341]. Furthermore, one interesting science-fiction-like question is: Can plants be genetically reprogrammed to form new-to-nature structures useful for human life? For example, it has been envisioned that a tree could be potentially reprogrammed to grow into a fully functional house based on the genetic instructions designed by scientists [342].

#### 4.2.11. Plant Biosystems Design for Space Exploration

There are growing interests and ongoing plans for human to travel to Mars in the near future, and plants on Earth need to be redesigned for meeting the needs of humans living in the Martian environment [343]. The targets include increasing drought-resistance to allow for plant growth under water scarcity in an extraterrestrial environment [344], cold tolerance, nutrient-utilization efficiency, and adjusting photosynthetic tuning to optimize for the lower Martian light intensity [343]. For practical reasons, it would be better to engineer plants for survival and yield in a space station and protected facilities on the surface of other celestial bodies (e.g., controlled environment like growth chambers), rather than in the open space on the surfaces of those bodies. The Martian surface temperatures are generally between -60 and 0°C and are not suitable to plant growth without radical engineering out of reach of current technology.

## 5. Social Responsibility of Plant Biosystems Design

Biosystems design is highly powerful for plant science research with enormous promise and needed caution. While plant biosystems design has great potential for genetic improvement of crop plants or creation of synthetic plant genomes for the benefit of our society, this new research discipline has a huge social responsibility to ensure biosafety and address the potential ethical issues.

### 5.1. Safety of Plant Biosystems Design

Engineered plants show enormous potential and have been repeatedly shown to be safe by a large number of scientific organizations, including the World Health Organization (https://www.who.int/health-topics/food-genetically-modified#tab=tab_2) and the American Association for the Advancement of Science (https://www.aaas.org/news/statement-aaas-board-directors-labeling-genetically-modified-foods). The Society of American Foresters supports and encourages the use of appropriately regulated Genetically Modified Organisms (GMOs) (https://www.eforester.org/Main/Issues_and_Advocacy/Statements/Regulation_of_Genetically_Modified_Trees.aspx). Nonetheless, plant biotechnology still presents some potential risks that must be considered during plant biosystems design research. These risks can be grouped into six broad categories: nutritional nonequivalence, potential allergenicity, escape of noxious transgenes, creation of resistant weeds and pathogens, unintended changes, and disruption of ecosystem function. Strategies are available to reduce each of these risks but must be implemented through the design, testing, and implementation phases of plant biosystems design in order to effectively manage the risks. While there is a great concern about the potential safety issues caused by transgenes, the risks of not using transgenes have been ignored. It is critical to evaluate the risks of using vs. not using transgenes. For transgenes which are not noxious, their risks can be considered very low if there are no unintended changes (e.g., side effects and off-target effects) caused by the transgenes. Risk analyses should focus on the engineered plant products, not the process through which they are created (e.g., CRISPR/Cas-based gene editing vs. transgenesis). Therefore, genome-edited crops are not regulated as GMOs in more and more countries since genetic variants created through genome editing are indistinguishable from naturally evolved variants [345], although they are still categorized as GMOs in Europe, with concerns on unintended effects (e.g., off-target effects, unintended on-target effects, and other unintended consequences) [346] (see Section 5.1.5 for more details).

#### 5.1.1. Nutritional Nonequivalence

Nutritional nonequivalence is frequently a design feature for transgenic crops, as in the case of biofortified cereal grains [347, 348]. However, there is always the possibility during biosystem engineering of unintended changes to the crop product metabolome, which may result in lower nutritional quality. Careful quantification of compounds metabolically linked to any plant biodesign changes should be carried out under a variety of field conditions to test for changes to nutritional content or production of harmful levels of unintentional off-target metabolites. Flux analysis is a biosystems design tool that can guide the search for relevant metabolites to be analyzed [349].

#### 5.1.2. Potential Allergenicity

Introduced allergenicity is another risk that arises naturally from a design feature. If genes encoding allergenic proteins are introduced into a plant, the resulting engineered plant is likely to act as an allergen. The generation of allergenic plants occurred early in the history of transgenic plant production with soybeans encoding the 2S albumin protein from Brazil nut [350]. Allergies to Brazil nut 2S albumin are relatively common and often severe, so it is likely that introduction of the allergenic soybean to the human food supply would have harmed some consumers, leading to a public relation disaster. Fortunately, the product was never brought to market, but this work acts as a reminder of the seriousness of this issue [351]. All transgenic proteins introduced to crops should be rigorously tested with a library of immune cells and serum samples to ensure no known or unknown allergens are being added to crops.

#### 5.1.3. Potential Escape of Noxious Transgenes

Engineered plants containing transgenes are broadly useful for agriculture, with transgenes intended to remain within the specific cultivars for which they are designed. However, multiple instances have been documented of “transgene escape,” which occurs when transgenes are transferred to other crop cultivars or wild relatives [352, 353]. Transgene escape is widely discussed by opponents of plant biotechnology and could potentially interfere with wild gene pools, generating agronomic problems such as herbicide resistant weeds. Steps should therefore be taken to reduce transgene escape rates. Such methods include the use of transgenesis in the plastid genome rather than the plant nuclear genome, resulting in maternal rather than biparental inheritance [354], self-limiting genes that result in eventual sterilization of the plants [355], male sterility [356], and bisexual sterility [357].

#### 5.1.4. Emergence of Herbicide and Pesticide Resistance

Agricultural fields are usually designed with extremely high densities of genetically similar plants and high levels of nutrients, which are ideal conditions for the spread of disease, parasitism, or opportunistic growth by undesired plants. In the ongoing battle against pathogens, pests, and weeds, new tools tend to be effective for a length of time before evolution of the undesired organisms renders the tool useless [241, 358]. The situation is analogous to the problem of antibiotic resistance in medicine, and the balance will likely continue until the elimination of agriculture or the elimination of the undesired pests, pathogens, and weeds. In some cases, transgene escape could lead to evolution of resistance to existing tools, but even if that case is prevented, evolution will take its course, and organisms will develop resistance to the current agricultural defenses. Agronomic and biotechnological steps can be taken to delay the evolution of resistance including the use of multiple independent forms of defense simultaneously (e.g., several distinct herbicides and herbivory deterrents) and the planting of undefended “refuges” to reduce the selective pressure for undesired organisms to evolve resistance to the crop defense chemical or engineered trait [359].

#### 5.1.5. Unintended Changes

CRISPR/Cas-mediated genome editing has been widely used for genetic improvement of plants. However, there are concerns about potential, unintended genetic modifications caused by gene editing due to the off-target effects. Although off-target mutations in CRISPR/Cas9-edited plants can be negligible and at a level lower than inherent natural variation when highly specific gRNAs are used [360], it would be prudent to carry out risk analysis for practical application or commercialization of genome-edited plants [361, 362]. For example, a recent study assessed, in addition to off-target mutations, potential epigenetic changes attributable to CRISPR-mediated genome editing, reporting no detectable changes on DNA methylation status in edited plants [363].

#### 5.1.6. Disruption of Ecosystem Function

While uncommon, there is a risk that transgenic plants have negative consequences on natural ecosystems. A classic example is the case of plants transformed with the genetic material from the bacterium *Bacillus thuringiensis* (*Bt*) and the monarch butterfly, in which a substantial research effort led by the US Department of Agriculture occurred following reports that monarch larvae had a decrease in survival following feedings on milkweed dusted with *Bt* pollen [364]. Although it was determined that only a very small portion of adjacent milkweed plants accumulate pollen in sufficient concentration to have a negative effect, it is important to recognize that there may be ecological risks associated with the release of transgenic plants.

### 5.2. Ethics of Plant Biosystems Design

As an emerging cutting-edge discipline, plant biosystems design should meet the following ethic principles: public beneficence, responsible stewardship, intellectual freedom and responsibility, democratic deliberation, and justice and fairness [365].

#### 5.2.1. Ethical Issues of Plant Biosystems Design

Plant biosystems design and plant biotechnology show great promise in improving environmental and human health. Continued funding for applied plant science is critical to ensure this promise is fulfilled, especially for cases without obvious commercial interests such as design of biofortified food for poor consumers in the developing world. Since national research organizations provide a substantial fraction of the funding for plant biosystems design research, there is a moral obligation to ensure that the benefits of the products are shared by as many people as possible.

#### 5.2.2. Solutions to Address the Ethical Issues

A well-known genetically engineered crop is Golden Rice, a widely celebrated biofortified crop that resulted from a major public-private collaboration [366]. The product was designed to benefit the global poor, and the licensing agreements regarding the underlying intellectual property are exemplary: while large scale farmers in Western countries must pay for use of the product, Golden Rice is free for those who need it, specifically breeding programs and smallholder farmers (http://www.goldenrice.org/Content1-Who/who4_IP.php). Such licensing arrangements can simultaneously give companies the required economic incentives to produce products and democratize access to those products to the global poor who stand to benefit most.

## 6. Conclusion

As an emerging interdisciplinary research field, plant biosystems design shows massive potential for not only increasing our understanding of the mechanisms underlying the biological complexity of plant organisms but also accelerating the domestication of crop plants or creation of novel plant organisms to address challenges related to food and energy security, environmental sustainability, and human health. In this roadmap, we discuss the principles, methods, applications, and social responsibility of plant biosystems design. Currently, the theories and principles of plant biosystems design are only partially understood, and the knowledge gaps are expected to be filled through systems biology research and the DBTL process(es) in the future. Significant progress has already been made in method development for plant biosystems design, such as efficient assembly of DNA parts, high-precision gene editing, enhancement of plant transformation using morphogenic regulators, and virus/nanotube-mediated *in planta* transformation. Still, new technologies will need to be developed for enabling large-scale genome refactoring or construction of functional synthetic plant genomes. Exciting achievements have been made in the application of biosystems design to pathway engineering for improving agricultural crops, yet the potential of biosystems design needs to be exploited in many other aspects, such as bio-based materials, climate change mitigation, bioenergy production, phytosensor, tissue engineering, and space exploration. The potential of new machine learning capabilities (e.g., explainable artificial intelligence) could be exploited for predictive learning from big genomics and phenomics data. Also, plant biosystems design will need to be deployed for a new type of high precision agriculture and bioeconomy as a contribution to the fourth industrial revolution, in which genetic engineering plays an important role [367–369]. While it will be very important to achieve the scientific and technological advancements in plant biosystems design, we should pay special attention to its social responsibility (i.e., biosafety and ethics) to improve the public perception and acceptance of this new research discipline as benefiting humanity and the environment. In particular, there is a need to engage private and academic stakeholders to bring all these technologies to the poorest populations. Finally, plant biosystems design is much more challenging than traditional genetic engineering and therefore needs extensive interdisciplinary collaborations among many researchers around the world. We hope that the three computational modules in the “Plant Biodesign Hub” (Figures 6, 7, and 9) outlined in this roadmap will serve as a major public platform for national and international collaborations to realize the great promise of plant biosystems design for the future of our global society.

## References

[1] S. Knapp, “People and plants: the unbreakable bond,” *Plants, People, Planet*, vol. 1, no. 1, pp. 20–26, 2019

[2] S. J. Hiscock, P. Wilkin, S. Lennon, and B. Young, “Plants matter: introducing plants, people, planet,” *Plants, People, Planet*, vol. 1, no. 1, pp. 2–4, 2019

[3] H.-Z. Chen, and Z.-H. Liu, “Steam explosion and its combinatorial pretreatment refining technology of plant biomass to bio-based products,” *Biotechnology Journal*, vol. 10, no. 6, pp. 866–885, 2015 25904087 10.1002/biot.201400705

[4] A. T. Austin, and A. E. Zanne, “Whether in life or in death: fresh perspectives on how plants affect biogeochemical cycling,” *Journal of Ecology*, vol. 103, no. 6, pp. 1367–1371, 2015

[5] M. E. Dusenge, A. G. Duarte, and D. A. Way, “Plant carbon metabolism and climate change: elevated CO_2_ and temperature impacts on photosynthesis, photorespiration and respiration,” *New Phytologist*, vol. 221, no. 1, pp. 32–49, 2019 29983005 10.1111/nph.15283

[6] M. J. M. Christenhusz, and j. W. Byng, “The number of known plants species in the world and its annual increase,” *Phytotaxa*, vol. 261, no. 3, pp. 201–217, 2016

[7] S. P. Long, A. Marshall-Colon, and X.-G. Zhu, “Meeting the global food demand of the future by engineering crop photosynthesis and yield potential,” *Cell*, vol. 161, no. 1, pp. 56–66, 2015 25815985 10.1016/j.cell.2015.03.019

[8] J. Bailey-Serres, J. E. Parker, E. A. Ainsworth, G. E. D. Oldroyd, and J. I. Schroeder, “Genetic strategies for improving crop yields,” *Nature*, vol. 575, no. 7781, pp. 109–118, 2019 31695205 10.1038/s41586-019-1679-0 PMC7024682

[9] FAO *Food and Agriculture Organization of the United Nations, Rome, 2018*, p. 60, 2018

[10] S. S. Myers, M. R. Smith, S. Guth, C. D. Golden, B. Vaitla, N. D. Mueller, A. D. Dangour, and P. Huybers, “Climate change and global food systems: potential impacts on food security and undernutrition,” *Annual Review of Public Health*, vol. 38, no. 1, pp. 259–277, 2017 10.1146/annurev-publhealth-031816-044356 28125383

[11] G. A. Pavlopoulos, M. Secrier, C. N. Moschopoulos, T. G. Soldatos, S. Kossida, J. Aerts, R. Schneider, and P. G. Bagos, “Using graph theory to analyze biological networks,” *BioData Mining*, vol. 4, no. 1, 2011 10.1186/1756-0381-4-10 PMC3101653 21527005

[12] E. Watson, L. T. MacNeil, H. E. Arda, L. J. Zhu, and A. J. M. Walhout, “Integration of metabolic and gene regulatory networks modulates the *C. elegans* dietary response,” *Cell*, vol. 153, no. 1, pp. 253–266, 2013 23540702 10.1016/j.cell.2013.02.050 PMC3817025

[13] M. M. Rinschen, J. Ivanisevic, M. Giera, and G. Siuzdak, “Identification of bioactive metabolites using activity metabolomics,” *Nature Reviews. Molecular Cell Biology*, vol. 20, no. 6, pp. 353–367, 2019 30814649 10.1038/s41580-019-0108-4 PMC6613555

[14] H. He, F. Van Breusegem, and A. Mhamdi, “Redox-dependent control of nuclear transcription in plants,” *Journal of Experimental Botany*, vol. 69, no. 14, pp. 3359–3372, 2018 29659979 10.1093/jxb/ery130

[15] Y. Shen, E. Issakidis-Bourguet, and D.-X. Zhou, “Perspectives on the interactions between metabolism, redox, and epigenetics in plants,” *Journal of Experimental Botany*, vol. 67, no. 18, pp. 5291–5300, 2016 27531885 10.1093/jxb/erw310

[16] S. Duran-Nebreda, and G. W. Bassel, “Plant behaviour in response to the environment: information processing in the solid state,” *Philosophical Transactions of the Royal Society B: Biological Sciences*, vol. 374, no. 1774, article 20180370, 2019 10.1098/rstb.2018.0370 PMC6553596 31006360

[17] G. W. Bassel, “Information processing and distributed computation in plant organs,” *Trends in Plant Science*, vol. 23, no. 11, pp. 994–1005, 2018 30219546 10.1016/j.tplants.2018.08.006

[18] E. Wong, B. Baur, S. Quader, and C. H. Huang, “Biological network motif detection: principles and practice,” *Briefings in Bioinformatics*, vol. 13, no. 2, pp. 202–215, 2012 22396487 10.1093/bib/bbr033 PMC3294240

[19] T. E. Gorochowski, C. S. Grierson, and M. di Bernardo, “Organization of feed-forward loop motifs reveals architectural principles in natural and engineered networks,” *Science Advances*, vol. 4, no. 3, article eaap9751, 2018 10.1126/sciadv.aap9751 PMC5903899 29670941

[20] P. Gupta, and S. K. Singh *Molecular Approaches in Plant Biology and Environmental Challenges*, S. P. Singh, S. K. Upadhyay, A. Pandey, and S. Kumar, Eds., Springer Singapore, Singapore, 2019

[21] G. Jin *Encyclopedia of Systems Biology*, W. Dubitzky, O. Wolkenhauer, K.-H. Cho, and H. Yokota, Eds., Springer, New York, NY, 2013

[22] I. Piazza, K. Kochanowski, V. Cappelletti, T. Fuhrer, E. Noor, U. Sauer, and P. Picotti, “A map of protein-metabolite interactions reveals principles of chemical communication,” *Cell*, vol. 172, no. 1-2, pp. 358–372.e23, 2018 29307493 10.1016/j.cell.2017.12.006

[23] O. Erbilgin, O. Rübel, K. B. Louie, M. Trinh, M. . Raad, T. Wildish, D. Udwary, C. Hoover, S. Deutsch, T. R. Northen, and B. P. Bowen, “MAGI: a method for metabolite annotation and gene integration,” *ACS Chemical Biology*, vol. 14, no. 4, pp. 704–714, 2019 30896917 10.1021/acschembio.8b01107

[24] I. Thiele, and B. Ø. Palsson, “A protocol for generating a high-quality genome-scale metabolic reconstruction,” *Nature Protocols*, vol. 5, no. 1, pp. 93–121, 2010 20057383 10.1038/nprot.2009.203 PMC3125167

[25] S. K. Masakapalli, N. J. Kruger, and R. G. Ratcliffe, “The metabolic flux phenotype of heterotrophic *Arabidopsis* cells reveals a complex response to changes in nitrogen supply,” *Plant Journal*, vol. 74, no. 4, pp. 569–582, 2013 10.1111/tpj.12142 23406511

[26] T. C. R. Williams, L. J. Sweetlove, and R. G. Ratcliffe, “Capturing metabolite channeling in metabolic flux phenotypes,” *Plant Physiology*, vol. 157, no. 3, pp. 981–984, 2011 21896888 10.1104/pp.111.184887 PMC3252163

[27] D. K. Allen, “Quantifying plant phenotypes with isotopic labeling & metabolic flux analysis,” *Current Opinion in Biotechnology*, vol. 37, pp. 45–52, 2016 26613198 10.1016/j.copbio.2015.10.002

[28] M. G. Poolman, L. Miguet, L. J. Sweetlove, and D. A. Fell, “A genome-scale metabolic model of *Arabidopsis* and some of its properties,” *Plant Physiology*, vol. 151, no. 3, pp. 1570–1581, 2009 19755544 10.1104/pp.109.141267 PMC2773075

[29] C. G. de Oliveira Dal'Molin, L.-E. Quek, R. W. Palfreyman, S. M. Brumbley, and L. K. Nielsen, “AraGEM, a genome-scale reconstruction of the primary metabolic network in *Arabidopsis*,” *Plant Physiology*, vol. 152, no. 2, pp. 579–589, 2010 20044452 10.1104/pp.109.148817 PMC2815881

[30] T. J. Clark, L. Guo, J. Morgan, and J. Schwender, “Modeling plant metabolism: from network reconstruction to mechanistic models,” *Annual Review of Plant Biology*, vol. 71, no. 1, pp. 303–326, 2020 10.1146/annurev-arplant-050718-100221 32017600

[31] D. McCloskey, B. Ø. Palsson, and A. M. Feist, “Basic and applied uses of genome-scale metabolic network reconstructions of *Escherichia coli*,” *Molecular Systems Biology*, vol. 9, no. 1, p. 661, 2013 23632383 10.1038/msb.2013.18 PMC3658273

[32] M. A. Oberhardt, B. Ø. Palsson, and J. A. Papin, “Applications of genome-scale metabolic reconstructions,” *Molecular Systems Biology*, vol. 5, no. 1, p. 320, 2009 19888215 10.1038/msb.2009.77 PMC2795471

[33] N. D. Price, J. L. Reed, and B. O. Palsson, “Genome-scale models of microbial cells: evaluating the consequences of constraints,” *Nature Reviews Microbiology*, vol. 2, no. 11, pp. 886–897, 2004 15494745 10.1038/nrmicro1023

[34] C. T. Trinh, A. Wlaschin, and F. Srienc, “Elementary mode analysis: a useful metabolic pathway analysis tool for characterizing cellular metabolism,” *Applied Microbiology and Biotechnology*, vol. 81, no. 5, pp. 813–826, 2009 19015845 10.1007/s00253-008-1770-1 PMC2909134

[35] R. A. Notebaart, B. Szappanos, B. Kintses, F. Pal, A. Gyorkei, B. Bogos, V. Lazar, R. Spohn, B. Csorg, A. Wagner, E. Ruppin, C. Pal, and B. Papp, “Network-level architecture and the evolutionary potential of underground metabolism,” *Proceedings of the National Academy of Sciences*, vol. 111, no. 32, pp. 11762–11767, 2014 10.1073/pnas.1406102111 PMC4136603 25071190

[36] Y. Luo, A. Widmer, and S. Karrenberg, “The roles of genetic drift and natural selection in quantitative trait divergence along an altitudinal gradient in *Arabidopsis thaliana*,” *Heredity*, vol. 114, no. 2, pp. 220–228, 2015 25293874 10.1038/hdy.2014.89 PMC4815633

[37] M. Pigliucci, and G. Muller *Evolution–The Extended Synthesis*, The MIT Press, Cambridge, Massachusetts, 2010

[38] S. J. Gould, “The exaptive excellence of spandrels as a term and prototype,” *Proceedings of the National Academy of Sciences*, vol. 94, no. 20, pp. 10750–10755, 1997 10.1073/pnas.94.20.10750 PMC23474 11038582

[39] S. J. Gould, and R. C. Lewontin, “The spandrels of San Marco and the Panglossian paradigm: a critique of the adaptationist programme,” *Proceedings of the Royal Society B: Biological Sciences*, vol. 205, no. 1161, pp. 581–598, 1979 42062 10.1098/rspb.1979.0086

[40] M.-L. Weng, C. Becker, J. Hildebrandt, M. Neumann, M. T. Rutter, R. G. Shaw, D. Weigel, and C. B. Fenster, “Fine-grained analysis of spontaneous mutation spectrum and frequency in *Arabidopsis thaliana*,” *Genetics*, vol. 211, no. 2, pp. 703–714, 2019 30514707 10.1534/genetics.118.301721 PMC6366913

[41] L. Wang, Y. Ji, Y. Hu, H. Hu, X. Jia, M. Jiang, X. Zhang, L. Zhao, Y. Zhang, Y. Jia, C. Qin, L. Yu, J. Huang, S. Yang, L. D. Hurst, and D. Tian, “The architecture of intra-organism mutation rate variation in plants,” *PLoS Biology*, vol. 17, no. 4, pp. e3000191–e3000191, 2019 30964866 10.1371/journal.pbio.3000191 PMC6456163

[42] B. T. Hofmeister, J. Denkena, M. Colomé-Tatché, Y. Shahryary, R. Hazarika, J. Grimwood, S. Mamidi, J. Jenkins, P. P. Grabowski, A. Sreedasyam, S. Shu, K. Barry, K. Lail, C. Adam, A. Lipzen, R. Sorek, D. Kudrna, J. Talag, R. Wing, D. W. Hall, D. Jacobsen, G. A. Tuskan, J. Schmutz, F. Johannes, and R. J. Schmitz, “A genome assembly and the somatic genetic and epigenetic mutation rate in a wild long-lived perennial *Populus trichocarpa*,” *Genome Biology*, vol. 21, no. 1, p. 259, 2020 33023654 10.1186/s13059-020-02162-5 PMC7539514

[43] S. Ossowski, K. Schneeberger, J. I. Lucas-Lledo, N. Warthmann, R. M. Clark, R. G. Shaw, D. Weigel, and M. Lynch, “The rate and molecular spectrum of spontaneous mutations in *Arabidopsis thaliana*,” *Science*, vol. 327, no. 5961, pp. 92–94, 2009 10.1126/science.1180677 PMC3878865 20044577

[44] D. Auboeuf, “Physicochemical foundations of life that direct evolution: chance and natural selection are not evolutionary driving forces,” *Life*, vol. 10, no. 2, p. 7, 2020 31973071 10.3390/life10020007 PMC7175370

[45] S. Garcia, and C. T. Trinh, “Modular design: implementing proven engineering principles in biotechnology,” *Biotechnology Advances*, vol. 37, no. 7, article 107403, 2019 10.1016/j.biotechadv.2019.06.002 31181317

[46] T. D. Miller, and P. Elgard *Proceedings of the 13th IPS Research Seminar, Fuglsoe*, Aalborg Universiy Fuglsoe, 1998

[47] J.-D. J. Han, N. Bertin, T. Hao, D. S. Goldberg, G. F. Berriz, L. V. Zhang, D. Dupuy, A. J. M. Walhout, M. E. Cusick, F. P. Roth, and M. Vidal, “Evidence for dynamically organized modularity in the yeast protein–protein interaction network,” *Nature*, vol. 430, no. 6995, pp. 88–93, 2004 15190252 10.1038/nature02555

[48] M. E. J. Newman, “Modularity and community structure in networks,” *Proceedings of the National Academy of Sciences*, vol. 103, no. 23, pp. 8577–8582, 2006 10.1073/pnas.0601602103 PMC1482622 16723398

[49] E. Ravasz, A. L. Somera, D. A. Mongru, Z. N. Oltvai, and A.-L. Barabási, “Hierarchical organization of modularity in metabolic networks,” *Science*, vol. 297, no. 5586, pp. 1551–1555, 2002 12202830 10.1126/science.1073374

[50] G. P. Wagner, M. Pavlicev, and J. M. Cheverud, “The road to modularity,” *Nature Reviews Genetics*, vol. 8, no. 12, pp. 921–931, 2007 10.1038/nrg2267 18007649

[51] S. Garcia, and C. T. Trinh, “Harnessing natural modularity of metabolism with goal attainment optimization to design a modular chassis cell for production of diverse chemicals,” *ACS Synthetic Biology*, vol. 9, no. 7, pp. 1665–1681, 2020 32470305 10.1021/acssynbio.9b00518

[52] H. Aigner, R. H. Wilson, A. Bracher, L. Calisse, J. Y. Bhat, F. U. Hartl, and M. Hayer-Hartl, “Plant RuBisCo assembly in *E. coli* with five chloroplast chaperones including BSD2,” *Science*, vol. 358, no. 6368, pp. 1272–1278, 2017 29217567 10.1126/science.aap9221

[53] W. Guo, J. Sheng, and X. Feng, “Mini-review: in vitro metabolic engineering for biomanufacturing of high-value products,” *Computational and Structural Biotechnology Journal*, vol. 15, pp. 161–167, 2017 28179978 10.1016/j.csbj.2017.01.006 PMC5288458

[54] S. Garcia, and C. T. Trinh, “Multiobjective strain design: a framework for modular cell engineering,” *Metabolic Engineering*, vol. 51, pp. 110–120, 2019 30201314 10.1016/j.ymben.2018.09.003

[55] S. Garcia, and C. T. Trinh, “Comparison of multi-objective evolutionary algorithms to solve the modular cell design problem for novel biocatalysis,” *Processes*, vol. 7, no. 6, p. 361, 2019

[56] K. Kaufmann, A. Pajoro, and G. C. Angenent, “Regulation of transcription in plants: mechanisms controlling developmental switches,” *Nature Reviews Genetics*, vol. 11, no. 12, pp. 830–842, 2010 10.1038/nrg2885 21063441

[57] J. Xiao, R. Jin, and D. Wagner, “Developmental transitions: integrating environmental cues with hormonal signaling in the chromatin landscape in plants,” *Genome Biology*, vol. 18, no. 1, p. 88, 2017 28490341 10.1186/s13059-017-1228-9 PMC5425979

[58] D. Chen, W. Yan, L.-Y. Fu, and K. Kaufmann, “Architecture of gene regulatory networks controlling flower development in *Arabidopsis thaliana*,” *Nature Communications*, vol. 9, no. 1, article 4534, 2018 10.1038/s41467-018-06772-3 PMC6208445 30382087

[59] Y. Liu, F. Guérard, M. Hodges, and M. Jossier, “Phosphomimetic T335D mutation of hydroxypyruvate reductase 1 modifies cofactor specificity and impacts *Arabidopsis* growth in air,” *Plant Physiology*, vol. 183, no. 1, pp. 194–205, 2020 32156771 10.1104/pp.19.01225 PMC7210656

[60] J. Anderson, N. Strelkowa, G. B. Stan, T. Douglas, J. Savulescu, M. Barahona, and A. Papachristodoulou, “Engineering and ethical perspectives in synthetic biology. Rigorous, robust and predictable designs, public engagement and a modern ethical framework are vital to the continued success of synthetic biology,” *EMBO Reports*, vol. 13, no. 7, pp. 584–590, 2012 22699939 10.1038/embor.2012.81 PMC3389334

[61] D. L. Trudeau, C. Edlich-Muth, J. Zarzycki, M. Scheffen, M. Goldsmith, O. Khersonsky, Z. Avizemer, S. J. Fleishman, C. A. R. Cotton, T. J. Erb, D. S. Tawfik, and A. Bar-Even, “Design and in vitro realization of carbon-conserving photorespiration,” *Proceedings of the National Academy of Sciences*, vol. 115, no. 49, pp. E11455–E11464, 2018 10.1073/pnas.1812605115 PMC6298115 30459276

[62] J. Dalal, H. Lopez, N. B. Vasani, Z. Hu, J. E. Swift, R. Yalamanchili, M. Dvora, X. Lin, D. Xie, R. Qu, and H. W. Sederoff, “A photorespiratory bypass increases plant growth and seed yield in biofuel crop *Camelina sativa*,” *Biotechnology for Biofuels*, vol. 8, no. 1, article 175, 2015 10.1186/s13068-015-0357-1 PMC4625952 26516348

[63] J. M. Whitacre, and A. Bender, “Networked buffering: a basic mechanism for distributed robustness in complex adaptive systems,” *Theoretical Biology & Medical Modelling*, vol. 7, no. 1, pp. 20–20, 2010 20550663 10.1186/1742-4682-7-20 PMC2901314

[64] S. Yamada, T. Yamada, S. Bracke, and M. Inoue, “Upgradable design for reduction of production cost and CO_2_ emission - case study of a laptop computer,” *Applied Mechanics and Materials*, vol. 761, pp. 589–593, 2015

[65] H. M, K. E, W. E, and R. B, “Simple and versatile selection of *Arabidopsis* transformants,” *Plant Cell Reports*, vol. 21, no. 2, pp. 130–135, 2002

[66] L. Hu, H. Li, R. Qin, R. Xu, J. Li, L. Li, P. Wei, and J. Yang, “Plant phosphomannose isomerase as a selectable marker for rice transformation,” *Scientific Reports*, vol. 6, no. 1, article 25921, 2016 10.1038/srep25921 PMC4865823 27174847

[67] Y.-C. Kuan, V. Thiruvengadam, J. S. Lin, J. H. Liu, T. J. Chen, H. M. Wu, W. C. Wang, and L. J. Chen, “Broad-specificity amino acid racemase, a novel non-antibiotic selectable marker for transgenic plants,” *Plant Biotechnology Reports*, vol. 12, no. 1, pp. 27–38, 2018

[68] D. P. Chin, I. Shiratori, A. Shimizu, K. Kato, M. Mii, and I. Waga, “Generation of brilliant green fluorescent petunia plants by using a new and potent fluorescent protein transgene,” *Scientific Reports*, vol. 8, no. 1, article 16556, 2018 10.1038/s41598-018-34837-2 PMC6224394 30410086

[69] S. Yang, Y. Hu, Z. Cheng, J. H. Rice, L. Miao, J. Ma, T. Hewezi, Y. Li, and J. Gai, “An efficient *Agrobacterium*-mediated soybean transformation method using green fluorescent protein as a selectable marker,” *Plant Signaling & Behavior*, vol. 14, no. 7, article 1612682, 2019 10.1080/15592324.2019.1612682 PMC6619996 31056001

[70] Z. Polóniová, M. Jopčík, I. Matušíková, J. Libantová, and J. Moravčíková, “Preparation of plant transformation vector containing “Self-excision” Cre/loxP system,” *Journal of Microbiology, Biotechnology and Food Sciences*, vol. 2019, pp. 563–572, 2019

[71] D. Du, R. Jin, J. Guo, and F. Zhang, “Construction of marker-free genetically modified maize using a heat-inducible auto-excision vector,” *Genes*, vol. 10, no. 5, p. 374, 2019 31108922 10.3390/genes10050374 PMC6562874

[72] J. P. Cody, N. D. Graham, C. Zhao, N. C. Swyers, and J. A. Birchler, “Site-specific recombinase genome engineering toolkit in maize,” *Plant Direct*, vol. 4, article e00209, 2020 10.1002/pld3.209 PMC7061458 32166212

[73] Y. Wang, Y.-Y. Yau, D. Perkins-Balding, and J. G. Thomson, “Recombinase technology: applications and possibilities,” *Plant Cell Reports*, vol. 30, no. 3, pp. 267–285, 2011 20972794 10.1007/s00299-010-0938-1 PMC3036822

[74] B. P. Pathak, E. Pruett, H. Guan, and V. Srivastava, “Utility of I-SceI and CCR5-ZFN nucleases in excising selectable marker genes from transgenic plants,” *BMC Research Notes*, vol. 12, no. 1, article 272, 2019 10.1186/s13104-019-4304-2 PMC6518718 31088537

[75] R. K. Singh, L. Sharma, N. Bohra, S. Anandhan, E. Ruiz-May, and F. R. Quiroz-Figueroa *Advances in Plant Transgenics: Methods and Applications*, R. Sathishkumar, S. R. Kumar, J. Hema, and V. Baskar, Eds., Springer Singapore, Singapore, 2019

[76] V. Srivastava, J. L. Underwood, and S. Zhao, “Dual-targeting by CRISPR/Cas9 for precise excision of transgenes from rice genome,” *Plant Cell, Tissue and Organ Culture*, vol. 129, no. 1, pp. 153–160, 2017

[77] S. Galanie, K. Thodey, I. J. Trenchard, M. Filsinger Interrante, and C. D. Smolke, “Complete biosynthesis of opioids in yeast,” *Science*, vol. 349, no. 6252, pp. 1095–1100, 2015 26272907 10.1126/science.aac9373 PMC4924617

[78] P. Opgenorth, Z. Costello, T. Okada, G. Goyal, Y. Chen, J. Gin, V. Benites, M. de Raad, T. R. Northen, K. Deng, S. Deutsch, E. E. K. Baidoo, C. J. Petzold, N. J. Hillson, H. Garcia Martin, and H. R. Beller, “Lessons from two design–build–test–learn cycles of dodecanol production in *Escherichia coli* aided by machine learning,” *ACS Synthetic Biology*, vol. 8, no. 6, pp. 1337–1351, 2019 31072100 10.1021/acssynbio.9b00020

[79] J. G. Perez, J. C. Stark, and M. C. Jewett, “Cell-free synthetic biology: engineering beyond the cell,” *Cold Spring Harbor Perspectives in Biology*, vol. 8, p. a023853, 2016 27742731 10.1101/cshperspect.a023853 PMC5131772

[80] E. Oberortner, J.-F. Cheng, N. J. Hillson, and S. Deutsch, “Streamlining the design-to-build transition with build-optimization software tools,” *ACS Synthetic Biology*, vol. 6, pp. 485–496, 2016 28004921 10.1021/acssynbio.6b00200

[81] Y. Chen, J. M. Guenther, J. W. Gin, L. J. G. Chan, Z. Costello, T. L. Ogorzalek, H. M. Tran, J. M. Blake-Hedges, J. D. Keasling, P. D. Adams, H. García Martín, N. J. Hillson, and C. J. Petzold, “Automated “Cells-to-peptides” sample preparation workflow for high-throughput, quantitative proteomic assays of microbes,” *Journal of Proteome Research*, vol. 18, no. 10, pp. 3752–3761, 2019 31436101 10.1021/acs.jproteome.9b00455

[82] J. Zhang, S. D. Petersen, T. Radivojevic, A. Ramirez, A. Pérez-Manríquez, E. Abeliuk, B. J. Sánchez, Z. Costello, Y. Chen, M. J. Fero, H. G. Martin, J. Nielsen, J. D. Keasling, and M. K. Jensen, “Combining mechanistic and machine learning models for predictive engineering and optimization of tryptophan metabolism,” *Nature Communications*, vol. 11, no. 1, pp. 4880–4880, 2020 10.1038/s41467-020-17910-1 PMC7519671 32978375

[83] R. Chao, S. Mishra, T. Si, and H. Zhao, “Engineering biological systems using automated biofoundries,” *Metabolic Engineering*, vol. 42, pp. 98–108, 2017 28602523 10.1016/j.ymben.2017.06.003 PMC5544601

[84] T. S. Ham, Z. Dmytriv, H. Plahar, J. Chen, N. J. Hillson, and J. D. Keasling, “Design, implementation and practice of JBEI-ICE: an open source biological part registry platform and tools,” *Nucleic Acids Research*, vol. 40, no. 18, p. e141, 2012 22718978 10.1093/nar/gks531 PMC3467034

[85] W. C. Morrell, G. W. Birkel, M. Forrer, T. Lopez, T. W. H. Backman, M. Dussault, C. J. Petzold, E. E. K. Baidoo, Z. Costello, D. Ando, J. Alonso-Gutierrez, K. W. George, A. Mukhopadhyay, I. Vaino, J. D. Keasling, P. D. Adams, N. J. Hillson, and H. Garcia Martin, “The experiment data depot: a web-based software tool for biological experimental data storage, sharing, and visualization,” *ACS Synthetic Biology*, vol. 6, no. 12, pp. 2248–2259, 2017 28826210 10.1021/acssynbio.7b00204

[86] B. Wilbanks, D. S. Layton, S. Garcia, and C. T. Trinh, “A prototype for modular cell engineering,” *ACS Synthetic Biology*, vol. 7, pp. 187–199, 2017 29017319 10.1021/acssynbio.7b00269

[87] C. T. Trinh, Y. Liu, and D. Conner, “Rational design of efficient modular cells,” *Metabolic Engineering*, vol. 32, pp. 220–231, 2015 26497627 10.1016/j.ymben.2015.10.005

[88] B. Canton, A. Labno, and D. Endy, “Refinement and standardization of synthetic biological parts and devices,” *Nature Biotechnology*, vol. 26, no. 7, pp. 787–793, 2008 10.1038/nbt1413 18612302

[89] N. J. Patron, D. Orzaez, S. Marillonnet, H. Warzecha, C. Matthewman, M. Youles, O. Raitskin, A. Leveau, G. Farré, C. Rogers, A. Smith, J. Hibberd, A. A. R. Webb, J. Locke, S. Schornack, J. Ajioka, D. C. Baulcombe, C. Zipfel, S. Kamoun, J. D. G. Jones, H. Kuhn, S. Robatzek, H. P. van Esse, D. Sanders, G. Oldroyd, C. Martin, R. Field, S. O'Connor, S. Fox, B. Wulff, B. Miller, A. Breakspear, G. Radhakrishnan, P. M. Delaux, D. Loqué, A. Granell, A. Tissier, P. Shih, T. P. Brutnell, W. P. Quick, H. Rischer, P. D. Fraser, A. Aharoni, C. Raines, P. F. South, J. M. Ané, B. R. Hamberger, J. Langdale, J. Stougaard, H. Bouwmeester, M. Udvardi, J. A. H. Murray, V. Ntoukakis, P. Schäfer, K. Denby, K. J. Edwards, A. Osbourn, and J. Haseloff, “Standards for plant synthetic biology: a common syntax for exchange of DNA parts,” *New Phytologist*, vol. 208, no. 1, pp. 13–19, 2015 26171760 10.1111/nph.13532

[90] C. D. Smolke, “Building outside of the box: iGEM and the BioBricks Foundation,” *Nature Biotechnology*, vol. 27, no. 12, pp. 1099–1102, 2009 10.1038/nbt1209-1099 20010584

[91] C. Goodman, “Engineering ingenuity at iGEM,” *Nature Chemical Biology*, vol. 4, no. 1, pp. 13–13, 2008 18084272 10.1038/nchembio0108-13

[92] J. A. McLaughlin, C. J. Myers, Z. Zundel, G. Mısırlı, M. Zhang, I. D. Ofiteru, A. Goñi-Moreno, and A. Wipat, “SynBioHub: a standards-enabled design repository for synthetic biology,” *ACS Synthetic Biology*, vol. 7, no. 2, pp. 682–688, 2018 29316788 10.1021/acssynbio.7b00403

[93] J. Kamens, “The Addgene repository: an international nonprofit plasmid and data resource,” *Nucleic Acids Research*, vol. 43, pp. D1152–D1157, 2014 25392412 10.1093/nar/gku893 PMC4384007

[94] M. Herscovitch, E. Perkins, A. Baltus, and M. Fan, “Addgene provides an open forum for plasmid sharing,” *Nature Biotechnology*, vol. 30, no. 4, pp. 316–317, 2012 10.1038/nbt.2177 22491276

[95] S. Werner, C. Engler, E. Weber, R. Gruetzner, and S. Marillonnet, “Fast track assembly of multigene constructs using Golden Gate cloning and the MoClo system,” *Bioengineered*, vol. 3, pp. 38–43, 2014 10.4161/bbug.3.1.18223 22126803

[96] C. Engler, M. Youles, R. Gruetzner, T. M. Ehnert, S. Werner, J. D. G. Jones, N. J. Patron, and S. Marillonnet, “A Golden Gate modular cloning toolbox for plants,” *ACS Synthetic Biology*, vol. 3, no. 11, pp. 839–843, 2014 24933124 10.1021/sb4001504

[97] A. Occhialini, A. A. Piatek, A. C. Pfotenhauer, T. P. Frazier, C. N. Stewart Jr., and S. C. Lenaghan, “MoChlo: a versatile, modular cloning toolbox for chloroplast biotechnology,” *Plant Physiology*, vol. 179, no. 3, pp. 943–957, 2019 30679266 10.1104/pp.18.01220 PMC6393787

[98] S. G. Hussey, J. Grima-Pettenati, A. A. Myburg, E. Mizrachi, S. M. Brady, Y. Yoshikuni, and S. Deutsch, “A standardized synthetic *Eucalyptus* transcription factor and promoter panel for re-engineering secondary cell wall regulation in biomass and bioenergy crops,” *ACS Synthetic Biology*, vol. 8, no. 2, pp. 463–465, 2019 30605615 10.1021/acssynbio.8b00440

[99] M. S. Belcher, K. M. Vuu, A. Zhou, N. Mansoori, A. Agosto Ramos, M. G. Thompson, H. V. Scheller, D. Loqué, and P. M. Shih, “Design of orthogonal regulatory systems for modulating gene expression in plants,” *Nature Chemical Biology*, vol. 16, no. 8, pp. 857–865, 2020 32424304 10.1038/s41589-020-0547-4

[100] J. Kuo, F. Stirling, Y. H. Lau, Y. Shulgina, J. C. Way, and P. A. Silver, “Synthetic genome recoding: new genetic codes for new features,” *Current Genetics*, vol. 64, no. 2, pp. 327–333, 2018 28983660 10.1007/s00294-017-0754-z PMC5849531

[101] A. P. Arkin, R. W. Cottingham, C. S. Henry, N. L. Harris, R. L. Stevens, S. Maslov, P. Dehal, D. Ware, F. Perez, S. Canon, M. W. Sneddon, M. L. Henderson, W. J. Riehl, D. Murphy-Olson, S. Y. Chan, R. T. Kamimura, S. Kumari, M. M. Drake, T. S. Brettin, E. M. Glass, D. Chivian, D. Gunter, D. J. Weston, B. H. Allen, J. Baumohl, A. A. Best, B. Bowen, S. E. Brenner, C. C. Bun, J. M. Chandonia, J. M. Chia, R. Colasanti, N. Conrad, J. J. Davis, B. H. Davison, M. DeJongh, S. Devoid, E. Dietrich, I. Dubchak, J. N. Edirisinghe, G. Fang, J. P. Faria, P. M. Frybarger, W. Gerlach, M. Gerstein, A. Greiner, J. Gurtowski, H. L. Haun, F. He, R. Jain, M. P. Joachimiak, K. P. Keegan, S. Kondo, V. Kumar, M. L. Land, F. Meyer, M. Mills, P. S. Novichkov, T. Oh, G. J. Olsen, R. Olson, B. Parrello, S. Pasternak, E. Pearson, S. S. Poon, G. A. Price, S. Ramakrishnan, P. Ranjan, P. C. Ronald, M. C. Schatz, S. M. D. Seaver, M. Shukla, R. A. Sutormin, M. H. Syed, J. Thomason, N. L. Tintle, D. Wang, F. Xia, H. Yoo, S. Yoo, and D. Yu, “KBase: The United States Department of Energy Systems Biology Knowledgebase,” *Nature Biotechnology*, vol. 36, no. 7, pp. 566–569, 2018 10.1038/nbt.4163 PMC6870991 29979655

[102] D. Liu, R. Hu, K. J. Palla, G. A. Tuskan, and X. Yang, “Advances and perspectives on the use of CRISPR/Cas9 systems in plant genomics research,” *Current Opinion in Plant Biology*, vol. 30, pp. 70–77, 2016 26896588 10.1016/j.pbi.2016.01.007

[103] A. V. Anzalone, P. B. Randolph, J. R. Davis, A. A. Sousa, L. W. Koblan, J. M. Levy, P. J. Chen, C. Wilson, G. A. Newby, A. Raguram, and D. R. Liu, “Search-and-replace genome editing without double-strand breaks or donor DNA,” *Nature*, vol. 576, no. 7785, pp. 149–157, 2019 31634902 10.1038/s41586-019-1711-4 PMC6907074

[104] J. L. Doman, A. Raguram, G. A. Newby, and D. R. Liu, “Evaluation and minimization of Cas9-independent off-target DNA editing by cytosine base editors,” *Nature Biotechnology*, vol. 38, no. 5, pp. 620–628, 2020 10.1038/s41587-020-0414-6 PMC7335424 32042165

[105] N. M. Gaudelli, A. C. Komor, H. A. Rees, M. S. Packer, A. H. Badran, D. I. Bryson, and D. R. Liu, “Programmable base editing of A•T to G•C in genomic DNA without DNA cleavage,” *Nature*, vol. 551, no. 7681, pp. 464–471, 2017 29160308 10.1038/nature24644 PMC5726555

[106] M. M. Hassan, G. Yuan, J.-G. Chen, G. A. Tuskan, and X. Yang, “Prime editing technology and its prospects for future applications in plant biology research,” *BioDesign Research*, vol. 2020, article 9350905, pp. 1–14, 2020 10.34133/2020/9350905 PMC10530660 37849904

[107] S. S. Bharat, S. Li, J. Li, L. Yan, and L. Xia, “Base editing in plants: current status and challenges,” *Crop Journal*, vol. 8, no. 3, pp. 384–395, 2020

[108] R. Mishra, R. K. Joshi, and K. Zhao, “Base editing in crops: current advances, limitations and future implications,” *Plant Biotechnology Journal*, vol. 18, pp. 20–31, 2019 31365173 10.1111/pbi.13225 PMC6920333

[109] R. Xu, J. Li, X. Liu, T. Shan, R. Qin, and P. Wei, “Development of plant prime-editing systems for precise genome editing,” *Plant Communications*, vol. 1, no. 3, article 100043, 2020 10.1016/j.xplc.2020.100043 PMC7747961 33367239

[110] Q. Lin, Y. Zong, C. Xue, S. Wang, S. Jin, Z. Zhu, Y. Wang, A. V. Anzalone, A. Raguram, J. L. Doman, D. R. Liu, and C. Gao, “Prime genome editing in rice and wheat,” *Nature Biotechnology*, vol. 38, no. 5, pp. 582–585, 2020 10.1038/s41587-020-0455-x 32393904

[111] M. H. Larson, L. A. Gilbert, X. Wang, W. A. Lim, J. S. Weissman, and L. S. Qi, “CRISPR interference (CRISPRi) for sequence-specific control of gene expression,” *Nature Protocols*, vol. 8, no. 11, pp. 2180–2196, 2013 24136345 10.1038/nprot.2013.132 PMC3922765

[112] K. R. Sanson, R. E. Hanna, M. Hegde, K. F. Donovan, C. Strand, M. E. Sullender, E. W. Vaimberg, A. Goodale, D. E. Root, F. Piccioni, and J. G. Doench, “Optimized libraries for CRISPR-Cas9 genetic screens with multiple modalities,” *Nature Communications*, vol. 9, no. 1, article 5416, 2018 10.1038/s41467-018-07901-8 PMC6303322 30575746

[113] O. O. Abudayyeh, J. S. Gootenberg, B. Franklin, J. Koob, M. J. Kellner, A. Ladha, J. Joung, P. Kirchgatterer, D. B. T. Cox, and F. Zhang, “A cytosine deaminase for programmable single-base RNA editing,” *Science*, vol. 365, no. 6451, pp. 382–386, 2019 31296651 10.1126/science.aax7063 PMC6956565

[114] M. Jinek, K. Chylinski, I. Fonfara, M. Hauer, J. A. Doudna, and E. Charpentier, “A programmable dual-RNA-guided DNA endonuclease in adaptive bacterial immunity,” *Science*, vol. 337, no. 6096, pp. 816–821, 2012 22745249 10.1126/science.1225829 PMC6286148

[115] A. A. K. Nielsen, B. S. der, J. Shin, P. Vaidyanathan, V. Paralanov, E. A. Strychalski, D. Ross, D. Densmore, and C. A. Voigt, “Genetic circuit design automation,” *Science*, vol. 352, no. 6281, article aac7341, 2016 10.1126/science.aac7341 27034378

[116] M. Zhang, J. A. McLaughlin, A. Wipat, and C. J. Myers, “SBOLDesigner 2: an intuitive tool for structural genetic design,” *ACS Synthetic Biology*, vol. 6, no. 7, pp. 1150–1160, 2017 28441476 10.1021/acssynbio.6b00275

[117] A. J. Walhout, “Protein interaction mapping in *C. elegans* using proteins involved in vulval development,” *Science*, vol. 287, no. 5450, pp. 116–122, 2000 10615043 10.1126/science.287.5450.116

[118] C. Engler, R. Kandzia, and S. Marillonnet, “A one pot, one step, precision cloning method with high throughput capability,” *PLoS One*, vol. 3, no. 11, pp. e3647–e3647, 2008 18985154 10.1371/journal.pone.0003647 PMC2574415

[119] D. G. Gibson, L. Young, R. Y. Chuang, J. C. Venter, C. A. Hutchison III, and H. O. Smith, “Enzymatic assembly of DNA molecules up to several hundred kilobases,” *Nature Methods*, vol. 6, no. 5, pp. 343–345, 2009 19363495 10.1038/nmeth.1318

[120] A. Sarrion-Perdigones, M. Vazquez-Vilar, J. Palaci, B. Castelijns, J. Forment, P. Ziarsolo, J. Blanca, A. Granell, and D. Orzaez, “GoldenBraid 2.0: a comprehensive DNA assembly framework for plant synthetic biology,” *Plant Physiology*, vol. 162, no. 3, pp. 1618–1631, 2013 23669743 10.1104/pp.113.217661 PMC3707536

[121] R.-B. Yang, L.-J. Bi, and X.-E. Zhang, “A novel T-type overhangs improve the enzyme-free cloning of PCR products,” *Molecular Biotechnology*, vol. 55, no. 1, pp. 10–16, 2013 23733332 10.1007/s12033-012-9597-5

[122] H. C. De Paoli, G. A. Tuskan, and X. Yang, “An innovative platform for quick and flexible joining of assorted DNA fragments,” *Scientific Reports*, vol. 6, no. 1, article 19278, 2016 10.1038/srep19278 PMC4725820 26758940

[123] C. Lei, S.-Y. Li, J.-K. Liu, X. Zheng, G.-P. Zhao, and J. Wang, “The CCTL (Cpf1-assisted cutting and Taq DNA ligase-assisted ligation) method for efficient editing of large DNA constructs in vitro,” *Nucleic Acids Research*, vol. 45, pp. e74–e74, 2017 28115632 10.1093/nar/gkx018 PMC5436000

[124] S. B. Moon, J. M. Lee, J. G. Kang, N.-E. Lee, D.-I. Ha, D. Y. Kim, S. H. Kim, K. Yoo, D. Kim, J.-H. Ko, and Y.-S. Kim, “Highly efficient genome editing by CRISPR-Cpf1 using CRISPR RNA with a uridinylate-rich 3’-overhang,” *Nature Communications*, vol. 9, no. 1, article 3651, 2018 10.1038/s41467-018-06129-w PMC6128929 30194297

[125] R. T. Fuchs, J. Curcuru, M. Mabuchi, P. Yourik, and G. B. Robb, “Cas12a trans-cleavage can be modulated in vitro and is active on ssDNA, dsDNA, and RNA,” bioRxiv, 2019

[126] B. Pollak, T. Matute, I. Nuñez, A. Cerda, C. Lopez, V. Vargas, A. Kan, V. Bielinski, P. von Dassow, C. L. Dupont, and F. Federici, “Universal loop assembly: open, efficient and cross-kingdom DNA fabrication,” *Synthetic Biology*, vol. 5, no. 1, article ysaa001, 2020 10.1093/synbio/ysaa001 PMC7052795 32161816

[127] V. T. Dien, M. Holcomb, A. W. Feldman, E. C. Fischer, T. J. Dwyer, and F. E. Romesberg, “Progress toward a semi-synthetic organism with an unrestricted expanded genetic alphabet,” *Journal of the American Chemical Society*, vol. 140, no. 47, pp. 16115–16123, 2018 30418780 10.1021/jacs.8b08416 PMC6373772

[128] J. C. Braman, and P. J. Sheffield, “Seamless assembly of DNA parts into functional devices and higher order multi-device systems,” *PLoS One*, vol. 14, no. 6, article e0199653, 2019 10.1371/journal.pone.0199653 PMC6599225 31251741

[129] P. M. Shih, K. Vuu, N. Mansoori, L. Ayad, K. B. Louie, B. P. Bowen, T. R. Northen, and D. Loqué, “A robust gene-stacking method utilizing yeast assembly for plant synthetic biology,” *Nature Communications*, vol. 7, no. 1, article 13215, 2016 10.1038/ncomms13215 PMC5095168 27782150

[130] J. E. Venetz, L. del Medico, A. Wölfle, P. Schächle, Y. Bucher, D. Appert, F. Tschan, C. E. Flores-Tinoco, M. van Kooten, R. Guennoun, S. Deutsch, M. Christen, and B. Christen, “Chemical synthesis rewriting of a bacterial genome to achieve design flexibility and biological functionality,” *Proceedings of the National Academy of Sciences*, vol. 116, no. 16, pp. 8070–8079, 2019 10.1073/pnas.1818259116 PMC6475421 30936302

[131] J. Fredens, K. Wang, D. de la Torre, L. F. H. Funke, W. E. Robertson, Y. Christova, T. Chia, W. H. Schmied, D. L. Dunkelmann, V. Beránek, C. Uttamapinant, A. G. Llamazares, T. S. Elliott, and J. W. Chin, “Total synthesis of *Escherichia coli* with a recoded genome,” *Nature*, vol. 569, no. 7757, pp. 514–518, 2019 31092918 10.1038/s41586-019-1192-5 PMC7039709

[132] A. Ferrer, M. Arró, D. Manzano, and T. Altabella *Advanced Technologies for Protein Complex Production and Characterization*, M. C. Vega, Ed., Springer International Publishing, Cham, 2016

[133] B. M. O'Neill, K. L. Mikkelson, N. M. Gutierrez, J. L. Cunningham, K. L. Wolff, S. J. Szyjka, C. B. Yohn, K. E. Redding, and M. J. Mendez, “An exogenous chloroplast genome for complex sequence manipulation in algae,” *Nucleic Acids Research*, vol. 40, pp. 2782–2792, 2011 22116061 10.1093/nar/gkr1008 PMC3315318

[134] H. S. Schindel, A. A. Piatek, C. N. Stewart, and S. C. Lenaghan, “The plastid genome as a chassis for synthetic biology-enabled metabolic engineering: players in gene expression,” *Plant Cell Reports*, vol. 37, no. 10, pp. 1419–1429, 2018 30039465 10.1007/s00299-018-2323-4

[135] K. Lowe, E. Wu, N. Wang, G. Hoerster, C. Hastings, M. J. Cho, C. Scelonge, B. Lenderts, M. Chamberlin, J. Cushatt, L. Wang, L. Ryan, T. Khan, J. Chow-Yiu, W. Hua, M. Yu, J. Banh, Z. Bao, K. Brink, E. Igo, B. Rudrappa, P. M. Shamseer, W. Bruce, L. Newman, B. Shen, P. Zheng, D. Bidney, C. Falco, J. Register, Z. Y. Zhao, D. Xu, T. Jones, and W. Gordon-Kamm, “Morphogenic regulators *Baby boom* and *Wuschel* improve monocot transformation,” *Plant Cell*, vol. 28, no. 9, pp. 1998–2015, 2016 27600536 10.1105/tpc.16.00124 PMC5059793

[136] M. F. Maher, R. A. Nasti, M. Vollbrecht, C. G. Starker, M. D. Clark, and D. F. Voytas, “Plant gene editing through de novo induction of meristems,” *Nature Biotechnology*, vol. 38, no. 1, pp. 84–89, 2020 10.1038/s41587-019-0337-2 PMC6954279 31844292

[137] A. Plasencia, M. Soler, A. Dupas, N. Ladouce, G. Silva-Martins, Y. Martinez, C. Lapierre, C. Franche, I. Truchet, and J. Grima-Pettenati, “*Eucalyptus* hairy roots, a fast, efficient and versatile tool to explore function and expression of genes involved in wood formation,” *Plant Biotechnology Journal*, vol. 14, no. 6, pp. 1381–1393, 2016 26579999 10.1111/pbi.12502 PMC11388834

[138] Y. Dai, G. Hu, A. Dupas, L. Medina, N. Blandels, H. San Clemente, N. Ladouce, M. Badawi, G. Hernandez-Raquet, F. Mounet, J. Grima-Pettenati, and H. Cassan-Wang, “Implementing the CRISPR/Cas9 technology in *Eucalyptus* hairy roots using wood-related genes,” *International Journal of Molecular Sciences*, vol. 21, no. 10, p. 3408, 2020 32408486 10.3390/ijms21103408 PMC7279396

[139] R. P. Kaur, and S. Devi, “*In planta* transformation in plants: a review,” *Agricultural Reviews*, vol. 40, pp. 159–174, 2019

[140] Z. Bahari, S. Sazegari, A. Niazi, and A. Afsharifar, “The application of an *Agrobacterium*-mediated in planta transformation system in a *Catharanthus roseus* medicinal plant,” *Czech Journal of Genetics and Plant Breeding*, vol. 56, pp. 34–41, 2020

[141] N. E. Zlobin, M. V. Lebedeva, and V. V. Taranov, “CRISPR/Cas9 genome editing throughin plantatransformation,” *Critical Reviews in Biotechnology*, vol. 40, no. 2, pp. 153–168, 2020 31903793 10.1080/07388551.2019.1709795

[142] E. Toda, N. Koiso, A. Takebayashi, M. Ichikawa, T. Kiba, K. Osakabe, Y. Osakabe, H. Sakakibara, N. Kato, and T. Okamoto, “An efficient DNA- and selectable-marker-free genome-editing system using zygotes in rice,” *Nature Plants*, vol. 5, no. 4, pp. 363–368, 2019 30911123 10.1038/s41477-019-0386-z

[143] G. S. Demirer, H. Zhang, N. S. Goh, E. González-Grandío, and M. P. Landry, “Carbon nanotube–mediated DNA delivery without transgene integration in intact plants,” *Nature Protocols*, vol. 14, no. 10, pp. 2954–2971, 2019 31534231 10.1038/s41596-019-0208-9 PMC10496593

[144] Y. He, and Y. Zhao, “Technological breakthroughs in generating transgene-free and genetically stable CRISPR-edited plants,” *aBIOTECH*, vol. 1, no. 1, pp. 88–96, 2020 36305007 10.1007/s42994-019-00013-x PMC9584093

[145] A. Untergasser, G. J. M. Bijl, W. Liu, T. Bisseling, J. G. Schaart, and R. Geurts, “One-step *Agrobacterium* mediated transformation of eight genes essential for rhizobium symbiotic signaling using the novel binary vector system pHUGE,” *PLoS One*, vol. 7, no. 10, pp. e47885–e47885, 2012 23112864 10.1371/journal.pone.0047885 PMC3480454

[146] F. Pasin, L. C. Bedoya, J. M. Bernabé-Orts, A. Gallo, C. Simón-Mateo, D. Orzaez, and J. A. García, “Multiple T-DNA delivery to plants using novel mini binary vectors with compatible replication origins,” *ACS Synthetic Biology*, vol. 6, no. 10, pp. 1962–1968, 2017 28657330 10.1021/acssynbio.6b00354

[147] V. Srivastava *Transgenic Plants. Methods in Molecular Biology*, S. Kumar, P. Barone, and M. Smith, Eds., Humana Press, New York, NY, vol. vol 1864, 2019

[148] S. Jin, and H. Daniell, “The engineered chloroplast genome just got smarter,” *Trends in Plant Science*, vol. 20, no. 10, pp. 622–640, 2015 26440432 10.1016/j.tplants.2015.07.004 PMC4606472

[149] J. Yang, X. Xie, N. Xiang, Z. X. Tian, R. Dixon, and Y. P. Wang, “Polyprotein strategy for stoichiometric assembly of nitrogen fixation components for synthetic biology,” *Proceedings of the National Academy of Sciences*, vol. 115, no. 36, pp. E8509–E8517, 2018 10.1073/pnas.1804992115 PMC6130400 30061389

[150] V. A. Márquez-Escobar, O. González-Ortega, and S. Rosales-Mendoza *Prospects of Plant-Based Vaccines in Veterinary Medicine*, J. MacDonald, Ed., Springer International Publishing, Cham, 2018

[151] K. Lee, A. L. Eggenberger, R. Banakar, M. E. McCaw, H. Zhu, M. Main, M. Kang, S. B. Gelvin, and K. Wang, “CRISPR/Cas9-mediated targeted T-DNA integration in rice,” *Plant Molecular Biology*, vol. 99, no. 4-5, pp. 317–328, 2019 30645710 10.1007/s11103-018-00819-1

[152] D. Miki, W. Zhang, W. Zeng, Z. Feng, and J.-K. Zhu, “CRISPR/Cas9-mediated gene targeting in *Arabidopsis* using sequential transformation,” *Nature Communications*, vol. 9, no. 1, article 1967, 2018 10.1038/s41467-018-04416-0 PMC5958078 29773790

[153] M. Mellado-Sánchez, F. McDiarmid, V. Cardoso, K. Kanyuka, and D. R. MacGregor, “Virus-mediated transient expression techniques enable gene function studies in black-grass,” *Plant Physiology*, vol. 183, no. 2, pp. 455–459, 2020 32238443 10.1104/pp.20.00205 PMC7271810

[154] L. Jones, F. Ratcliff, and D. C. Baulcombe, “RNA-directed transcriptional gene silencing in plants can be inherited independently of the RNA trigger and requires Met1 for maintenance,” *Current Biology*, vol. 11, no. 10, pp. 747–757, 2001 11378384 10.1016/s0960-9822(01)00226-3

[155] Z. Ali, A. Abul-faraj, L. Li, N. Ghosh, M. Piatek, A. Mahjoub, M. Aouida, A. Piatek, N. J. Baltes, D. F. Voytas, S. Dinesh-Kumar, and M. M. Mahfouz, “Efficient virus-mediated genome editing in plants using the CRISPR/Cas9 system,” *Molecular Plant*, vol. 8, no. 8, pp. 1288–1291, 2015 25749112 10.1016/j.molp.2015.02.011

[156] Y. Mei, B. M. Beernink, E. E. Ellison, E. Konečná, A. K. Neelakandan, D. F. Voytas, and S. A. Whitham, “Protein expression and gene editing in monocots using foxtail mosaic virus vectors,” *Plant Direct*, vol. 3, article e00181, 2019 10.1002/pld3.181 PMC6874699 31768497

[157] X. Ma, X. Zhang, H. Liu, and Z. Li, “Highly efficient DNA-free plant genome editing using virally delivered CRISPR–Cas9,” *Nature Plants*, vol. 6, no. 7, pp. 773–779, 2020 32601419 10.1038/s41477-020-0704-5

[158] E. E. Ellison, U. Nagalakshmi, M. E. Gamo, P. J. Huang, S. Dinesh-Kumar, and D. F. Voytas, “Multiplexed heritable gene editing using RNA viruses and mobile single guide RNAs,” *Nature Plants*, vol. 6, no. 6, pp. 620–624, 2020 32483329 10.1038/s41477-020-0670-y

[159] G. S. Demirer, H. Zhang, N. S. Goh, R. L. Pinals, R. Chang, and M. P. Landry, “Carbon nanocarriers deliver siRNA to intact plant cells for efficient gene knockdown,” *Science Advances*, vol. 6, no. 26, article eaaz0495, 2020 10.1126/sciadv.aaz0495 PMC7314522 32637592

[160] D. Talukdar *Single-Cell Omics*, D. Barh, and V. Azevedo, Eds., Academic Press, San Diego, 2019

[161] J. Mergner, M. Frejno, M. List, M. Papacek, X. Chen, A. Chaudhary, P. Samaras, S. Richter, H. Shikata, M. Messerer, D. Lang, S. Altmann, P. Cyprys, D. P. Zolg, T. Mathieson, M. Bantscheff, R. R. Hazarika, T. Schmidt, C. Dawid, A. Dunkel, T. Hofmann, S. Sprunck, P. Falter-Braun, F. Johannes, K. F. X. Mayer, G. Jürgens, M. Wilhelm, J. Baumbach, E. Grill, K. Schneitz, C. Schwechheimer, and B. Kuster, “Mass-spectrometry-based draft of the *Arabidopsis* proteome,” *Nature*, vol. 579, no. 7799, pp. 409–414, 2020 32188942 10.1038/s41586-020-2094-2

[162] A. Kalia, and S. P. Sharma *Single-Cell Omics*, D. Barh, and V. Azevedo, Eds., Academic Press, San Diego, 2019

[163] V. Svensson, R. Vento-Tormo, and S. A. Teichmann, “Exponential scaling of single-cell RNA-seq in the past decade,” *Nature Protocols*, vol. 13, no. 4, pp. 599–604, 2018 29494575 10.1038/nprot.2017.149

[164] T. Denyer, X. Ma, S. Klesen, E. Scacchi, K. Nieselt, and M. C. P. Timmermans, “Spatiotemporal developmental trajectories in the *Arabidopsis* root revealed using high-throughput single-cell RNA sequencing,” *Developmental Cell*, vol. 48, no. 6, pp. 840–852.e5, 2019 30913408 10.1016/j.devcel.2019.02.022

[165] B. Budnik, E. Levy, G. Harmange, and N. Slavov, “SCoPE-MS: mass spectrometry of single mammalian cells quantifies proteome heterogeneity during cell differentiation,” *Genome Biology*, vol. 19, no. 1, p. 161, 2018 30343672 10.1186/s13059-018-1547-5 PMC6196420

[166] I. Efroni, and K. D. Birnbaum, “The potential of single-cell profiling in plants,” *Genome Biology*, vol. 17, no. 1, article 65, 2016 10.1186/s13059-016-0931-2 PMC4820866 27048384

[167] R. Zenobi, “Single-cell metabolomics: analytical and biological perspectives,” *Science*, vol. 342, no. 6163, article 1243259, 2013 10.1126/science.1243259 24311695

[168] M. Labib, and S. O. Kelley, “Single-cell analysis targeting the proteome,” *Nature Reviews Chemistry*, vol. 4, no. 3, pp. 143–158, 2020 37128021 10.1038/s41570-020-0162-7

[169] K. H. Ryu, L. Huang, H. M. Kang, and J. Schiefelbein, “Single-cell RNA sequencing resolves molecular relationships among individual plant cells,” *Plant Physiology*, vol. 179, no. 4, pp. 1444–1456, 2019 30718350 10.1104/pp.18.01482 PMC6446759

[170] C. Rich-Griffin, A. Stechemesser, J. Finch, E. Lucas, S. Ott, and P. Schäfer, “Single-cell transcriptomics: a high-resolution avenue for plant functional genomics,” *Trends in Plant Science*, vol. 25, no. 2, pp. 186–197, 2020 31780334 10.1016/j.tplants.2019.10.008

[171] C. N. Shulse, B. J. Cole, D. Ciobanu, J. Lin, Y. Yoshinaga, M. Gouran, G. M. Turco, Y. Zhu, R. C. O’Malley, S. M. Brady, and D. E. Dickel, “High-throughput single-cell transcriptome profiling of plant cell types,” *Cell Reports*, vol. 27, no. 7, pp. 2241–2247.e4, 2019 31091459 10.1016/j.celrep.2019.04.054 PMC6758921

[172] Y. Zhu, H. Li, S. Bhatti, S. Zhou, Y. Yang, T. Fish, and T. W. Thannhauser, “Development of a laser capture microscope-based single-cell-type proteomics tool for studying proteomes of individual cell layers of plant roots,” *Horticulture Research*, vol. 3, no. 1, article 16026, 2016 10.1038/hortres.2016.26 PMC4888759 27280026

[173] T. Fujii, S. Matsuda, M. L. Tejedor, T. Esaki, I. Sakane, H. Mizuno, N. Tsuyama, and T. Masujima, “Direct metabolomics for plant cells by live single-cell mass spectrometry,” *Nature Protocols*, vol. 10, no. 9, pp. 1445–1456, 2015 26313480 10.1038/nprot.2015.084

[174] H.-K. Choi, “Translational genomics and multi-omics integrated approaches as a useful strategy for crop breeding,” *Genes & Genomics*, vol. 41, no. 2, pp. 133–146, 2019 30353370 10.1007/s13258-018-0751-8 PMC6394800

[175] J. P. Wang, M. L. Matthews, C. M. Williams, R. Shi, C. Yang, S. Tunlaya-Anukit, H. C. Chen, Q. Li, J. Liu, C. Y. Lin, P. Naik, Y. H. Sun, P. L. Loziuk, T. F. Yeh, H. Kim, E. Gjersing, T. Shollenberger, C. M. Shuford, J. Song, Z. Miller, Y. Y. Huang, C. W. Edmunds, B. Liu, Y. Sun, Y. C. J. Lin, W. Li, H. Chen, I. Peszlen, J. J. Ducoste, J. Ralph, H. M. Chang, D. C. Muddiman, M. F. Davis, C. Smith, F. Isik, R. Sederoff, and V. L. Chiang, “Improving wood properties for wood utilization through multi-omics integration in lignin biosynthesis,” *Nature Communications*, vol. 9, no. 1, p. 1579, 2018 10.1038/s41467-018-03863-z PMC5910405 29679008

[176] B. B. Misra, C. Langefeld, M. Olivier, and L. A. Cox, “Integrated omics: tools, advances and future approaches,” *Journal of Molecular Endocrinology*, vol. 62, pp. R21–R45, 2019 10.1530/JME-18-0055 30006342

[177] T. Ma, and A. Zhang, “Integrate multi-omics data with biological interaction networks using Multi-view Factorization AutoEncoder (MAE),” *BMC Genomics*, vol. 20, no. S11, article 944, 2019 10.1186/s12864-019-6285-x PMC6923820 31856727

[178] A. Holzinger, B. Haibe-Kains, and I. Jurisica, “Why imaging data alone is not enough: AI-based integration of imaging, omics, and clinical data,” *European Journal of Nuclear Medicine and Molecular Imaging*, vol. 46, no. 13, pp. 2722–2730, 2019 31203421 10.1007/s00259-019-04382-9

[179] P. J. Kersey, “Plant genome sequences: past, present, future,” *Current Opinion in Plant Biology*, vol. 48, pp. 1–8, 2019 30579050 10.1016/j.pbi.2018.11.001

[180] T. D. Niehaus, A. M. K. Thamm, V. de Crécy-, and A. D. H. Lagard, “Proteins of unknown biochemical function: a persistent problem and a roadmap to help overcome it,” *Plant Physiology*, vol. 169, pp. 1436–1442, 2015 26269542 10.1104/pp.15.00959 PMC4634069

[181] S. Y. Rhee, and M. Mutwil, “Towards revealing the functions of all genes in plants,” *Trends in Plant Science*, vol. 19, no. 4, pp. 212–221, 2014 24231067 10.1016/j.tplants.2013.10.006

[182] D. Liu, M. Chen, B. Mendoza, H. Cheng, R. Hu, L. Li, C. T. Trinh, G. A. Tuskan, and X. Yang, “CRISPR/Cas9-mediated targeted mutagenesis for functional genomics research of crassulacean acid metabolism plants,” *Journal of Experimental Botany*, vol. 70, no. 22, pp. 6621–6629, 2019 31562521 10.1093/jxb/erz415 PMC6883263

[183] B. Minkenberg, K. Xie, and Y. Yang, “Discovery of rice essential genes by characterizing a CRISPR-edited mutation of closely related rice MAP kinase genes,” *Plant Journal*, vol. 89, no. 3, pp. 636–648, 2017 10.1111/tpj.13399 27747971

[184] A. R. Leydon, H. P. Gala, S. Guiziou, and J. L. Nemhauser, “Engineering synthetic signaling in plants,” *Annual Review of Plant Biology*, vol. 71, no. 1, pp. 767–788, 2020 10.1146/annurev-arplant-081519-035852 32092279

[185] X. Xiong, Y. Zhang, J. Yan, S. Jain, S. Chee, B. Ren, and H. Zhao, “A scalable epitope tagging approach for high throughput ChIP-Seq analysis,” *ACS Synthetic Biology*, vol. 6, no. 6, pp. 1034–1042, 2017 28215080 10.1021/acssynbio.6b00358 PMC5536957

[186] V. Armario Najera, R. M. Twyman, P. Christou, and C. Zhu, “Applications of multiplex genome editing in higher plants,” *Current Opinion in Biotechnology*, vol. 59, pp. 93–102, 2019 30978482 10.1016/j.copbio.2019.02.015

[187] Z. Luo, Q. Yang, B. Geng, S. Jiang, S. Yang, X. Li, Y. Cai, and J. Dai, “Whole genome engineering by synthesis,” *Science China Life Sciences*, vol. 61, no. 12, pp. 1515–1527, 2018 30465231 10.1007/s11427-018-9403-y

[188] J. Rees-Garbutt, O. Chalkley, S. Landon, O. Purcell, L. Marucci, and C. Grierson, “Designing minimal genomes using whole-cell models,” *Nature Communications*, vol. 11, no. 1, article 836, 2020 10.1038/s41467-020-14545-0 PMC7012841 32047145

[189] J. Zhou, R. Wu, X. Xue, and Z. Qin, “CasHRA (Cas9-facilitated Homologous Recombination Assembly) method of constructing megabase-sized DNA,” *Nucleic Acids Research*, vol. 44, no. 14, pp. e124–e124, 2016 27220470 10.1093/nar/gkw475 PMC5001600

[190] C. A. Hutchison, R. Y. Chuang, V. N. Noskov, N. Assad-Garcia, T. J. Deerinck, M. H. Ellisman, J. Gill, K. Kannan, B. J. Karas, L. Ma, J. F. Pelletier, Z. Q. Qi, R. A. Richter, E. A. Strychalski, L. Sun, Y. Suzuki, B. Tsvetanova, K. S. Wise, H. O. Smith, J. I. Glass, C. Merryman, D. G. Gibson, and J. C. Venter, “Design and synthesis of a minimal bacterial genome,” *Science*, vol. 351, no. 6280, article aad6253, 2016 10.1126/science.aad6253 27013737

[191] P. Lubrano, A. Danchin, and C. G. Acevedo-Rocha *Minimal Cells: Design, Construction, Biotechnological Applications*, A. R. Lara, and G. Gosset, Eds., Springer, Cham, 2020

[192] S. P. Long, S. Burgess, and I. Causton *Sustaining Global Food Security: The Nexus of Science and Policy*, R. S. Zeigler, Ed., CSIRO Publishing, Clayton South, 2019

[193] R. A. Voloshin, V. D. Kreslavski, S. K. Zharmukhamedov, V. S. Bedbenov, S. Ramakrishna, and S. I. Allakhverdiev, “Photoelectrochemical cells based on photosynthetic systems: a review,” *Biofuel Research Journal*, vol. 2, no. 2, pp. 227–235, 2015

[194] B. M. Wolf, and R. E. Blankenship, “Far-red light acclimation in diverse oxygenic photosynthetic organisms,” *Photosynthesis Research*, vol. 142, no. 3, pp. 349–359, 2019 31222688 10.1007/s11120-019-00653-6

[195] D. R. Ort, S. S. Merchant, J. Alric, A. Barkan, R. E. Blankenship, R. Bock, R. Croce, M. R. Hanson, J. M. Hibberd, S. P. Long, T. A. Moore, J. Moroney, K. K. Niyogi, M. A. J. Parry, P. P. Peralta-Yahya, R. C. Prince, K. E. Redding, M. H. Spalding, K. J. van Wijk, W. F. J. Vermaas, S. von Caemmerer, A. P. M. Weber, T. O. Yeates, J. S. Yuan, and X. G. Zhu, “Redesigning photosynthesis to sustainably meet global food and bioenergy demand,” *Proceedings of the National Academy of Sciences*, vol. 112, no. 28, pp. 8529–8536, 2015 10.1073/pnas.1424031112 PMC4507207 26124102

[196] A. J. Simkin, L. McAusland, T. Lawson, and C. A. Raines, “Overexpression of the RieskeFeS protein increases electron transport rates and biomass yield,” *Plant Physiology*, vol. 175, no. 1, pp. 134–145, 2017 28754840 10.1104/pp.17.00622 PMC5580758

[197] M. Ermakova, P. E. Lopez-Calcagno, C. A. Raines, R. T. Furbank, and S. von Caemmerer, “Overexpression of the Rieske FeS protein of the Cytochrome b6f complex increases C_4_ photosynthesis in *Setaria viridis*,” *Communications Biology*, vol. 2, no. 1, article 314, 2019 10.1038/s42003-019-0561-9 PMC6697696 31453378

[198] J.-H. Chen, S. T. Chen, N. Y. He, Q. L. Wang, Y. Zhao, W. Gao, and F. Q. Guo, “Nuclear-encoded synthesis of the D1 subunit of photosystem II increases photosynthetic efficiency and crop yield,” *Nature Plants*, vol. 6, no. 5, pp. 570–580, 2020 32313138 10.1038/s41477-020-0629-z

[199] J. Kromdijk, K. Głowacka, L. Leonelli, S. T. Gabilly, M. Iwai, K. K. Niyogi, and S. P. Long, “Improving photosynthesis and crop productivity by accelerating recovery from photoprotection,” *Science*, vol. 354, no. 6314, pp. 857–861, 2016 27856901 10.1126/science.aai8878

[200] A. Garcia-Molina, and D. Leister, “Accelerated relaxation of photoprotection impairs biomass accumulation in *Arabidopsis*,” *Nature Plants*, vol. 6, no. 1, pp. 9–12, 2020 31907400 10.1038/s41477-019-0572-z

[201] M. Betti, H. Bauwe, F. A. Busch, A. R. Fernie, O. Keech, M. Levey, D. R. Ort, M. A. J. Parry, R. Sage, S. Timm, B. Walker, and A. P. M. Weber, “Manipulating photorespiration to increase plant productivity: recent advances and perspectives for crop improvement,” *Journal of Experimental Botany*, vol. 67, no. 10, pp. 2977–2988, 2016 26951371 10.1093/jxb/erw076

[202] M. A. J. Parry, P. J. Andralojc, J. C. Scales, M. E. Salvucci, A. E. Carmo-Silva, H. Alonso, and S. M. Whitney, “Rubisco activity and regulation as targets for crop improvement,” *Journal of Experimental Botany*, vol. 64, no. 3, pp. 717–730, 2013 23162118 10.1093/jxb/ers336

[203] R. Kebeish, M. Niessen, K. Thiruveedhi, R. Bari, H. J. Hirsch, R. Rosenkranz, N. Stäbler, B. Schönfeld, F. Kreuzaler, and C. Peterhänsel, “Chloroplastic photorespiratory bypass increases photosynthesis and biomass production in *Arabidopsis thaliana*,” *Nature Biotechnology*, vol. 25, no. 5, pp. 593–599, 2007 10.1038/nbt1299 17435746

[204] A. Maier, H. Fahnenstich, S. von Caemmerer, M. K. M. Engqvist, A. P. M. Weber, U. I. Flügge, and V. G. Maurino, “Transgenic introduction of a glycolate oxidative cycle into *A. thaliana* chloroplasts leads to growth improvement,” *Frontiers in Plant Science*, vol. 3, 2012 10.3389/fpls.2012.00038 PMC3355595 22639647

[205] P. F. South, A. P. Cavanagh, H. W. Liu, and D. R. Ort, “Synthetic glycolate metabolism pathways stimulate crop growth and productivity in the field,” *Science*, vol. 363, no. 6422, article eaat9077, 2019 10.1126/science.aat9077 PMC7745124 30606819

[206] L.-M. Wang, B. R. Shen, B. D. Li, C. L. Zhang, M. Lin, P. P. Tong, L. L. Cui, Z. S. Zhang, and X. X. Peng, “A synthetic photorespiratory shortcut enhances photosynthesis to boost biomass and grain yield in rice,” *Molecular Plant*, 2020 10.1016/j.molp.2020.10.007 33075506

[207] V. G. Maurino, “Using energy-efficient synthetic biochemical pathways to bypass photorespiration,” *Biochemical Society Transactions*, vol. 47, no. 6, pp. 1805–1813, 2019 31754693 10.1042/BST20190322

[208] A. Bar-Even, E. Noor, N. E. Lewis, and R. Milo, “Design and analysis of synthetic carbon fixation pathways,” *Proceedings of the National Academy of Sciences*, vol. 107, no. 19, pp. 8889–8894, 2010 10.1073/pnas.0907176107 PMC2889323 20410460

[209] T. Schwander, L. Schada von Borzyskowski, S. Burgener, N. S. Cortina, and T. J. Erb, “A synthetic pathway for the fixation of carbon dioxide in vitro,” *Science*, vol. 354, no. 6314, pp. 900–904, 2016 27856910 10.1126/science.aah5237 PMC5892708

[210] T. E. Miller, T. Beneyton, T. Schwander, C. Diehl, M. Girault, R. McLean, T. Chotel, P. Claus, N. S. Cortina, J. C. Baret, and T. J. Erb, “Light-powered CO_2_ fixation in a chloroplast mimic with natural and synthetic parts,” *Science*, vol. 368, no. 6491, pp. 649–654, 2020 32381722 10.1126/science.aaz6802 PMC7610767

[211] T. J. Erb, P. R. Jones, and A. Bar-Even, “Synthetic metabolism: metabolic engineering meets enzyme design,” *Current Opinion in Chemical Biology*, vol. 37, pp. 56–62, 2017 28152442 10.1016/j.cbpa.2016.12.023 PMC7610756

[212] E. T. Wurtzel, C. E. Vickers, A. D. Hanson, A. H. Millar, M. Cooper, K. P. Voss-Fels, P. I. Nikel, and T. J. Erb, “Revolutionizing agriculture with synthetic biology,” *Nature Plants*, vol. 5, no. 12, pp. 1207–1210, 2019 31740769 10.1038/s41477-019-0539-0

[213] J. A. Raven, J. Beardall, and P. Sánchez-Baracaldo, “The possible evolution and future of CO_2_-concentrating mechanisms,” *Journal of Experimental Botany*, vol. 68, no. 14, pp. 3701–3716, 2017 28505361 10.1093/jxb/erx110

[214] S. C. Davis, J. Simpson, K. . C. Gil-Vega, N. A. Niechayev, E. . Tongerlo, N. H. Castano, L. V. Dever, and A. Búrquez, “Undervalued potential of crassulacean acid metabolism for current and future agricultural production,” *Journal of Experimental Botany*, vol. 70, no. 22, pp. 6521–6537, 2019 31087091 10.1093/jxb/erz223 PMC6883259

[215] M. Ermakova, F. R. Danila, R. T. Furbank, and S. von Caemmerer, “On the road to C_4_ rice: advances and perspectives,” *Plant Journal*, vol. 101, no. 4, pp. 940–950, 2020 10.1111/tpj.14562 PMC7065233 31596523

[216] I. Jurić, J. M. Hibberd, M. Blatt, and N. J. Burroughs, “Computational modelling predicts substantial carbon assimilation gains for C_3_ plants with a single-celled C_4_ biochemical pump,” *PLoS Computational Biology*, vol. 15, no. 9, article e1007373, 2019 10.1371/journal.pcbi.1007373 PMC6786660 31568503

[217] B. D. Rae, B. M. Long, B. Förster, N. D. Nguyen, C. N. Velanis, N. Atkinson, W. Y. Hee, B. Mukherjee, G. D. Price, and A. J. McCormick, “Progress and challenges of engineering a biophysical CO_2_-concentrating mechanism into higher plants,” *Journal of Experimental Botany*, vol. 68, no. 14, pp. 3717–3737, 2017 28444330 10.1093/jxb/erx133

[218] J. H. Hennacy, and M. C. Jonikas, “Prospects for engineering biophysical CO_2_ concentrating mechanisms into land plants to enhance yields,” *Annual Review of Plant Biology*, vol. 71, no. 1, pp. 461–485, 2020 10.1146/annurev-arplant-081519-040100 PMC7845915 32151155

[219] N. Atkinson, D. Feike, L. C. M. Mackinder, M. T. Meyer, H. Griffiths, M. C. Jonikas, A. M. Smith, and A. J. McCormick, “Introducing an algal carbon-concentrating mechanism into higher plants: location and incorporation of key components,” *Plant Biotechnology Journal*, vol. 14, no. 5, pp. 1302–1315, 2016 26538195 10.1111/pbi.12497 PMC5102585

[220] B. M. Long, W. Y. Hee, R. E. Sharwood, B. D. Rae, S. Kaines, Y. L. Lim, N. D. Nguyen, B. Massey, S. Bala, S. von Caemmerer, M. R. Badger, and G. D. Price, “Carboxysome encapsulation of the CO_2_-fixing enzyme Rubisco in tobacco chloroplasts,” *Nature Communications*, vol. 9, no. 1, p. 3570, 2018 10.1038/s41467-018-06044-0 PMC6120970 30177711

[221] N. Atkinson, C. N. Velanis, T. Wunder, D. J. Clarke, O. Mueller-Cajar, and A. J. McCormick, “The pyrenoidal linker protein EPYC1 phase separates with hybrid *Arabidopsis*–*Chlamydomonas* Rubisco through interactions with the algal Rubisco small subunit,” *Journal of Experimental Botany*, vol. 70, no. 19, pp. 5271–5285, 2019 31504763 10.1093/jxb/erz275 PMC6793452

[222] L. Wang, G. Ma, H. Wang, C. Cheng, S. Mu, W. Quan, L. Jiang, Z. Zhao, Y. Zhang, K. Zhang, X. Wang, C. Tian, and Y. Zhang, “A draft genome assembly of halophyte *Suaeda aralocaspica*, a plant that performs C_4_ photosynthesis within individual cells,” *GigaScience*, vol. 8, no. 9, 2019 10.1093/gigascience/giz116 PMC6741815 31513708

[223] M. R. Lundgren, “C_2_ photosynthesis: a promising route towards crop improvement?,” *New Phytologist*, 2020 10.1111/nph.16494 32080851

[224] C. Bellasio, and G. D. Farquhar, “A leaf-level biochemical model simulating the introduction of C_2_ and C_4_ photosynthesis in C_3_ rice: gains, losses and metabolite fluxes,” *New Phytologist*, vol. 223, no. 1, pp. 150–166, 2019 30859576 10.1111/nph.15787

[225] J. S. Amthor, A. Bar-Even, A. D. Hanson, A. H. Millar, M. Stitt, L. J. Sweetlove, and S. D. Tyerman, “Engineering strategies to boost crop productivity by cutting respiratory carbon loss,” *Plant Cell*, vol. 31, no. 2, pp. 297–314, 2019 30670486 10.1105/tpc.18.00743 PMC6447004

[226] Q. Zhu, B. Wang, J. Tan, T. Liu, L. Li, and Y. G. Liu, “Plant synthetic metabolic engineering for enhancing crop nutritional quality,” *Plant Communications*, vol. 1, no. 1, article 100017, 2020 10.1016/j.xplc.2019.100017 PMC7747972 33404538

[227] S. Fahad, A. A. Bajwa, U. Nazir, S. A. Anjum, A. Farooq, A. Zohaib, S. Sadia, W. Nasim, S. Adkins, S. Saud, M. Z. Ihsan, H. Alharby, C. Wu, D. Wang, and J. Huang, “Crop production under drought and heat stress: plant responses and management options,” *Frontiers in Plant Science*, vol. 8, 2017 10.3389/fpls.2017.01147 PMC5489704 28706531

[228] M. Ashraf, M. S. A. Ahmad, M. Öztürk, and A. Aksoy *Crop Production for Agricultural Improvement*, M. Ashraf, M. Öztürk, M. S. A. Ahmad, and A. Aksoy, Eds., Springer, Netherlands, Dordrecht, 2012

[229] J. Mizoi, and K. Yamaguchi-Shinozaki *Rice Protocols*, Y. Yang, Ed., Humana Press, Totowa, NJ, vol. 956, 2013

[230] F. Marco, M. Bitrián, P. Carrasco, M. V. Rajam, R. Alcázar, and A. F. Tiburcio *Plant Biology and Biotechnology: Volume II: Plant Genomics and Biotechnology*, B. Bahadur, M. V. Rajam, L. Sahijram, and K. V. Krishnamurthy, Eds., Springer India, New Delhi, 2015

[231] A. M. Borland, H. Griffiths, J. Hartwell, and J. A. C. Smith, “Exploiting the potential of plants with crassulacean acid metabolism for bioenergy production on marginal lands,” *Journal of Experimental Botany*, vol. 60, no. 10, pp. 2879–2896, 2009 19395392 10.1093/jxb/erp118

[232] S. C. Davis, D. S. LeBauer, and S. P. Long, “Light to liquid fuel: theoretical and realized energy conversion efficiency of plants using Crassulacean Acid Metabolism (CAM) in arid conditions,” *Journal of Experimental Botany*, vol. 65, no. 13, pp. 3471–3478, 2014 24744431 10.1093/jxb/eru163

[233] A. M. Borland, J. Hartwell, D. J. Weston, K. A. Schlauch, T. J. Tschaplinski, G. A. Tuskan, X. Yang, and J. C. Cushman, “Engineering crassulacean acid metabolism to improve water-use efficiency,” *Trends in Plant Science*, vol. 19, no. 5, pp. 327–338, 2014 24559590 10.1016/j.tplants.2014.01.006 PMC4065858

[234] D. Liu, K. J. Palla, R. Hu, R. C. Moseley, C. Mendoza, M. Chen, P. E. Abraham, J. L. Labbé, U. C. Kalluri, T. J. Tschaplinski, J. C. Cushman, A. M. Borland, G. A. Tuskan, and X. Yang, “Perspectives on the basic and applied aspects of crassulacean acid metabolism (CAM) research,” *Plant Science*, vol. 274, pp. 394–401, 2018 30080627 10.1016/j.plantsci.2018.06.012

[235] X. Yang, J. C. Cushman, A. M. Borland, E. J. Edwards, S. D. Wullschleger, G. A. Tuskan, N. A. Owen, H. Griffiths, J. A. C. Smith, H. C. de Paoli, D. J. Weston, R. Cottingham, J. Hartwell, S. C. Davis, K. Silvera, R. Ming, K. Schlauch, P. Abraham, J. R. Stewart, H. B. Guo, R. Albion, J. Ha, S. D. Lim, B. W. M. Wone, W. C. Yim, T. Garcia, J. A. Mayer, J. Petereit, S. S. Nair, E. Casey, R. L. Hettich, J. Ceusters, P. Ranjan, K. J. Palla, H. Yin, C. Reyes-García, J. L. Andrade, L. Freschi, J. D. Beltrán, L. V. Dever, S. F. Boxall, J. Waller, J. Davies, P. Bupphada, N. Kadu, K. Winter, R. F. Sage, C. N. Aguilar, J. Schmutz, J. Jenkins, and J. A. M. Holtum, “A roadmap for research on crassulacean acid metabolism (CAM) to enhance sustainable food and bioenergy production in a hotter, drier world,” *New Phytologist*, vol. 207, no. 3, pp. 491–504, 2015 26153373 10.1111/nph.13393

[236] X. Yang, J. C. Cushman, A. M. Borland, and Q. Liu, “Editorial: systems biology and synthetic biology in relation to drought tolerance or avoidance in plants,” *Frontiers in Plant Science*, vol. 11, no. 394, 2020 10.3389/fpls.2020.00394 PMC7161431 32328077

[237] A. Giannakopoulou, A. Bialas, S. Kamoun, and V. G. A. A. Vleeshouwers, “Plant immunity switched from bacteria to virus,” *Nature Biotechnology*, vol. 34, no. 4, pp. 391–392, 2016 10.1038/nbt.3538 27054993

[238] S. Horsefield, H. Burdett, X. Zhang, M. K. Manik, Y. Shi, J. Chen, T. Qi, J. Gilley, J. S. Lai, M. X. Rank, L. W. Casey, W. Gu, D. J. Ericsson, G. Foley, R. O. Hughes, T. Bosanac, M. von Itzstein, J. P. Rathjen, J. D. Nanson, M. Boden, I. B. Dry, S. J. Williams, B. J. Staskawicz, M. P. Coleman, T. Ve, P. N. Dodds, and B. Kobe, “NAD^+^ cleavage activity by animal and plant TIR domains in cell death pathways,” *Science*, vol. 365, no. 6455, pp. 793–799, 2019 31439792 10.1126/science.aax1911

[239] L. Wan, K. Essuman, R. G. Anderson, Y. Sasaki, F. Monteiro, E. H. Chung, E. Osborne Nishimura, A. DiAntonio, J. Milbrandt, J. L. Dangl, and M. T. Nishimura, “TIR domains of plant immune receptors are NAD^+^-cleaving enzymes that promote cell death,” *Science*, vol. 365, no. 6455, pp. 799–803, 2019 31439793 10.1126/science.aax1771 PMC7045805

[240] A. M. R. Gatehouse, N. Ferry, M. G. Edwards, and H. A. Bell, “Insect-resistant biotech crops and their impacts on beneficial arthropods,” *Philosophical Transactions of the Royal Society B: Biological Sciences*, vol. 366, no. 1569, pp. 1438–1452, 2011 10.1098/rstb.2010.0330 PMC3081576 21444317

[241] B. E. Tabashnik, T. Brévault, and Y. Carrière, “Insect resistance to Bt crops: lessons from the first billion acres,” *Nature Biotechnology*, vol. 31, no. 6, pp. 510–521, 2013 10.1038/nbt.2597 23752438

[242] X.-D. Yu, Z. C. Liu, S. L. Huang, Z. Q. Chen, Y. W. Sun, P. F. Duan, Y. Z. Ma, and L. Q. Xia, “RNAi-mediated plant protection against aphids,” *Pest Management Science*, vol. 72, no. 6, pp. 1090–1098, 2016 26888776 10.1002/ps.4258

[243] S. S. Porter, R. Bantay, C. A. Friel, A. Garoutte, K. Gdanetz, K. Ibarreta, B. M. Moore, P. Shetty, E. Siler, and M. L. Friesen, “Beneficial microbes ameliorate abiotic and biotic sources of stress on plants,” *Functional Ecology*, vol. 34, no. 10, pp. 2075–2086, 2020

[244] F. Mus, M. B. Crook, K. Garcia, A. Garcia Costas, B. A. Geddes, E. D. Kouri, P. Paramasivan, M. H. Ryu, G. E. D. Oldroyd, P. S. Poole, M. K. Udvardi, C. A. Voigt, J. M. Ané, and J. W. Peters, “Symbiotic nitrogen fixation and the challenges to its extension to nonlegumes,” *Applied and Environmental Microbiology*, vol. 82, no. 13, pp. 3698–3710, 2016 27084023 10.1128/AEM.01055-16 PMC4907175

[245] B. A. Geddes, P. Paramasivan, A. Joffrin, A. L. Thompson, K. Christensen, B. Jorrin, P. Brett, S. J. Conway, G. E. D. Oldroyd, and P. S. Poole, “Engineering transkingdom signalling in plants to control gene expression in rhizosphere bacteria,” *Nature Communications*, vol. 10, no. 1, article 3430, 2019 10.1038/s41467-019-10882-x PMC6668481 31366919

[246] E. M. Wiseman, S. Bar-El Dadon, and R. Reifen, “The vicious cycle of vitamin a deficiency: a review,” *Critical Reviews in Food Science and Nutrition*, vol. 57, no. 17, pp. 3703–3714, 2017 27128154 10.1080/10408398.2016.1160362

[247] J. Umbreit, “Iron deficiency: a concise review,” *American Journal of Hematology*, vol. 78, no. 3, pp. 225–231, 2005 15726599 10.1002/ajh.20249

[248] A. E. Czeizel, I. Dudás, A. Vereczkey, and F. Bánhidy, “Folate deficiency and folic acid supplementation: the prevention of neural-tube defects and congenital heart defects,” *Nutrients*, vol. 5, no. 11, pp. 4760–4775, 2013 24284617 10.3390/nu5114760 PMC3847759

[249] United Nations Department of Economic and Social Affairs *Millennium Development Goals Report 2009*, United Nations Publications, New York, 2009

[250] M.-S. Roell, and M. D. Zurbriggen, “The impact of synthetic biology for future agriculture and nutrition,” *Current Opinion in Biotechnology*, vol. 61, pp. 102–109, 2020 31812911 10.1016/j.copbio.2019.10.004

[251] J.-Y. Paul, H. Khanna, J. Kleidon, P. Hoang, J. Geijskes, J. Daniells, E. Zaplin, Y. Rosenberg, A. James, B. Mlalazi, P. Deo, G. Arinaitwe, P. Namanya, D. Becker, J. Tindamanyire, W. Tushemereirwe, R. Harding, and J. Dale, “Golden bananas in the field: elevated fruit pro-vitamin A from the expression of a single banana transgene,” *Plant Biotechnology Journal*, vol. 15, no. 4, pp. 520–532, 2017 27734628 10.1111/pbi.12650 PMC5362681

[252] E. T. Wurtzel, “Changing form and function through carotenoids and synthetic biology,” *Plant Physiology*, vol. 179, no. 3, pp. 830–843, 2019 30361256 10.1104/pp.18.01122 PMC6393808

[253] H. Masuda, Y. Ishimaru, M. S. Aung, T. Kobayashi, Y. Kakei, M. Takahashi, K. Higuchi, H. Nakanishi, and N. K. Nishizawa, “Iron biofortification in rice by the introduction of multiple genes involved in iron nutrition,” *Scientific Reports*, vol. 2, no. 1, article 543, 2012 10.1038/srep00543 PMC3408131 22848789

[254] R. I. Díaz de la Garza, J. F. Gregory, and A. D. Hanson, “Folate biofortification of tomato fruit,” *Proceedings of the National Academy of Sciences*, vol. 104, no. 10, pp. 4218–4222, 2007 10.1073/pnas.0700409104 PMC1810332 17360503

[255] L. Mène-Saffrané, and S. Pellaud, “Current strategies for vitamin E biofortification of crops,” *Current Opinion in Biotechnology*, vol. 44, pp. 189–197, 2017 28327426 10.1016/j.copbio.2017.01.007

[256] P. White, and M. Broadley, “Physiological limits to zinc biofortification of edible crops,” *Frontiers in Plant Science*, vol. 2, 2011 10.3389/fpls.2011.00080 PMC3355814 22645552

[257] K. Sinha, and V. Khare, “Review on: antinutritional factors in vegetable crops,” *Pharma Innovation Journal*, vol. 12, pp. 353–358, 2017

[258] R. Sharma, S. Bharti, and K. V. S. Kumar, “Diet and thyroid - myths and facts,” *Journal of Medical Nutrition and Nutraceuticals*, vol. 3, pp. 60–65, 2014

[259] A. Gonçalves, P. Goufo, A. Barros, R. Domínguez-Perles, H. Trindade, E. A. S. Rosa, L. Ferreira, and M. Rodrigues, “Cowpea (*Vigna unguiculata* L. Walp), a renewed multipurpose crop for a more sustainable agri-food system: nutritional advantages and constraints,” *Journal of the Science of Food and Agriculture*, vol. 96, no. 9, pp. 2941–2951, 2016 26804459 10.1002/jsfa.7644

[260] IPCC *Core Writing Team*, R. Pachauri, and L. Meyer, Eds., IPCC, Geneva, Switzerland, 2014

[261] J. Rogelj, P. M. Forster, E. Kriegler, C. J. Smith, and R. Séférian, “Estimating and tracking the remaining carbon budget for stringent climate targets,” *Nature*, vol. 571, no. 7765, pp. 335–342, 2019 31316194 10.1038/s41586-019-1368-z

[262] C. DeLisi, “The role of synthetic biology in climate change mitigation,” *Biology Direct*, vol. 14, no. 1, p. 14, 2019 31429783 10.1186/s13062-019-0247-8 PMC6700980

[263] C. DeLisi, A. Patrinos, M. MacCracken, D. Drell, G. Annas, A. Arkin, G. Church, R. Cook-Deegan, H. Jacoby, M. Lidstrom, J. Melillo, R. Milo, K. Paustian, J. Reilly, R. J. Roberts, D. Segrè, S. Solomon, D. Woolf, S. D. Wullschleger, and X. Yang, “The role of synthetic biology in atmospheric greenhouse gas reduction: prospects and challenges,” *BioDesign Research*, vol. 2020, pp. 1–8, 2020 10.34133/2020/1016207 PMC10521736 37849905

[264] U. C. Kalluri, X. Yang, and S. D. Wullschleger, “Plant biosystems design for a carbon-neutral bioeconomy,” *BioDesign Research*, vol. 2020, pp. 1–5, 2020 10.34133/2020/7914051 PMC10521676 37849896

[265] B. Baker, “Can modern agriculture be sustainable?,” *BioScience*, vol. 67, no. 4, pp. 325–331, 2017

[266] T. Ma, G. Dai, S. Zhu, D. Chen, L. Chen, X. Lü, X. Wang, J. Zhu, Y. Zhang, W. Ma, J. S. He, Y. Bai, X. Han, and X. Feng, “Distribution and preservation of root- and shoot-derived carbon components in soils across the Chinese-Mongolian grasslands,” *Journal of Geophysical Research: Biogeosciences*, vol. 124, no. 2, pp. 420–431, 2019

[267] R. Blaustein, “Sowing seeds for carbon sequestration,” *Bioscience*, vol. 69, no. 5, pp. 404–404, 2019

[268] H. Harwatt, “Including animal to plant protein shifts in climate change mitigation policy: a proposed three-step strategy,” *Climate Policy*, vol. 19, no. 5, pp. 533–541, 2019

[269] J.-F. Bastin, Y. Finegold, C. Garcia, D. Mollicone, M. Rezende, D. Routh, C. M. Zohner, and T. W. Crowther, “The global tree restoration potential,” *Science*, vol. 365, no. 6448, pp. 76–79, 2019 31273120 10.1126/science.aax0848

[270] F. Abbas-Aghababazadeh, Q. Mo, and B. L. Fridley, “Statistical genomics in rare cancer,” *Seminars in Cancer Biology*, vol. 61, pp. 1–10, 2020 31437624 10.1016/j.semcancer.2019.08.021 PMC7771546

[271] H. He, E. J. Veneklaas, J. Kuo, and H. Lambers, “Physiological and ecological significance of biomineralization in plants,” *Trends in Plant Science*, vol. 19, no. 3, pp. 166–174, 2014 24291440 10.1016/j.tplants.2013.11.002

[272] C. Jansson, T. Mockler, J. Vogel, H. DePaoli, S. Hazen, V. Srinivasan, A. Cousins, P. Lemaux, J. Dahlberg, and T. Brutnell *Oxygen Production and Reduction in Artificial and Natural Systems*, J. Barber, A. V. Ruban, and P. J. Nixon, Eds., World Scientific Publishing, Singapore, 2019

[273] U.S. DOE *Genome Engineering for Materials Synthesis Workshop Report. DOE/SC-0198*, Department of Energy Office of Science, U.S, 2019

[274] A. Harfouche, R. Meilan, and A. Altman, “Tree genetic engineering and applications to sustainable forestry and biomass production,” *Trends in Biotechnology*, vol. 29, no. 1, pp. 9–17, 2011 20970211 10.1016/j.tibtech.2010.09.003

[275] S. K. Ramamoorthy, M. Skrifvars, and A. Persson, “A review of natural fibers used in biocomposites: plant, animal and regenerated cellulose fibers,” *Polymer Reviews*, vol. 55, no. 1, pp. 107–162, 2015

[276] P. Singh, Y.-J. Kim, D. Zhang, and D.-C. Yang, “Biological synthesis of nanoparticles from plants and microorganisms,” *Trends in Biotechnology*, vol. 34, no. 7, pp. 588–599, 2016 26944794 10.1016/j.tibtech.2016.02.006

[277] W. Fang, S. Yang, X.-L. Wang, T.-Q. Yuan, and R.-C. Sun, “Manufacture and application of lignin-based carbon fibers (LCFs) and lignin-based carbon nanofibers (LCNFs),” *Green Chemistry*, vol. 19, no. 8, pp. 1794–1827, 2017

[278] S. Campuzano, and A. E. Pelling, “Scaffolds for 3D cell culture and cellular agriculture applications derived from non-animal sources,” *Frontiers in Sustainable Food Systems*, vol. 3, p. 38, 2019

[279] T. H. Jovic, G. Kungwengwe, A. C. Mills, and I. S. Whitaker, “Plant-derived biomaterials: a review of 3D bioprinting and biomedical applications,” *Frontiers in Mechanical Engineering*, vol. 5, p. 19, 2019

[280] H. Karan, C. Funk, M. Grabert, M. Oey, and B. Hankamer, “Green bioplastics as part of a circular bioeconomy,” *Trends in Plant Science*, vol. 24, no. 3, pp. 237–249, 2019 30612789 10.1016/j.tplants.2018.11.010

[281] R. Mohammadinejad, H. Maleki, E. Larrañeta, A. R. Fajardo, A. B. Nik, A. Shavandi, A. Sheikhi, M. Ghorbanpour, M. Farokhi, P. Govindh, E. Cabane, S. Azizi, A. R. Aref, M. Mozafari, M. Mehrali, S. Thomas, J. F. Mano, Y. K. Mishra, and V. K. Thakur, “Status and future scope of plant-based green hydrogels in biomedical engineering,” *Applied Materials Today*, vol. 16, pp. 213–246, 2019

[282] S. Bertella, and J. S. Luterbacher, “Lignin functionalization for the production of novel materials,” *Trends in Chemistry*, vol. 2, no. 5, pp. 440–453, 2020

[283] P. A. Nakata, “Calcium oxalate crystal morphology,” *Trends in Plant Science*, vol. 7, no. 7, p. 324, 2002 12119171 10.1016/s1360-1385(02)02285-9

[284] J. Sun, and B. Bhushan, “Hierarchical structure and mechanical properties of nacre: a review,” *RSC Advances*, vol. 2, no. 20, pp. 7617–7632, 2012

[285] A. Abd, “Studying the mechanical and electrical properties of epoxy with PVC and calcium carbonate filler,” *International Journal of Engineering & Technology*, vol. 3, no. 4, pp. 545–553, 2014

[286] C. Lertvachirapaiboon, P. Pienpinijtham, K. Wongravee, and S. Ekgasit, “Optical properties of individual aragonite plates from nacre,” *ChemistrySelect*, vol. 3, no. 41, pp. 11700–11704, 2018

[287] W. Sun, S. Jayaraman, W. Chen, K. A. Persson, and G. Ceder, “Nucleation of metastable aragonite CaCO_3_ in seawater,” *Proceedings of the National Academy of Sciences*, vol. 112, no. 11, pp. 3199–3204, 2015 10.1073/pnas.1423898112 PMC4371997 25739963

[288] R. Davies, O. Teall, M. Pilegis, A. Kanellopoulos, T. Sharma, A. Jefferson, D. Gardner, A. al-Tabbaa, K. Paine, and R. Lark, “Large scale application of self-healing concrete: design, construction, and testing,” *Frontiers in Materials*, vol. 5, p. 51, 2018

[289] T. Chen, P. Shi, Y. Li, T. Duan, Y. Yu, X. Li, and W. Zhu, “Biomineralization of varied calcium carbonate crystals by the synergistic effect of silk fibroin/magnesium ions in a microbial system,” *CrystEngComm*, vol. 20, no. 17, pp. 2366–2373, 2018

[290] K. Daicho, T. Saito, S. Fujisawa, and A. Isogai, “The crystallinity of nanocellulose: dispersion-induced disordering of the grain boundary in biologically structured cellulose,” *ACS Applied Nano Materials*, vol. 1, no. 10, pp. 5774–5785, 2018

[291] V. G. Vandavasi, D. K. Putnam, Q. Zhang, L. Petridis, W. T. Heller, B. T. Nixon, C. H. Haigler, U. Kalluri, L. Coates, P. Langan, J. C. Smith, J. Meiler, and H. O’Neill, “A structural study of CESA1 catalytic domain of *Arabidopsis* cellulose synthesis complex: evidence for CESA trimers,” *Plant Physiology*, vol. 170, pp. 123–135, 2015 26556795 10.1104/pp.15.01356 PMC4704586

[292] C. P. Joshi, S. Thammannagowda, T. Fujino, J. Q. Gou, U. Avci, C. H. Haigler, L. M. McDonnell, S. D. Mansfield, B. Mengesha, N. C. Carpita, D. Harris, S. DeBolt, and G. F. Peter, “Perturbation of wood cellulose synthesis causes pleiotropic effects in transgenic aspen,” *Molecular Plant*, vol. 4, no. 2, pp. 331–345, 2011 21300756 10.1093/mp/ssq081

[293] X. Zhang, P. G. Dominguez, M. Kumar, J. Bygdell, S. Miroshnichenko, B. Sundberg, G. Wingsle, and T. Niittylä, “Cellulose synthase stoichiometry in aspen differs from *Arabidopsis* and Norway spruce,” *Plant Physiology*, vol. 177, no. 3, pp. 1096–1107, 2018 29760198 10.1104/pp.18.00394 PMC6053019

[294] H. Lu, G. Yuan, S. H. Strauss, T. J. Tschaplinski, G. A. Tuskan, J. G. Chen, and X. Yang, “Reconfiguring plant metabolism for biodegradable plastic production,” *BioDesign Research*, vol. 2020, pp. 1–13, 2020 10.34133/2020/9078303 PMC10530661 37849903

[295] N. Savage, “Fuel options: the ideal biofuel,” *Nature*, vol. 474, no. 7352, pp. S9–S11, 2011 10.1038/474S09a 21697843

[296] D. Rathore, A.-S. Nizami, A. Singh, and D. Pant, “Key issues in estimating energy and greenhouse gas savings of biofuels: challenges and perspectives,” *Biofuel Research Journal*, vol. 3, no. 2, pp. 380–393, 2016

[297] M. W. Rosegrant *Biofuels and Grain Prices: Impacts and Policy Responses*, International Food Policy Research Institute, Washington, DC, 2008

[298] L. German, A. Goetz, T. Searchinger, G. . L. T. Oliveira, J. Tomei, C. Hunsberger, and J. Weigelt, “*Sine Qua Nons* of sustainable biofuels: distilling implications of under-performance for national biofuel programs,” *Energy Policy*, vol. 108, pp. 806–817, 2017

[299] K. Markel, M. S. Belcher, and P. M. Shih, “Defining and engineering bioenergy plant feedstock ideotypes,” *Current Opinion in Biotechnology*, vol. 62, pp. 196–201, 2020 31841969 10.1016/j.copbio.2019.11.014

[300] M. A. Díaz-Pérez, and J. C. Serrano-Ruiz, “Catalytic production of jet fuels from biomass,” *Molecules*, vol. 25, no. 4, p. 802, 2020 32059552 10.3390/molecules25040802 PMC7071043

[301] P. McKendry, “Energy production from biomass (part 1): overview of biomass,” *Bioresource Technology*, vol. 83, no. 1, pp. 37–46, 2002 12058829 10.1016/s0960-8524(01)00118-3

[302] G. Perin, and P. R. Jones, “Economic feasibility and long-term sustainability criteria on the path to enable a transition from fossil fuels to biofuels,” *Current Opinion in Biotechnology*, vol. 57, pp. 175–182, 2019 31103911 10.1016/j.copbio.2019.04.004

[303] J. Suman, O. Uhlik, J. Viktorova, and T. Macek, “Phytoextraction of heavy metals: a promising tool for clean-up of polluted environment?,” *Frontiers in Plant Science*, vol. 9, p. 1476, 2018 30459775 10.3389/fpls.2018.01476 PMC6232834

[304] A. van der Ent, A. J. M. Baker, R. D. Reeves, A. J. Pollard, and H. Schat, “Hyperaccumulators of metal and metalloid trace elements: facts and fiction,” *Plant and Soil*, vol. 362, pp. 319–334, 2013

[305] R. D. Reeves, A. J. M. Baker, T. Jaffré, P. D. Erskine, G. Echevarria, and A. van der Ent, “A global database for plants that hyperaccumulate metal and metalloid trace elements,” *New Phytologist*, vol. 218, no. 2, pp. 407–411, 2018 29139134 10.1111/nph.14907

[306] N. Rascio, and F. Navari-Izzo, “Heavy metal hyperaccumulating plants: how and why do they do it? And what makes them so interesting?,” *Plant Science*, vol. 180, no. 2, pp. 169–181, 2011 21421358 10.1016/j.plantsci.2010.08.016

[307] H. Ali, E. Khan, and M. A. Sajad, “Phytoremediation of heavy metals—concepts and applications,” *Chemosphere*, vol. 91, no. 7, pp. 869–881, 2013 23466085 10.1016/j.chemosphere.2013.01.075

[308] A. van der Ent, A. J. Baker, R. D. Reeves, R. L. Chaney, C. W. Anderson, J. A. Meech, P. D. Erskine, M. O. Simonnot, J. Vaughan, J. L. Morel, and G. Echevarria, “Agromining: farming for metals in the future?,” *Environmental Science & Technology*, vol. 49, no. 8, pp. 4773–4780, 2015 25700109 10.1021/es506031u

[309] G. Narender Reddy, and M. N. V. Prasad, “Heavy metal-binding proteins/peptides: occurrence, structure, synthesis and functions. A review,” *Environmental and Experimental Botany*, vol. 30, no. 3, pp. 251–264, 1990

[310] G. Tyler, “Rare earth elements in soil and plant systems - a review,” *Plant and Soil*, vol. 267, no. 1-2, pp. 191–206, 2004

[311] C. Turra, E. A. De Nadai Fernandes, M. A. Bacchi, G. A. Sarriés, and A. E. L. Reyes, “Uptake of rare earth elements by citrus plants from phosphate fertilizers,” *Plant and Soil*, vol. 437, no. 1-2, pp. 291–299, 2019

[312] P. Mikołajczak, K. Borowiak, and P. Niedzielski, “Phytoextraction of rare earth elements in herbaceous plant species growing close to roads,” *Environmental Science and Pollution Research International*, vol. 24, no. 16, pp. 14091–14103, 2017 28411316 10.1007/s11356-017-8944-2 PMC5486614

[313] J. B. Calixto, “The role of natural products in modern drug discovery,” *Anais da Academia Brasileira de Ciências*, vol. 91, Suppl 3, p. e20190105, 2019 31166478 10.1590/0001-3765201920190105

[314] J. C. Mortimer, “Plant synthetic biology could drive a revolution in biofuels and medicine,” *Experimental Biology and Medicine*, vol. 244, no. 4, pp. 323–331, 2019 30249124 10.1177/1535370218793890 PMC6435885

[315] K. Brook, J. Bennett, and S. P. Desai, “The chemical history of morphine: an 8000-year journey, from resin to de-novo synthesis,” *Journal of Anesthesia History*, vol. 3, no. 2, pp. 50–55, 2017 28641826 10.1016/j.janh.2017.02.001

[316] I. A. Graham, K. Besser, S. Blumer, C. A. Branigan, T. Czechowski, L. Elias, I. Guterman, D. Harvey, P. G. Isaac, A. M. Khan, T. R. Larson, Y. Li, T. Pawson, T. Penfield, A. M. Rae, D. A. Rathbone, S. Reid, J. Ross, M. F. Smallwood, V. Segura, T. Townsend, D. Vyas, T. Winzer, and D. Bowles, “The genetic map of *Artemisia annua* L. identifies loci affecting yield of the antimalarial drug artemisinin,” *Science*, vol. 327, no. 5963, pp. 328–331, 2010 20075252 10.1126/science.1182612

[317] N. K. B. K. Ikram, and H. T. Simonsen, “A review of biotechnological artemisinin production in plants,” *Frontiers in Plant Science*, vol. 8, no. 1966, 2017 10.3389/fpls.2017.01966 PMC5694819 29187859

[318] R. Mohammadinejad, A. Shavandi, D. S. Raie, J. Sangeetha, M. Soleimani, S. Shokrian Hajibehzad, D. Thangadurai, R. Hospet, J. O. Popoola, A. Arzani, M. A. Gómez-Lim, S. Iravani, and R. S. Varma, “Plant molecular farming: production of metallic nanoparticles and therapeutic proteins using green factories,” *Green Chemistry*, vol. 21, no. 8, pp. 1845–1865, 2019

[319] G. P. Lomonossoff, and M. A. D'Aoust, “Plant-produced biopharmaceuticals: a case of technical developments driving clinical deployment,” *Science*, vol. 353, no. 6305, pp. 1237–1240, 2016 27634524 10.1126/science.aaf6638

[320] V. Virdi, and A. Depicker, “Role of plant expression systems in antibody production for passive immunization,” *International Journal of Developmental Biology*, vol. 57, no. 6-7-8, pp. 587–593, 2013 24166441 10.1387/ijdb.130266ad

[321] S. R. Karg, and P. T. Kallio, “The production of biopharmaceuticals in plant systems,” *Biotechnology Advances*, vol. 27, no. 6, pp. 879–894, 2009 19647060 10.1016/j.biotechadv.2009.07.002

[322] R. Fischer, E. Stoger, S. Schillberg, P. Christou, and R. M. Twyman, “Plant-based production of biopharmaceuticals,” *Current Opinion in Plant Biology*, vol. 7, no. 2, pp. 152–158, 2004 15003215 10.1016/j.pbi.2004.01.007

[323] M. Chen, X. Liu, Z. Wang, J. Song, Q. Qi, and P. G. Wang, “Modification of plant N-glycans processing: the future of producing therapeutic protein by transgenic plants,” *Medicinal Research Reviews*, vol. 25, no. 3, pp. 343–360, 2005 15499575 10.1002/med.20022

[324] C. Arntzen, “Plant-made pharmaceuticals: from ‘Edible Vaccines’ to Ebola therapeutics,” *Plant Biotechnology Journal*, vol. 13, no. 8, pp. 1013–1016, 2015 26345276 10.1111/pbi.12460 PMC5049623

[325] J. Yao, Y. Weng, A. Dickey, and K. Y. Wang, “Plants as factories for human pharmaceuticals: applications and challenges,” *International Journal of Molecular Sciences*, vol. 16, no. 12, pp. 28549–28565, 2015 26633378 10.3390/ijms161226122 PMC4691069

[326] T. Capell, R. M. Twyman, V. Armario-Najera, J. K. C. Ma, S. Schillberg, and P. Christou, “Potential applications of plant biotechnology against SARS-CoV-2,” *Trends in Plant Science*, vol. 25, no. 7, pp. 635–643, 2020 32371057 10.1016/j.tplants.2020.04.009 PMC7181989

[327] N. King, C. T. Hittinger, and S. B. Carroll, “Evolution of key cell signaling and adhesion protein families predates animal origins,” *Science*, vol. 301, no. 5631, pp. 361–363, 2003 12869759 10.1126/science.1083853

[328] J. O. Woods, U. M. Singh-Blom, J. M. Laurent, K. L. McGary, and E. M. Marcotte, “Prediction of gene-phenotype associations in humans, mice, and plants using phenologs,” *BMC Bioinformatics*, vol. 14, no. 1, p. 203, 2013 23800157 10.1186/1471-2105-14-203 PMC3704650

[329] E. J. Klekowski, “Genetic load and its causes in long-lived plants,” *Trees*, vol. 2, no. 4, pp. 195–203, 1988

[330] G. A. Tuskan, R. Mewalal, L. E. Gunter, K. J. Palla, K. Carter, D. A. Jacobson, P. C. Jones, B. J. Garcia, D. A. Weighill, P. D. Hyatt, Y. Yang, J. Zhang, N. Reis, J. G. Chen, and W. Muchero, “Defining the genetic components of callus formation: a GWAS approach,” *PLoS One*, vol. 13, no. 8, article e0202519, 2018 10.1371/journal.pone.0202519 PMC6097687 30118526

[331] E. E. O'Neill, D. Matallanas, and W. Kolch, “Mammalian sterile 20-like kinases in tumor suppression: an emerging pathway:,” *Cancer Research*, vol. 65, no. 13, pp. 5485–5487, 2005 15994916 10.1158/0008-5472.CAN-05-1453

[332] R. Bdeir, W. Muchero, Y. Yordanov, G. A. Tuskan, V. Busov, and O. Gailing, “Quantitative trait locus mapping of *Populus* bark features and stem diameter,” *BMC Plant Biology*, vol. 17, no. 1, article 224, 2017 10.1186/s12870-017-1166-4 PMC5704590 29179673

[333] J. A. McGrath, “Hereditary diseases of desmosomes,” *Journal of Dermatological Science*, vol. 20, no. 2, pp. 85–91, 1999 10379701 10.1016/s0923-1811(99)00015-8

[334] M. J. Chenoweth, K. M. Giacomini, M. Pirmohamed, S. L. Hill, R. H. van Schaik, M. Schwab, A. R. Shuldiner, M. V. Relling, and R. F. Tyndale, “Global pharmacogenomics within precision medicine: challenges and opportunities,” *Clinical Pharmacology & Therapeutics*, vol. 107, pp. 57–61, 2019 31696505 10.1002/cpt.1664 PMC7077926

[335] A. Walia, R. Waadt, and A. M. Jones, “Genetically encoded biosensors in plants: pathways to discovery,” *Annual Review of Plant Biology*, vol. 69, no. 1, pp. 497–524, 2018 10.1146/annurev-arplant-042817-040104 29719164

[336] H. Kollist, S. I. Zandalinas, S. Sengupta, M. Nuhkat, J. Kangasjärvi, and R. Mittler, “Rapid responses to abiotic stress: priming the landscape for the signal transduction network,” *Trends in Plant Science*, vol. 24, no. 1, pp. 25–37, 2019 30401516 10.1016/j.tplants.2018.10.003

[337] M. S. Antunes, S. B. Ha, N. Tewari-Singh, K. J. Morey, A. M. Trofka, P. Kugrens, M. Deyholos, and J. I. Medford, “A synthetic de-greening gene circuit provides a reporting system that is remotely detectable and has a re-set capacity,” *Plant Biotechnology Journal*, vol. 4, no. 6, pp. 605–622, 2006 17309732 10.1111/j.1467-7652.2006.00205.x

[338] M. S. Antunes, K. J. Morey, J. J. Smith, K. D. Albrecht, T. A. Bowen, J. K. Zdunek, J. F. Troupe, M. J. Cuneo, C. T. Webb, H. W. Hellinga, and J. I. Medford, “Programmable ligand detection system in plants through a synthetic signal transduction pathway,” *PLoS One*, vol. 6, no. 1, article e16292, 2011 10.1371/journal.pone.0016292 PMC3026823 21283542

[339] B. P. Teague, P. Guye, and R. Weiss, “Synthetic morphogenesis,” *Cold Spring Harbor Perspectives in Biology*, vol. 8, no. 9, article a023929, 2016 10.1101/cshperspect.a023929 PMC5008072 27270296

[340] R. Wightman, and C. J. Luo, “From mammalian tissue engineering to 3D plant cell culture,” *The Biochemist*, vol. 38, no. 4, pp. 32–35, 2016

[341] C. J. Luo, R. Wightman, E. Meyerowitz, and S. K. Smoukov, “A 3-dimensional fibre scaffold as an investigative tool for studying the morphogenesis of isolated plant cells,” *BMC Plant Biology*, vol. 15, no. 1, article 211, 2015 10.1186/s12870-015-0581-7 PMC4550058 26310239

[342] R. Bernstein, K. Ingram, and K. M. Hart *BioBuilder: Synthetic Biology in the Lab*, O'Reilly Media, Inc., Sebastopol, 2015

[343] B. Llorente, T. C. Williams, and H. D. Goold, “The multiplanetary future of plant synthetic biology,” *Genes*, vol. 9, no. 7, p. 348, 2018 29996548 10.3390/genes9070348 PMC6071031

[344] A. A. Menezes, M. G. Montague, J. Cumbers, J. A. Hogan, and A. P. Arkin, “Grand challenges in space synthetic biology,” *Journal of The Royal Society Interface*, vol. 12, no. 113, pp. 20150803–20150803, 2015 26631337 10.1098/rsif.2015.0803 PMC4707852

[345] S. M. Schmidt, M. Belisle, and W. B. Frommer, “The evolving landscape around genome editing in agriculture,” *EMBO Reports*, vol. 21, article e50680, 2020 10.15252/embr.202050680 PMC7271327 32431018

[346] K. Kawall, J. Cotter, and C. Then, “Broadening the GMO risk assessment in the EU for genome editing technologies in agriculture,” *Environmental Sciences Europe*, vol. 32, no. 1, article 106, 2020

[347] J. A. Paine, C. A. Shipton, S. Chaggar, R. M. Howells, M. J. Kennedy, G. Vernon, S. Y. Wright, E. Hinchliffe, J. L. Adams, A. L. Silverstone, and R. Drake, “Improving the nutritional value of Golden Rice through increased pro-vitamin A content,” *Nature Biotechnology*, vol. 23, no. 4, pp. 482–487, 2005 10.1038/nbt1082 15793573

[348] A. Wakeel, S. Arif, M. A. Bashir, Z. Ahmad, H. . Rehman, A. Kiran, S. Ibrahim, and M. R. Khan, “Perspectives of folate biofortification of cereal grains,” *Journal of Plant Nutrition*, vol. 41, no. 19, pp. 2507–2524, 2018

[349] M.-L. Shih, and J. A. Morgan, “Metabolic flux analysis of secondary metabolism in plants,” *Metabolic Engineering Communications*, vol. 10, article e00123, 2020 10.1016/j.mec.2020.e00123 PMC7031320 32099803

[350] J. A. Nordlee, S. L. Taylor, J. A. Townsend, L. A. Thomas, and R. K. Bush, “Identification of a Brazil-nut allergen in transgenic soybeans,” *New England Journal of Medicine*, vol. 334, no. 11, pp. 688–692, 1996 8594427 10.1056/NEJM199603143341103

[351] M. Nestle, “Allergies to transgenic foods--questions of policy,” *New England Journal of Medicine*, vol. 334, no. 11, pp. 726–728, 1996 8594435 10.1056/NEJM199603143341111

[352] G. U. Ryffel, “Transgene flow: facts, speculations and possible countermeasures,” *GM Crops Food*, vol. 5, no. 4, pp. 249–258, 2014 25523171 10.4161/21645698.2014.945883 PMC5033179

[353] B.-R. Lu, “Transgene escape from GM crops and potential biosafety consequences: an environmental perspective,” *Collection of Biosafety Reviews*, vol. 4, pp. 66–141, 2008

[354] S. Ruf, D. Karcher, and R. Bock, “Determining the transgene containment level provided by chloroplast transformation,” *Proceedings of the National Academy of Sciences*, vol. 104, no. 17, pp. 6998–7002, 2007 10.1073/pnas.0700008104 PMC1849964 17420459

[355] V. Kuvshinov, K. Koivu, A. Kanerva, and E. Pehu, “Molecular control of transgene escape from genetically modified plants,” *Plant Science*, vol. 160, no. 3, pp. 517–522, 2001 11166439 10.1016/s0168-9452(00)00414-3

[356] H. Daniell, “Molecular strategies for gene containment in transgenic crops,” *Nature Biotechnology*, vol. 20, no. 6, pp. 581–586, 2002 10.1038/nbt0602-581 PMC3471138 12042861

[357] A. L. Klocko, H. Lu, A. Magnuson, A. M. Brunner, C. Ma, and S. H. Strauss, “Phenotypic expression and stability in a large-scale field study of genetically engineered poplars containing sexual containment transgenes,” *Frontiers in Bioengineering and Biotechnology*, vol. 6, p. 100, 2018 30123794 10.3389/fbioe.2018.00100 PMC6085431

[358] S. B. Powles, and Q. Yu, “Evolution in action: plants resistant to herbicides,” *Annual Review of Plant Biology*, vol. 61, no. 1, pp. 317–347, 2010 10.1146/annurev-arplant-042809-112119 20192743

[359] Y. Carrière, J. A. Fabrick, and B. E. Tabashnik, “Can pyramids and seed mixtures delay resistance to Bt crops?,” *Trends in Biotechnology*, vol. 34, no. 4, pp. 291–302, 2016 26774592 10.1016/j.tibtech.2015.12.011

[360] J. Young, G. Zastrow-Hayes, S. Deschamps, S. Svitashev, M. Zaremba, A. Acharya, S. Paulraj, B. Peterson-Burch, C. Schwartz, V. Djukanovic, B. Lenderts, L. Feigenbutz, L. Wang, C. Alarcon, V. Siksnys, G. May, N. D. Chilcoat, and S. Kumar, “CRISPR-Cas9 editing in maize: systematic evaluation of off-target activity and its relevance in crop improvement,” *Scientific Reports*, vol. 9, no. 1, article 6729, 2019 10.1038/s41598-019-43141-6 PMC6491584 31040331

[361] D. Carroll, “Collateral damage: benchmarking off-target effects in genome editing,” *Genome Biology*, vol. 20, no. 1, p. 114, 2019 31159845 10.1186/s13059-019-1725-0 PMC6545683

[362] P. E. Abraham, J. L. Labbé, and A. A. McBride, “Advancing how we learn from biodesign to mitigate risks with next-generation genome engineering,” *BioDesign Research*, vol. 2020, pp. 1–3, 2020 10.34133/2020/9429650 PMC10530647 37849898

[363] J. H. Lee, M. Mazarei, A. C. Pfotenhauer, A. B. Dorrough, M. R. Poindexter, T. Hewezi, S. C. Lenaghan, D. E. Graham, and C. N. Stewart Jr., “Epigenetic footprints of CRISPR/Cas9-mediated genome editing in plants,” *Frontiers in Plant Science*, vol. 10, p. 1720, 2020 32117329 10.3389/fpls.2019.01720 PMC7026911

[364] J. E. Losey, L. S. Rayor, and M. E. Carter, “Transgenic pollen harms monarch larvae,” *Nature*, vol. 399, no. 6733, pp. 214–214, 1999 10.1038/20338 10353241

[365] A. Gutmann, “The ethics of synthetic biology: guiding principles for emerging technologies,” *Hastings Center Report*, vol. 41, no. 4, pp. 17–22, 2011 10.1002/j.1552-146x.2011.tb00118.x 21845917

[366] P. Beyer, “Golden Rice and ‘Golden’ crops for human nutrition,” *New Biotechnology*, vol. 27, no. 5, pp. 478–481, 2010 20478420 10.1016/j.nbt.2010.05.010

[367] A. D. Maynard, “Navigating the fourth industrial revolution,” *Nature Nanotechnology*, vol. 10, no. 12, pp. 1005–1006, 2015 10.1038/nnano.2015.286 26632281

[368] M. Xu, J. M. David, and S. H. Kim, “The fourth industrial revolution: opportunities and challenges,” *International Journal of Financial Research*, vol. 9, no. 2, pp. 90–95, 2018

[369] P. Prisecaru, “Challenges of the fourth industrial revolution,” *Knowledge Horizons. Economics*, vol. 8, p. 57, 2016

[370] E. Gonçalves, J. Bucher, A. Ryll, J. Niklas, K. Mauch, S. Klamt, M. Rocha, and J. Saez-Rodriguez, “Bridging the layers: towards integration of signal transduction, regulation and metabolism into mathematical models,” *Molecular BioSystems*, vol. 9, no. 7, pp. 1576–1583, 2013 23525368 10.1039/c3mb25489e

[371] L. Heirendt, S. Arreckx, T. Pfau, S. N. Mendoza, A. Richelle, A. Heinken, H. S. Haraldsdóttir, J. Wachowiak, S. M. Keating, V. Vlasov, S. Magnusdóttir, C. Y. Ng, G. Preciat, A. Žagare, S. H. J. Chan, M. K. Aurich, C. M. Clancy, J. Modamio, J. T. Sauls, A. Noronha, A. Bordbar, B. Cousins, D. C. el Assal, L. V. Valcarcel, I. Apaolaza, S. Ghaderi, M. Ahookhosh, M. Ben Guebila, A. Kostromins, N. Sompairac, H. M. le, D. Ma, Y. Sun, L. Wang, J. T. Yurkovich, M. A. P. Oliveira, P. T. Vuong, L. P. el Assal, I. Kuperstein, A. Zinovyev, H. S. Hinton, W. A. Bryant, F. J. Aragón Artacho, F. J. Planes, E. Stalidzans, A. Maass, S. Vempala, M. Hucka, M. A. Saunders, C. D. Maranas, N. E. Lewis, T. Sauter, B. Ø. Palsson, I. Thiele, and R. M. T. Fleming, “Creation and analysis of biochemical constraint-based models using the COBRA Toolbox v.3.0,” *Nature Protocols*, vol. 14, no. 3, pp. 639–702, 2019 30787451 10.1038/s41596-018-0098-2 PMC6635304

[372] T. W. Grunberg, and D. Del Vecchio, “Modular analysis and design of biological circuits,” *Current Opinion in Biotechnology*, vol. 63, pp. 41–47, 2020 31877425 10.1016/j.copbio.2019.11.015

[373] B. M. Tyler, “The fog of war: how network buffering protects plants’ defense secrets from pathogens,” *PLoS Genetics*, vol. 13, no. 5, article e1006713, 2017 10.1371/journal.pgen.1006713 PMC5417413 28472034

[374] K. Yugi, H. Kubota, A. Hatano, and S. Kuroda, “Trans-omics: how to reconstruct biochemical networks across multiple ‘omic’ layers,” *Trends in Biotechnology*, vol. 34, no. 4, pp. 276–290, 2016 26806111 10.1016/j.tibtech.2015.12.013

[375] G. Zampieri, S. Vijayakumar, E. Yaneske, and C. Angione, “Machine and deep learning meet genome-scale metabolic modeling,” *PLoS Computational Biology*, vol. 15, no. 7, article e1007084, 2019 10.1371/journal.pcbi.1007084 PMC6622478 31295267

